# Beyond capacity limits: can social cohesion offset the impact of service constraints on youth mental health?

**DOI:** 10.1192/j.eurpsy.2025.10053

**Published:** 2025-06-27

**Authors:** Jo-An Occhipinti, Nicholas Ho, Paul Crosland, Sam Huntley, Wendy Hawkins, Adam Connell, Judith Piccone, Sarah Piper, Seyed Hossein Hosseini, Catherine Vacher, Jordan van Rosmalen, Sophie Morson, Courtney Milham, Wendy Burton, Kayla Andrade, Chloe Gosling, Kristen Tran, Yun Ju Christine Song, Victoria Loblay, Jo Robinson, Adam Skinner, Ian B. Hickie

**Affiliations:** 1 Brain and Mind Centre, Faculty of Medicine and Health, University of Sydney, Sydney, Australia; 2 Computer Simulation & Advanced Research Technologies, Sydney, Australia; 3 Metro South Hospital and Health Service, Queensland Health, Brisbane, Australia; 4 Mental Health Alcohol and Other Drugs Strategy and Planning Branch, Queensland Health, Brisbane, Australia; 5 Children’s Health Queensland Hospital and Health Service, Brisbane, Australia; 6 Thriving Queensland Kids Partnership, Australian Research Alliance for Children and Youth, Brisbane, Australia; 7 Brisbane South Primary Health Network, Brisbane, Australia; 8 Morningside General Practice Clinic, Brisbane, Australia; 9 Orygen, Melbourne, Australia; 10 Centre for Youth Mental Health, The University of Melbourne, Melbourne, Australia

**Keywords:** health systems, system dynamics, social cohesion, workforce, youth mental health

## Abstract

**Background:**

Rates of youth anxiety, depression, and self-harm have increased substantially in recent years. Expansion of clinical service capacity is constrained by workforce shortages and system fragmentation, and even substantial investment may not achieve the scale of growth required to address unmet need. Preventive strategies – such as strengthening social cohesion – are therefore essential to alleviate mounting pressures on the mental health system, yet their potential to compensate for these constraints remains unquantified.

**Methods:**

This study employed a system dynamics model to explore the interplay between service capacity and social cohesion on youth mental health outcomes. The model was developed for a population catchment characterized by a mix of urban, suburban, and rural communities. Primary outcomes were prevalence of psychological distress and mental disorders, and incidence of mental health-related emergency department (ED) presentations among young people aged 15–24 years, projected over a 10-year time horizon. Two-way sensitivity analyses of services capacity and social cohesion were conducted.

**Results:**

Changes to specialized mental health services capacity growth had the greatest projected impact on youth mental health outcomes. Heatmaps revealed thresholds where improvements in social cohesion could offset negative impacts of constrained service capacity. For example, if services capacity growth was sustained at only 80% of baseline, improving social cohesion could still reduce years lived with symptomatic disorder by 6.3%. To achieve a similar scale of improvement without improvements in social cohesion, the current growth rate in services capacity would need to be more than double. Combining a doubling of service capacity growth with reversing the decline in social cohesion could reduce ED presentations by 25.6% and years with symptomatic mental disorder by 19.2%. A doubling of specialized, headspace, and GP services capacity growth could prevent 24,060 years lived with symptomatic mental disorder among youth aged 15–24.

**Conclusions:**

This study provides a quantitative framework for understanding how social cohesion improvements can help mitigate workforce constraints in mental health systems, demonstrating the value of integrating service expansion with social cohesion enhancement strategies.

## Introduction

The state of youth mental health globally presents a grave and immediate concern. According to the 2019 Global Burden of Disease study, almost 14% of young people aged 15–24 live with a diagnosable mental disorder, and 24.9% of the years of life lived in disability due to mental ill-health occur before the age of 25 [1]. While the pandemic exacerbated youth mental health challenges, trends had been deteriorating prior. In the US, major depressive episodes increased by 52% among adolescents aged 12– 17 over the period 2005–2017 and 63% among 18–25-year-olds between 2009 and 2017 [2]. In the UK, the incidence rates for depression, anxiety, and eating disorders have all risen substantially between 2003 and 2018 [3]. Similarly, in Australia, the National Study of Mental Health and Wellbeing (2020–2022) reported that almost 40% of young Australians aged 16–24 years have experienced a mental health condition over the previous year, up from 26% in 2007 [4]. Between 2008–2009 and 2021–2022 self-harm hospitalisations among 15–19-year-olds also rose from 245.6 to 389.1 per 100,000 population [5], along with a more than three-fold increase in self-harm hospitalisations in females under the age of 14 [5].

Despite the elevation of mental health in the global development agenda [6–10], there has been no global reduction in the burden of mental disorder over the past three decades [11]. Recent examinations of this lack of progress point to increases in treatment provision being insufficient to offset a concurrent increase in the incidence of psychological distress and disorder driven by broader economic and social factors [12, 13]. To address the escalating youth mental health challenge, calls for investments to grow mental health services capacity (whether through increases to the overall workforce or increases in services efficiency or both) to address unmet need have been made by global institutions [14]. Unmet need is often perceived as a static quantum; however, it is dynamic and depends on service-capacity growth relative to (a) population growth; (b) the rising incidence of youth mental health conditions; and (c) progression from mild to severe disorders due to inadequate or delayed care. Efforts to expand service capacity are further constrained by significant skilled workforce shortages due to workforce aging, burnout, staff turnover, and quality and training concerns. The Australian Institute of Health and Welfare (AIHW) reported that approximately 26% of psychologists (2022) and over 40% of psychiatrists (2023) are aged 55 and over, indicating that a significant proportion of the workforce will retire over the coming decade [15]. Burnout and staff turnover further complicate the scenario, as the emotional toll of working in mental health and rising caseload intensity can lead to early retirement or career changes. This was further exacerbated by the COVID-19 pandemic [16]. Additionally, there are challenges in attracting and recruiting new professionals into the mental health field due to precarious work arrangements, insufficient remuneration and support structures, and lack of career pathways [17]. Quality and training concerns also persist, as the demand for mental health services often outstrips the availability of well-trained professionals [17].

In the face of these systemic limits – where capacity expansion alone cannot keep pace with dynamic unmet need – it becomes critical to moderate both the onset and progression of youth mental disorders by enhancing the environments in which young people live, including their communities, schools, and social networks [18]. In particular, social cohesion within these environments can foster a sense of belonging, support, and resilience. Social cohesion is best conceptualized as “a state of affairs concerning both vertical and horizontal interactions among members of society as characterized by a set of attitudes and norms that includes trust, a sense of belonging and the willingness to participate and help, as well as their behavioural manifestations.”[19] The Scanlon–Monash Index operationalizes this concept through five core domains – belonging, worth, social justice, participation, and acceptance/rejection – providing a comprehensive framework for measuring and understanding how communities maintain social bonds [20]. Evidence indicates that strong social bonds within communities can protect against the development of mental health issues such as depression, anxiety, and suicidal ideation among adolescents [21]. Social cohesion also moderates the impacts of other social determinants such as economic deprivation on mental health, providing a sense of belonging and access to resources [22, 23]. Therefore, leveraging social cohesion presents a promising complementary strategy to mitigate the growing youth mental health challenge and alleviate pressure on mental health systems, while investments in services capacity seek to address current unmet needs.

While investing in growth in services capacity and building social cohesion and support for young people may seem to be obvious strategies, what remains unknown is the extent to which different levels of each in particular contexts might result in an improvement in youth mental health outcomes at a population level. Therefore, this study aims to answer two key research questions: (i) Which services capacity increases are likely to deliver the greatest impacts on the prevalence of psychological distress, symptomatic mental disorder, and mental health related emergency department presentations in young people; and (ii) Can improvements in social cohesion help offset the impacts of constraints in mental health service capacity growth? To answer these questions, we employed a system dynamics model developed for an Australian population catchment (Brisbane South Primary Health Network) that captured service demand/supply dynamics and key social drivers of youth mental health.

## Method

### Context, model structure, outputs, and calibration

The Brisbane South Primary Health Network (PHN) is a large catchment covering 3,770 square kilometres with a diverse and rapidly growing population of approximately 1.2 million people (2024), and characterised by a mix of urban, suburban, and rural communities. The population catchment is characterised by substantial cultural diversity – with over 30% of residents born overseas (19.4% from non-English–speaking backgrounds), and 2% identifying as Aboriginal and Torres Strait Islander – as well as marked socioeconomic gradients (15.6% in the most disadvantaged quintile; 32.1% of low-income households experiencing housing stress), low educational attainment (29.3% of adults without schooling beyond Year 10) and elevated social risk factors including risky alcohol intake (15%), illicit drug use in the previous 12 months (16%) and homelessness (52.7 per 10,000) [24]. The region faces a substantial burden from mental disorders with a prevalence of 39.3% among 16–24 years olds in 2022 [25], and a significantly higher rate of intentional self-harm hospitalisations of 217 per 100,000 population in 2019–2020 compared to the national average of 136 per 100,000 population [24]. This is compounded by ongoing challenges of insufficient resources for mental health services for children and young people, workforce shortages, and system fragmentation and complexity.

System dynamics modelling is an analytical approach that uses systems of coupled differential equations, underpinned by well-established mathematical theory of nonlinear dynamics, to simulate and analyse the behaviour of complex systems over time. The participatory model development approach is described in detail elsewhere [26]. To summarise, the process comprised three full‐day workshops and additional technical meetings with a multidisciplinary group including service providers, managers and planners, educators, community and NGO representatives, and young people with lived experience of mental health issues. In these sessions, stakeholders mapped the flows, barriers, and feedback loops of the local youth mental health and broader social systems as well as verified and refined data inputs and model assumptions to align with real‐world conditions. In addition, stakeholders engaged in scenario testing, reviewing model outputs, validating its behaviour, and ensuring its practical relevance for local policy and planning.

**Figure 1.**
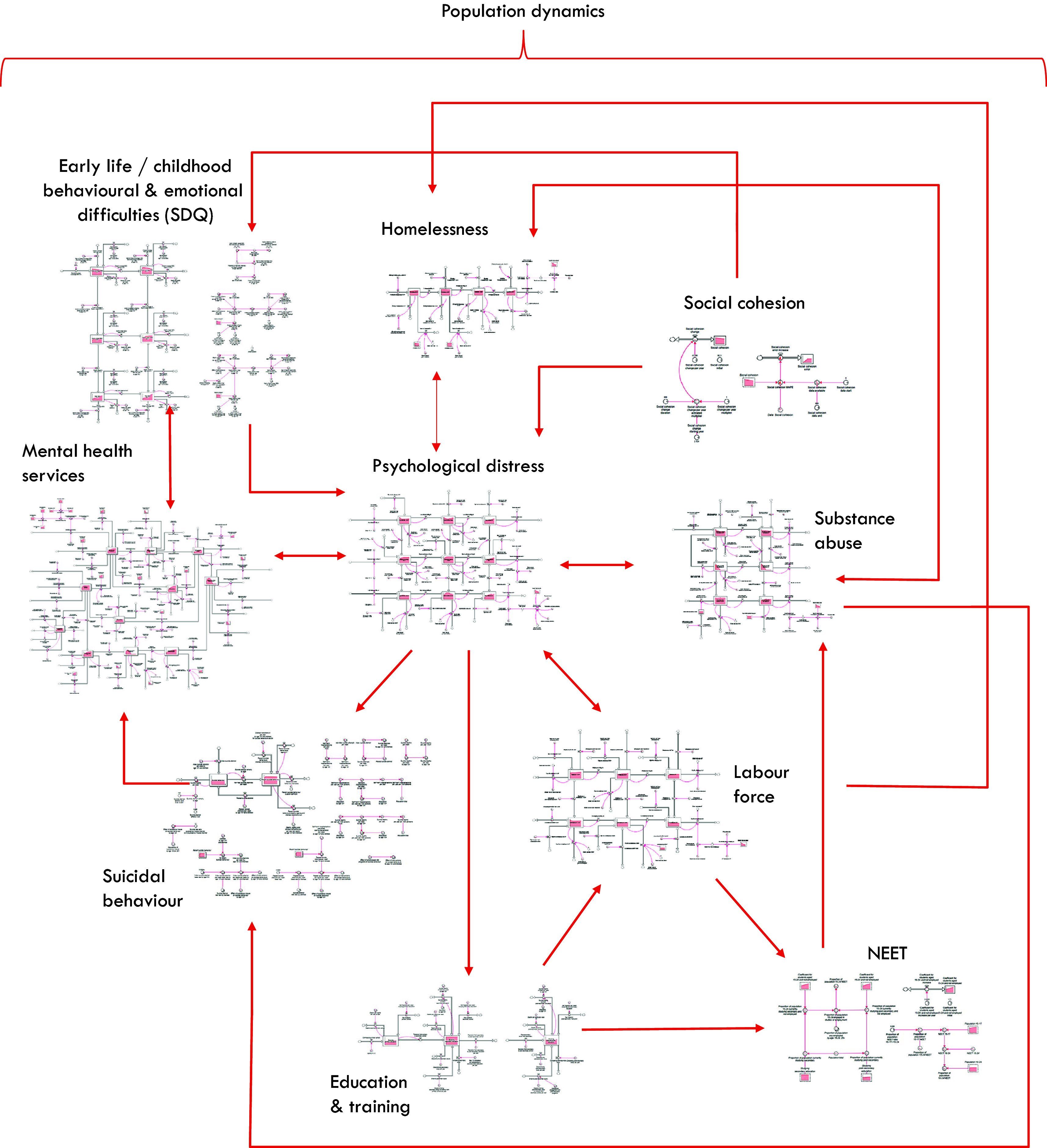
Overview of the causal structure of the system dynamics model. Arrows denote unidirectional or bidirectional relationships between each component based on research evidence and data. NEET: Youth not in employment, education, or training. Population growth influences all sectors of the model.

Figure 1 provides a high-level overview of the causal structure and pathways of the system dynamics model used for our analyses. Arrows denote unidirectional or bidirectional relationships between each component based on research evidence and data outlined in Table 1. Parameterisation and calibration of the model drew on a broad range of data sources including demographic and administrative records from the Australian Bureau of Statistics, Queensland Health, Australian Institute of Health and Welfare, and Brisbane South PHN (including population statistics, births, deaths, migration data, hospital admissions, emergency department presentations, and Medicare-subsidised services), nationally representative surveys (including National Health Surveys, National Study of Mental Health and Wellbeing, Young Minds Matter Survey, National Drug Strategy Household Survey, and the Longitudinal Study of Australian Children), and supplemented by research evidence from longitudinal studies, meta-analyses, systematic reviews, and prospective cohort studies. A full description of model structure, input data sources, and results of calibration of each sector is detailed in Supplementary file 1. To summarise, the model included the following sectors:

- A population component, capturing changes over time in the size and composition of the population resulting from births, migration, ageing, and mortality, and with a focus on young people aged 15–24 years.
- A psychological distress component that models the prevalence of and flows between states of (i) low psychological distress (Kessler 10 [K10] [27] score 10–15), (ii) moderate-to-very-high psychological distress (K10 score 16–50) with no mental disorder, and (iii) moderate-to-very-high psychological distress with symptomatic mental disorder.
- An early life vulnerability sector, which models the prevalence of behavioural and emotional difficulties among children under 12 years of age as measured by the Strengths and Difficulties Questionnaire (SDQ) [28].
- A series of components capturing the interdependent dynamics of key social determinants relevant to the modelled region, namely, substance misuse, homelessness, unemployment and underemployment, education and training, youth not in employment, education or training (NEET), social cohesion (according to the Scanlon-Monash Index of Social Cohesion [20]), and their influence on the onset of psychological distress and symptomatic mental disorder.
- A mental health services component that models the movement of people seeking care through any of several possible youth mental health service pathways involving (potentially) general practitioners, headspace (community-based, early intervention for mild-to-moderate youth mental health issues), specialised services (i.e., psychiatrists, psychologists and allied mental health professionals), community mental health care services (ambulatory care/hospital-based outpatient psychiatric services including child and youth mental health services (CYMHS) for those under the age of 18 years), emergency departments and psychiatric inpatient care, and online services. Waiting for services and services disengagement were also modelled, each reflecting changes over time in the interaction between service demand and service capacity.
- A suicide-related behaviour component that captures self-harm hospitalisations and suicide deaths.

**Figure 2.**
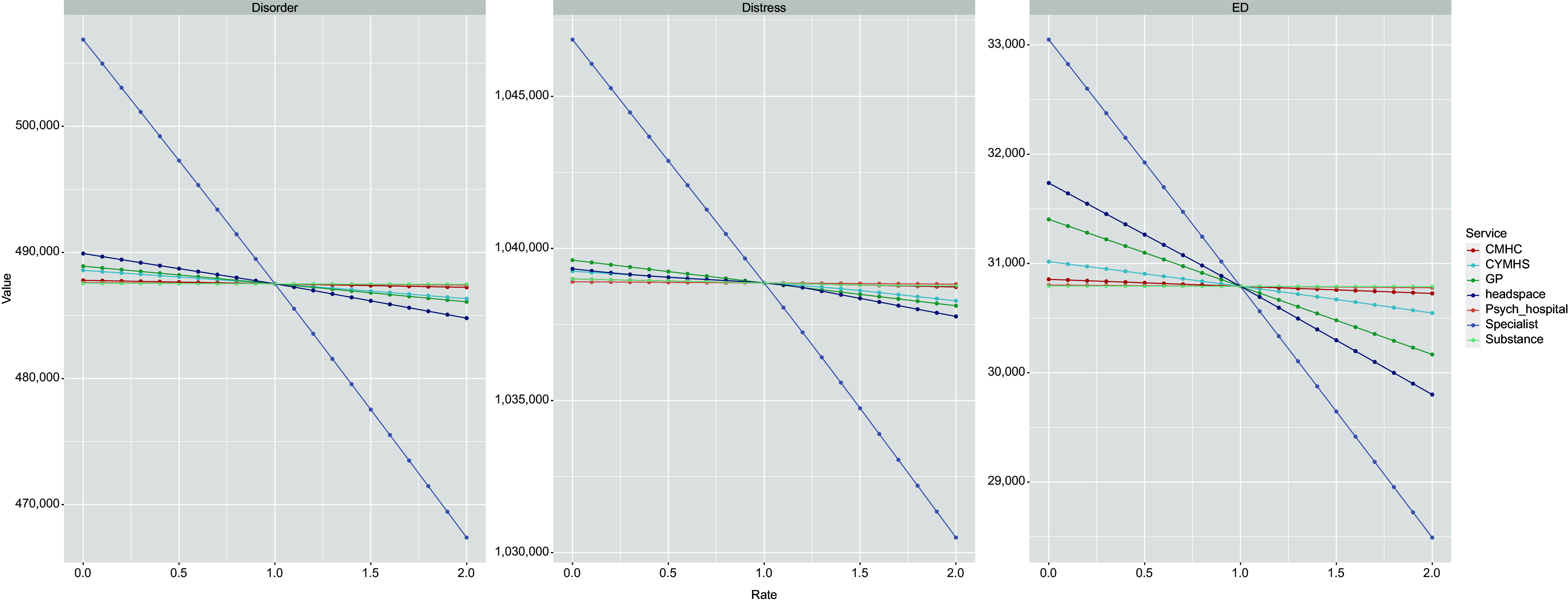
Simulation results. Panel A shows impact of changes to growth rate of services capacity (x-axis) on cumulative years with symptomatic mental disorder (y-axis). Panel B shows impact on cumulative years in moderate to high psychological distress. Panel C shows impact on cumulative mental health-related emergency department presentations. Values less than 1 on the x-axis represent a reduction in the baseline services growth rate, values greater than 1 represent an increase in the baseline services growth rate. CMHC: State funded Community Mental Health Care services; CYMHS: Child and Youth Mental Health Services; GP: General Practitioner – mental health related services; Psych Hospital: Tertiary services (specifically as provided by mental health inpatient units in general hospitals); Specialist: Specialised mental health services (psychiatrists, psychologists and allied mental health services); Substance: Alcohol and other drug treatment services.

**Table 1.** Parameters and data sources informing the interconnections among model sectors

Variable name	Value		Citation
**Education**			
Effect of moderate to very high distress on discontinuation of secondary education	1.99		Derived from Butterworth and Leach [30]
Effect of psychological distress on discontinuation of post-secondary education	1.1		Derived from Lee, Tsang, Breslau, Aguilar-Gaxiola, Angermeyer, Borges [31]
**Labour force**			
Effect of post-secondary qualification on underemployment to sufficiently employed rate	1.407		Derived from Wilkins [32]
Effect of moderate to very high distress on employment	0.840		Derived from Frijters, Johnston and Shields [33]
Effect of post-secondary qualification on participation	1.436		Derived from Australian Bureau of Statistics [34]
Effect of post-secondary qualification on employment vs low educational attainment	1.512		Derived from Australian Bureau of Statistics [34]
Effect of secondary qualification only on participation	1.281		Derived from Australian Bureau of Statistics [34]
Effect of secondary qualification only on employment vs low educational attainment	1.305		Derived from Australian Bureau of Statistics [34]
**Psychological distress/disorder**			
Effect of unemployment on distress	15–24 years	1.43	Derived from Australian Bureau of Statistics [35]
	25 years and older	1.81	
Effect of homelessness on distress	2.14		Derived from Australian Bureau of Statistics [35]
Effect of underemployment on distress	1.132		Derived from Dooley, Prause and Ham-Rowbottom [36]
Effect of substance abuse on distress	2.63		Derived from Marmorstein, Iacono and Malone [37]
Proportion of close to average SDQ to low distress	0.468		Derived from Australian Institute of Family Studies [38]
Proportion of close to average SDQ to disorder, moderate to very high distress	0.238		Derived from Australian Institute of Family Studies [38]
Proportion of slightly raised SDQ to low distress	0.182		Derived from Australian Institute of Family Studies [38]
Proportion of slightly raised SDQ to disorder, moderate to very high distress	0.62		Derived from Australian Institute of Family Studies [38]
Proportion of high SDQ to low distress	0.112		Derived from Australian Institute of Family Studies [38]
Proportion of high SDQ to disorder, moderate to very high distress	0.72		Derived from Australian Institute of Family Studies [38]
**Homelessness**			
Effect of unemployment on entering homelessness	2.6		Derived from Nilsson, Nordentoft and Hjorthøj [39]
Effect of mental illness on entering homelessness	1.7		Derived from Nilsson, Nordentoft and Hjorthøj [39]
Effect of substance misuse on entering homelessness	2.3		Derived from Nilsson, Nordentoft and Hjorthøj [39]
**Substance misuse**			
Effect of homelessness on substance misuse	1.65		Derived from Johnson, Freels, Parsons and Vangeest [40]
Effect of moderate to very high distress on substance misuse	2.505		Derived from Australian Institute of Health and Welfare [41]
Effect of NEET substance misuse	1.43		Derived from Gariépy, Danna, Hawke, Henderson and Iyer [42]
**Social cohesion**			
Coefficient social cohesion on distress onset per year per base rate by age 12+	12–14 years	−0.107	Estimated via constrained optimisation using the Scanlon-Monash Index of Social Cohesion data from the Scanlon Foundation Research Institute [20]
	15–17 years	−0.020	
	18–24 years	−0.028	
	25 years and older	−0.0329	
Intercept social cohesion on distress onset per year per base rate by age 12+	12–14 years	0.049	Estimated via constrained optimisation using the Scanlon-Monash Index of Social Cohesion data from the Scanlon Foundation Research Institute [20]
	15–17 years	0.061	
	18–24 years	−0.002	
	25 years and older	0.034	
Coefficient social cohesion on disorder incidence per year base rate by age 12+	12–14 years	−0.1176	Estimated via constrained optimisation using the Scanlon-Monash Index of Social Cohesion data from the Scanlon Foundation Research Institute [20]
	15–17 years	−0.027	
	18–24 years	−0.028	
	25 years and older	−0.026	
Intercept social cohesion on disorder incidence per year base rate by age 12+	12–14 years	0.974	Estimated via constrained optimisation using the Scanlon-Monash Index of Social Cohesion data from the Scanlon Foundation Research Institute [20]
	15–17 years	0.993	
	18–24 years	1.014	
	25 years and older	0.980	
**Suicidal behaviours**			
Suicide rate ratio of substance misuse disorder vs no substance misuse disorder	4.1		Derived from Too, Spittal, Bugeja, Reifels, Butterworth and Pirkis [43]
Suicide attempt rate ratio by distress	No distress	1	Derived from Hockey, Rocks, Ruusunen, Jacka, Huang, Liao [44]
	Distress no disorder	1.41	
	Distress disorder	3.57	
**Services**			
Effect of disengagement on increasing psychological distress	1.271517		Derived from Australian Bureau of Statistics [45]
Effect of distress on hospitalisation	No distress	0	Derived from Australian Bureau of Statistics [35]
	Distress no disorder	1.41	
	Distress disorder	3.57	
Effect of distress on help-seeking with GP	No distress	0	Derived from Australian Bureau of Statistics [35]
	Distress no disorder	1	
	Distress disorder	3.287	
Effect of distress on referral rate	No distress	0	Derived from Australian Bureau of Statistics [35]
	Distress no disorder	1	
	Distress disorder	1.786	
Effect of distress on seeking help psychiatrist or allied health services	No distress	0	Derived from Australian Bureau of Statistics [35]
	Distress no disorder	1	
	Distress disorder	4.398	
Baseline recovery rate psychiatric hospital care	No distress	0	Derived from Thase, Greenhouse, Frank, Reynolds, Pilkonis, Hurley [46]
	Distress no disorder	0.424	
	Distress disorder	0.371	
Baseline recovery rate headspace	0.050295858		Derived from KPMG [47]
Baseline recovery rate CMHC services	0.02332282		Derived from Australian Institute of Health and Welfare [48]
Recovery rate ratio GP services	No distress	0	Derived from Cuijpers, Straten, Schaik and Andersson [49]
	Distress no disorder	1	
	Distress disorder	0.463	
Baseline recovery rate mental health treatment	No distress	0	Derived from Thase, Greenhouse, Frank, Reynolds, Pilkonis, Hurley [46]
	Distress no disorder	0.095	
	Distress disorder	0.083	
Recovery rate online services	No distress	0	Derived from Cuijpers, Straten, Schaik and Andersson [49], Christensen, Griffiths and Jorm [50]
	Distress no disorder	0.4	
	Distress disorder	0.185	

Primary model outputs for these analyses were (i) prevalence of, and cumulative years lived in moderate-to-very-high psychological distress (youth aged 15–24 years), (ii) prevalence of, and cumulative years lived with symptomatic mental disorder (youth aged 15–24 years), and (iii) incidence of mental health-related emergency department (ED) presentations (youth aged 15–24 years). All outputs were calculated every 0.875 days (i.e. one eighth of a week) over a period of 24 years, starting from 1 January 2011, permitting comparisons of model outputs with historic data from 2011 to 2023 (see Additional file 1) and forecasts of the impacts of scenarios described below simulated from the time of implementation (2025) to 1 January 2035. Parameter values that could not be derived directly from available data or published research were estimated via constrained optimisation, using historical time series data on a wide range of mental health and social outcomes, including the prevalence of psychological distress, rates of mental health services usage, self-harm hospitalisation and suicide rates, prevalence of substance use disorder, rates of labour underutilisation, and the prevalence of homelessness. Powell’s method [29] was employed to obtain the set of (optimal) parameter values minimising the sum of the mean absolute percent error calculated for each time series separately (i.e. the mean of the absolute differences between the observed time series values and the corresponding model outputs, where each difference is expressed as a percentage of the observed value).

## Scenario analyses

To answer research question (i), we modelled scenarios of both increasing and decreasing rate of growth in capacity of each category of youth mental health services and explored their projected impacts on cumulative years spent in moderate-to-very-high psychological distress, cumulative years spent with symptomatic mental disorder, and cumulative mental health-related emergency department (ED) presentations among youth aged 15–24 years. Differences in projected outputs between baseline and scenario runs were calculated for each growth rate multiplier increment (0.1) spanning 0 (no growth) to 2 (double the baseline growth rate). The baseline annual capacity growth for each service was modelled as a yearly linear increase calibrated using AIHW data on mental health‐specific services. Table 2 describes each service with an absolute annual increase under baseline and maximum growth rate scenarios.

**Table 2.** Service descriptions, parameter values, and data sources used in the model analysis

Service	Description and data sources	Baseline/default growth per year (absolute)	Maximum growth per year (absolute) i.e. double the baseline growth rate
General practitioner mental health services	Baseline projected growth rate is based on Medicare-subsidised services data published by AIHW for the period 2017 to 2020 (pre-pandemic) [51]. We assume that GP mental health services were operating at maximum capacity over this period.	95.2 additional services/week	190.4 additional services/week
Specialist mental health services	Baseline projected growth rate is based on Medicare-subsidised services data published by AIHW for the period 2017 to 2020 (pre-pandemic) [51]. We assume that psychiatry, psychology, and allied mental health services were operating at maximum capacity over this period.	289 additional services/week	578.1 additional services/week
Child and youth mental health services (CYMHS)	Baseline projected growth rate is based on Community Mental Health Care Services data for 0–17 year olds published by AIHW for the period 2016 to 2018 (pre-pandemic) [52]. We assume that CYMHS were operating at maximum capacity over this period.	36.5 additional services/week	72.9 additional services/week
Community mental health care services (CMHCs)	Baseline projected growth rate is based on Community Mental Health Care Services data for 18+ year olds published by AIHW for the period 2016 to 2018 (pre-pandemic) [52]. We assume that CMHC services were operating at maximum capacity over this period.	15.5 additional services/week	31.1 additional services/week
Headspace	Baseline projected growth rate is based on headspace occasions of service data provided by BSPHN for the period 2017 to 2022. Weassume that headspace services were operating at maximum capacity over this period.	26 additional services/week	52.2 additional services/week
Psychiatric admitted care	Baseline projected growth rate is based on episodes of admitted patient care provided by Queensland Health [53] for the period 2017 to 2019 (pre-pandemic). We assume that services were operating at maximum capacity over this period.	0.3 additional services/week	0.6 additional services/week
Substance misuse treatment services	Baseline projected growth rate is based on alcohol and other drug closed treatment episodes published by AIHW [54] for the period 2017–18 to 2019–2020 (pre-pandemic). We assume that substance misuse treatment services were operating at maximum capacity over this period.	7 additional services/week	13.9 additional services/week

To answer research question (ii), we combined the services for which increases in capacity growth were projected to have the largest effects on population mental health (ranking is the same for all three outcomes measures) and conducted two-way sensitivity analyses using combinations of services capacity growth scenarios and changes to the historic trend of social cohesion. The historic decline in social cohesion in Australia between 2007 and 2022 has been 16.9% according to the Scanlon-Monash Index of Social Cohesion [20]. The two-way sensitivity analysis used 441 combinations of values. For social cohesion, the multiplier ranged from 1 (which assumes the historic decline in social cohesion will continue) to −1, which reverses the historic decline to a positive increase of equal scale, using increments of 0.1. For services capacity growth scenarios, the multipliers ranged from 0 for no growth to 2 for double the historic growth rate, using increments of 0.1. A heatmap was generated to visualise the conditions under which the projected outcomes would fall below the baseline trajectory (indicating an improvement in youth mental health outcomes). All forward projections were undertaken over a 10-year time horizon, from January 2025 to January 2035. Model construction and analyses were performed using Stella Architect ver. 3.4 (www.iseesystems.com). R statistical package was used for graphical presentation of results.

## Results

Research question (i): Figure 2 shows simulation results for the incremental changes in the growth rate of capacity of each category of youth mental health service and their projected impacts on cumulative years spent in moderate-to-very-high psychological distress, cumulative years spent with symptomatic mental disorder, and cumulative mental health-related ED presentations among young people aged 15–24 years. These results demonstrate that changes to the growth rate in specialised mental health services capacity are projected to have the greatest impact on the outcomes of interest, followed by headspace and GP services, and that all no-growth scenarios were projected to result in a deterioration of youth mental health outcomes.

Mental health-related ED presentations were the health outcome most responsive to changes in service capacity growth rates, showing a 14.8 percentage point difference in scenarios involving specialized mental health services, a 6.3 percentage point difference in scenarios involving headspace service capacity, and a 4-percentage point difference in scenarios involving mental health-related GP services (comparing no growth to doubling the growth rate in service capacity) (Table 3). Cumulative years spent in moderate-to-very-high psychological distress was the health outcome least responsive to changes in service capacity growth rates, with impacts below 1% across the service types. Doubling the growth rate in capacity of specialised, headspace, and GP services has the potential to prevent 24,060 years lived with symptomatic mental disorder among those aged 15–24 years in the Brisbane South population catchment.

**Table 3.** Simulation results. Impact of changes in services capacity growth rates on mental health related ED presentations, cumulative years in moderate-to-very-high psychological distress, and symptomatic mental disorder over the period January 2025 to January 2035

		Mental health-related ED presentations		Cumulative years in moderate-to-very-high psychological distress		Cumulative years with symptomatic mental disorder	
		*n*	%	*n*	%	*n*	%
Specialised services	No services growth	2,258	7.33	7,999	0.77	19,363	3.97
	Double services growth rate	−2,299	−7.47	−8,369	−0.81	−20,118	−4.13
headspace	No services growth	946	3.07	465	0.04	−2,403	0.49
	Double services growth rate	−990	−3.22	−1,101	−0.11	−2,719	−0.56
General Practitioners	No services growth	613	1.99	750	0.07	1,395	0.29
	Double services growth rate	−624	−2.03	−749	−0.07	−1,427	−0.29

Note: All figures are changes in outcome relative to the baseline services capacity growth rates. Negative values indicate an improvement in youth mental health outcomes.

**Figure 3.**
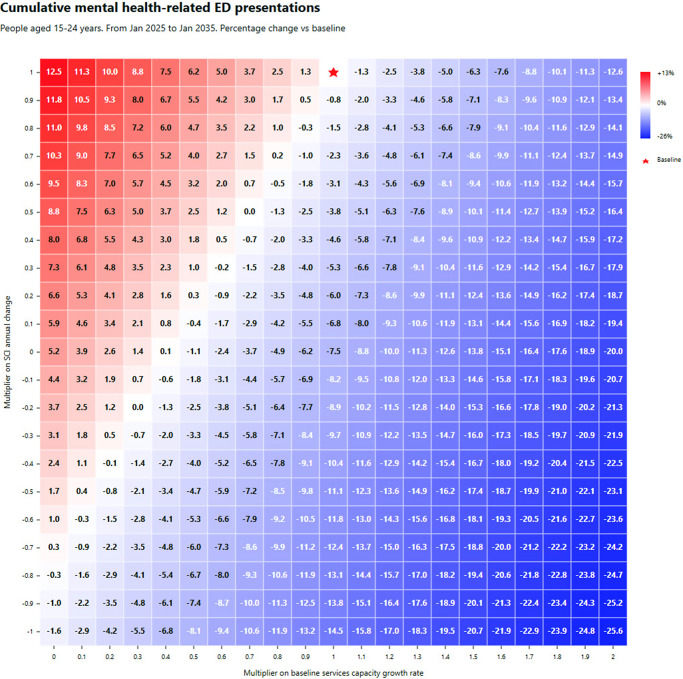
Simulation results. Combined impact of changes to growth rate of specialised services, headspace and GP services capacity (x-axis) versus social cohesion (y-axis) on cumulative mental health-related ED presentations over the 10-year period, January 2025 to January 2035. The figure presented in each square represents the increase/decrease in cumulative mental health-related ED presentations against the baseline. Red shading corresponds with a deterioration (increasing presentations) and blue shading with an improvement (decreasing presentations), with the intensity of shading reflecting the scale of deterioration or improvement. The baseline services capacity growth rate and social cohesion are marked by the red star.

Regarding research question (ii), Figure 3 shows percentage changes in cumulative mental health-related ED presentations as a function of the services capacity growth/degrowth (with specialised services, headspace, and GP services combined) and social cohesion multipliers. Results for cumulative years spent in moderate-to-very-high psychological distress and disorder can be found in Supplementary file 2. Results for all three outcomes demonstrate a threshold beyond which improvements in social cohesion can offset the potentially negative impacts of constraints in service capacity growth over the 10-year time horizon. For example, if the growth in services capacity could only be sustained at 80% of the baseline rate, a cessation in the decline of social cohesion could reduce mental health-related ED presentations by 4.9% and cumulative years lived with symptomatic mental disorder by 6.3%. The figure also shows that if the current decreasing trend in social cohesion continues, a 40% increase in the baseline services capacity growth rate (of specialised services, headspace and GP services) would be required to achieve that modest 4.9% improvement in mental health-related ED presentations, and more than doubling of the growth rate in services capacity (of the same three services) would be needed to achieve the 6.3% improvement in cumulative years lived with symptomatic mental disorder. These heatmaps highlight the potential worsening of youth mental health outcomes under scenarios of continued declines in social cohesion and constraints in the growth of services capacity. They also highlight the scale of improvements that could be achieved if a combined doubling of the growth rates of services capacity and a reversal of the declining trend in social cohesion was feasible i.e., a 25.6% reduction in mental health related ED presentations, a 5.7% reduction in cumulative years lived in moderate-to-high psychological distress, and a 19.2% reduction in cumulative years live with symptomatic mental disorder.

## Discussion

The aim of this study was to understand the future implications of constraints in the growth of youth mental health services capacity (due to resource limitations and workforce shortages) and the extent to which the negative effects of these constraints on mental health related ED presentations and the prevalence of psychological distress and symptomatic mental disorder could be offset by improvements in social cohesion. Findings highlight the critical role primary care and specialised mental health services play within the youth mental health care system in driving key population-level youth mental health outcomes. Importantly, the study reveals the projected scale of negative impacts on these outcomes should constraints in funding and workforce mean that the historic growth rate in services does not continue. These findings are also consistent with previous regional mental health systems modelling, which demonstrates that constraints in services capacity, whether due to population growth, increases in the onset of psychological distress and symptomatic mental disorder, or a result of unintended consequences of programs that effectively drive more people into a service system (e.g., awareness campaigns, community education, helplines), lead to increased waiting times for services, increased disengagement due to the longer waiting times, and a consequent rise in presentations to emergency departments [55, 56].

The findings of this study also support the argument that a focus on either the expansion of services or on improvements in social cohesion in isolation misses an opportunity to achieve substantial improvements in youth mental health outcomes [57]. Importantly, this research highlights the existence of thresholds, points at which reductions in either social cohesion or growth in services capacity (or both) would result in increases in the prevalence of psychological distress, symptomatic mental disorder, and presentations to emergency departments. Quantification of this threshold allows youth mental health funders and service planners to remain responsive to changes in unmet need, encouraging greater collaboration with the social sector to implement a multifaceted approach, coordinating and calibrating real-world investments in fostering social cohesion and growing youth mental health services capacity. Box 1 provides a series of reflections and insights by key system stakeholders engaged as partners in this research.

Box 1. Local stakeholder reflections on the implications of these findings for youth mental health investments, partnerships, advocacy, and action.

**Hospital and health services/service development perspectives:**

WH: These results highlight the importance of establishing and maintaining broad and effective partnerships with sectors related to social determinants (i.e. beyond health) to progress means for addressing the needs of young people, including through preventative approaches such as improving social cohesion.

JP: The findings in this paper demonstrate how a locally developed system dynamics modelling tool can provide a shared language for strengthening partnerships (between Hospital and Health Services and other stakeholders). This can then support an agreement on common goals (such as improving social cohesion) with shared understanding on where quantifiable improvements could be expected in mental health (and broader health) outcomes for young people and their family/community.

**Systems planning perspective**

AC: The findings can help guide ongoing investment into youth mental health as they explore the pressure points in the system. With a deeper understanding of the potential stressors on mental health services, planners and funders can better target investment for improved health outcomes for consumers. This paper highlights how a well-connected mental health system is vital to ensuring that young people are receiving mental health care appropriate to their needs.

**Services commissioning perspective**

CM: The findings identified in this paper show the importance of specialised services, along with a focus on enhancing social cohesion, to meet the needs of young people within a specific region. Additionally, this work highlights the value and importance of successful engagement for collaborative partnerships. Being able to review and evaluate the wider service offerings through the region-specific system dynamics modelling tool, in conjunction with these partnerships, supports the prioritisation and commissioning of health services tailored to the needs of young people in the region.

**Youth lived experience perspectives**

JVR: For young people like myself, the service demand and service capacity issues faced by the mental health sector can have very real, and very damaging impacts. Finding innovative solutions to service capacity issues is fundamental to ensuring young people have access to the services we desperately need. As a person with lived and living experiences of mental distress, I can attest to the value of social cohesion in a person’s recovery journey. Feeling connected to a community of like-minded peers, having a sense of purpose, contributing to issues larger than my own, and feeling confident that I can live a life with meaning are essential to keeping well in an increasingly nihilistic social landscape. Investing in programs that address social cohesion in addition to appropriately funding specialist mental health services for young people provides a clear way forward to achieve better outcomes for my peers.

KA: Reflecting on the results, volunteering at my local mental health service hub has enhanced social relationships in the community. It facilitates conversations about the various gaps in the community while interacting with your locality. During my time at the centre, there were council-funded programs, particularly for the youth, that provided a means of social interaction among young people; this served as a starting point for them to engage in the community. These social activities help them connect, improve wellbeing, learn something new, and stay connected amongst their peers. Unfortunately, you cannot witness the long-term effects come to fruition because funding for these events and social gatherings is frequently cut short. Investing in long-term projects that strengthen social cohesion and youth participation would be extremely beneficial to the future of young people.

**NGO/Systems perspective**

SM: These insights reinforce the need to take a wider perspective on mental illness in their context of the daily lives of young people – in their families and communities, interacting with systems as well as natural, built, and digital environments. This ecological approach has been adopted by The Nest,[63] Australia’s wellbeing framework for children and young people up to 24 years old. It conceptualises wellbeing as six inter-related domains: feeling valued, loved, and safe; being healthy, learning; participating; having material basics; and possessing a strong sense of identity and culture. To have the best possible wellbeing, a young person needs to be adequately resourced in all six domains at an individual level as well as within their family, community, and wider society, including online. Enhancing social cohesion as a means of preventing and/or alleviating youth mental illness (as well as fostering wellbeing in its own right) is therefore the responsibility of many, and requires a multi-faceted systems approach [64]. This will include a paradigm shift towards better addressing upstream factors that impact social cohesion, as well as ensuring equitable responses according to need, so that young people can thrive no matter where they live.

Finally, the findings underscore the urgency of developing strategies to enhance social cohesion. While no specific programs were modelled, scenarios of being able to arrest and possibly reverse the declining trend in social cohesion in Australia since 2007 (a 16.9% decline) were shown to be critical in improving population youth mental health outcomes and alleviating pressure on mental health systems. Initiatives that encourage peer support, mentorship, and community engagement offer promise in creating a protective environment for young people, reducing the likelihood of them developing mental health disorders. These might include youth-led community initiatives, intergenerational engagement, and participation in arts and sports programs. Social prescribing initiatives that connect young people to community resources are being increasingly explored as potential mechanisms to enhance social connections [58]. School-based programs focusing on social-emotional learning could particularly benefit younger adolescents, while employment pathways combined with peer support networks may better address the needs of older youth transitioning to adulthood. Drawing on Putnam’s concept of ‘bridging social capital’ [59], the most effective interventions likely connect young people across different social groups, creating networks that transcend socioeconomic, cultural, and geographic boundaries. Evidence suggests that participation in socially productive activities like volunteering provides numerous direct benefits for young people, including improved well-being, mental health, pro-social behaviour, and life satisfaction, observed in both cross-sectional and longitudinal studies [60, 61]. These activities offer meaningful social roles and the opportunity to develop a positive self-concept, empathy, a sense of purpose, and community connectedness. Determining the feasibility and nature, targeting, scale, and duration of programs needed to improve social cohesion is best explored in partnership with local communities and supported by systems modelling. Local governments can further facilitate these efforts through investments in community infrastructure, public spaces, and targeted funding for organizations working across traditional sectoral boundaries.

There is also a critical role for state and federal governments, given the influence of economic wellbeing, macroeconomic policies, and democratic institutions on social cohesion and mental health [13, 62]. Policies and interventions aimed at reducing poverty, inequality, and discrimination hold the potential to enhance mental health directly and indirectly by strengthening social cohesion [18]. Such coordinated efforts across governmental levels can complement local initiatives and services capacity investments, fostering a comprehensive approach to improving youth mental health outcomes.

### Limitations

Our model is tailored to the Brisbane South PHN catchment – a geographically diverse region south of the Brisbane River encompassing urban, suburban, and rural communities – and is informed by local data, evidence, and stakeholder input. As such, regional differences in service capacity, help seeking behaviours, and system structure may alter the dynamics between demand and supply, limiting the direct transferability of our quantitative findings. However, the model’s core architecture that integrates population need, help‐seeking, treatment pathways, and social‐environment feedbacks, can be adapted to other similar mixed urban–regional contexts (e.g. comparable areas in Canada, the UK, and Europe). Such adaptation involves (i) re-parameterising and re-calibrating the model using local data (e.g. demographics, baseline distress and disorder prevalence, service‐capacity metrics, social cohesion trends), and (ii) modifying the services sector of the model to reflect jurisdiction‐specific referral mechanisms and service engagement patterns (for instance, GP gatekeeping versus self‐referral systems, or cultural and digital access preferences). By updating these elements, the framework can identify context‐specific leverage points to guide both service‐capacity planning and upstream social‐cohesion interventions in other settings. Second, our study assumed that the trend in social cohesion, as measured by the Scanlon-Monash Index from national survey data, is representative of the Brisbane South region. However, there may be regional variations in the trend of social cohesion that could impact our results. If the decline in social cohesion in Brisbane South is more pronounced or less severe than the national average, it could affect the scale of the projected impacts on youth mental health outcomes. Nonetheless, the overarching insights – particularly, the critical role investments in social cohesion could play in mitigating the negative effects of constrained service capacity on youth mental health – are likely to remain valid. Differences in the trend may influence the magnitude of these impacts, but are unlikely to change the fundamental relationship identified in this study. We also acknowledge that social cohesion itself is a multidimensional and evolving concept whose meaning and measurement vary across cultural and geographic contexts. While our model uses the Scanlon-Monash Index, which captures five dimensions (belonging, worth, social justice, participation, and acceptance/rejection), different communities may prioritize distinct aspects of social cohesion based on their unique sociocultural characteristics. Finally, economic evaluation, including the investment required to achieve the hypothetical changes modelled, has not been included in this analysis. This prohibits conclusions as to whether investments in services capacity growth and/or social cohesion would represent an efficient allocation of resources. Despite these limitations, our research highlights the significant potential of leveraging social cohesion to address the growing youth mental health challenge. Future studies should consider regional variations, more localized data, and economic analysis to further refine these insights and develop tailored interventions that can effectively enhance youth mental health outcomes in diverse settings.

## Conclusion

The continuing deterioration in youth mental health in Australia and other countries necessitates a multifaceted approach that goes beyond merely expanding mental health services. A societal response is needed. Our study underscores the critical role of both primary and specialized mental health services in improving youth mental health outcomes, while also highlighting the substantial constraints posed by workforce shortages and the dynamic nature of unmet need. Importantly, our findings demonstrate the significant potential of social cohesion to mitigate the negative impacts of these constraints. Enhancing social cohesion within communities, schools, and social networks can provide a protective buffer against mental health issues among young people, thereby reducing the burden on the mental health service system. This dual approach of service capacity expansion and social cohesion enhancement offers a more sustainable and effective strategy for addressing the youth mental health challenge.

## Supporting information

10.1192/j.eurpsy.2025.10053.sm001 Occhipinti et al. supplementary material 1 Occhipinti et al. supplementary material

10.1192/j.eurpsy.2025.10053.sm002 Occhipinti et al. supplementary material 2 Occhipinti et al. supplementary material

## Data Availability

Model input data is cited in this published article. No original data were collected for this study. Model output data are available for non-commercial purposes on request to the corresponding author.

## Supplementary material

The supplementary material for this article can be found at http://doi.org/10.1192/j.eurpsy.2025.10053.
