# Supplementary material for: Beyond capacity limits: can social cohesion offset the impact of service constraints on youth mental health?
Source: Eur Psychiatry. 2025 Jun 27;68(1):e93. doi: 10.1192/j.eurpsy.2025.10053 (PMC12260717; doi:10.1192/j.eurpsy.2025.10053)
Supplement: Occhipinti et al. supplementary material 1 — Occhipinti et al. supplementary material [file S0924933825100539sup001.pdf]

Supplementary file 1: Model description

Beyond capacity limits: Can social cohesion offset the impact of service constraints on youth mental health?

Jo-An Occhipinti, PhD,[a,b,*§] Nicholas Ho, BSc. Hons,[a§] Paul Crosland, MHEcon,[a] Sam Huntley, BA(Psych),[a] Wendy Hawkins, BOccThy,[c] Adam Connell, MPH,[d] Judith Piccone, MClinPsych,[e] Sarah Piper, BA(Psych),[a] Seyed Hossein Hosseini, PhD,[a] Catherine Vacher, PhD,[a] Jordan Frith, BPH,[a] Sophie Morson, MClinPsych,[f] Courtney Milham, BHumanServ,[g] Wendy Burton, MD,[h] Kayla Andrade, BA(Arch),[a] Chloe Gosling, BSc (Psych Crim),[a] Kristen Tran, MEc,[a] Yun Ju C. Song, PhD,[a] Victoria Loblay, PhD,[a] Jo Robinson, PhD,[i,j] Adam Skinner, Ph,[a,^] Ian B. Hickie, MD.[a,^]

Affiliations:

[a] Brain and Mind Centre, Faculty of Medicine and Health, University of Sydney, Australia

[b] Computer Simulation & Advanced Research Technologies, Sydney, Australia.

[c] Metro South Hospital and Health Service, Queensland Health, Brisbane, Australia

[d] Mental Health Alcohol and Other Drugs Strategy and Planning Branch, Queensland Health, Brisbane, Australia

[e] Children's Health Queensland Hospital and Health Service; Brisbane, Australia

[f] Thriving Queensland Kids Partnership/Australian Research Alliance for Children and Youth; Brisbane, Australia

[g] Brisbane South Primary Health Network, Brisbane, Australia

[h] Morningside General Practice Clinic, Brisbane, Australia

[i] Orygen, Melbourne, Australia

[j] Centre for Youth Mental Health, The University of Melbourne, Melbourne, Australia

[§] Joint first authors

^ Joint senior authors

*Corresponding author: Jo-An Occhipinti, Head, Systems Modelling & Simulation, The Brain and Mind Centre, Faculty of Medicine & Health, University of Sydney, Moore College, Level 4, 1 King Street, Newtown, NSW, 4042, Australia; Ph: +61 467 522 766; email: jo-an.occhipinti@sydney.edu.au

Model structure

Overview

This system dynamics model was developed for the Brisbane South Primary Health Network (BSPHN) population catchment and consists of multiple sectors that represents different aspects of the population. Supplementary Figure 1 presents an overview of the model structure showing the causal links between different sectors. Supplementary Figures 2 to 22 present the structure of each sector. Please note that for each stock, an inflow representing the suicide deaths prevented through various interventions is included in the model but have been left out of these figures for visual clarity. For further details on each sector, please contact the corresponding author.

The sectors of the model included are:

- **Population** which models the resident population divided into six ages brackets (0-4-year-olds, 5-11, 12-14, 15-17, 18-24, and 25 and older),
- **Education** which models students enrolled in primary, secondary and post-secondary education, and people with different levels of highest qualifications,
- **Labour force** which models unemployment, underemployment and participation rates,
- **Not in education, employment nor training (NEET)** which models the youth population aged 15-24 years not in education, employment nor training,
- **Homelessness** which models the population experiencing homelessness,
- **Substance misuse** which models the prevalence of 12-month substance misuse disorder and substance misuse closed treatment episodes,
- **Psychological distress / disorder** which models the prevalence of low psychological distress and the prevalence of moderate to very high psychological distress. The population with moderate to very high psychological distress is further dichotomised by whether nor not they meet the criteria for any 12-month psychological disorder,
- **Strengths and difficulties** which models the prevalence of behavioural and emotional difficulties among children aged 0-4-years and 5-11-years as measured by the Strengths and Difficulties Questionnaire (SDQ),
- **Social cohesion** which models the population level of social cohesion according to the Scanlon-Monash Index of Social Cohesion,
- **Suicidal behaviours** which models the rates of suicide attempts and suicide deaths, and
- **Mental health services** which models the mental health services delivered by health professionals.

Data used to calibrate each sector are described in figure captions. For any data extracted at the Statistical Area (SA) level of geographic granularity (such as those from the Australian Bureau of Statistics (ABS)), we concorded these data to Primary Health Network (PHN) level estimates through concordance files supplied by the Australian Department of Health and Aged Care [1].

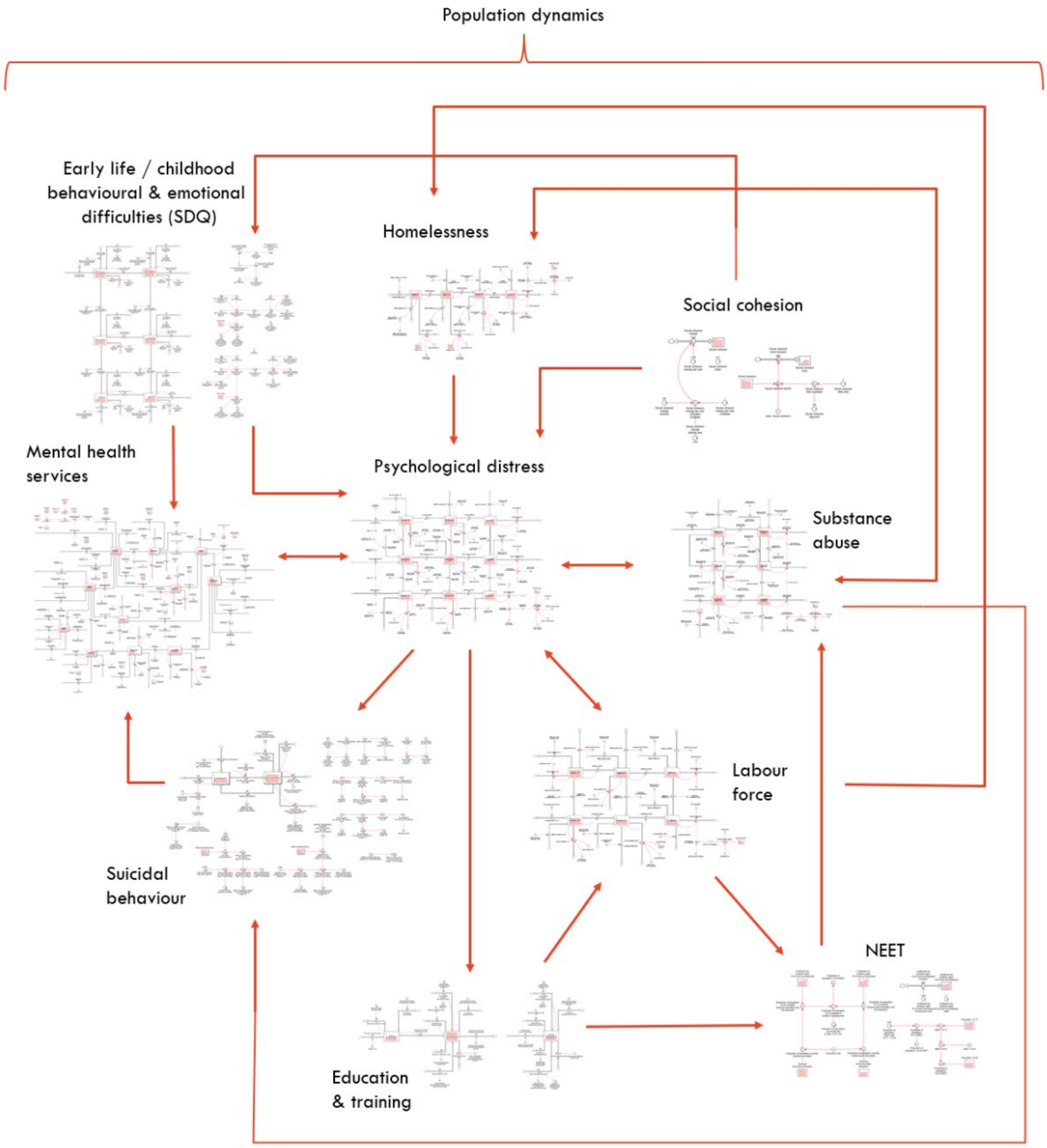

Supplementary Figure 1. Overview of the causal structure of the system dynamics model

Population

Supplementary Figure 2a. Structure of the population sector

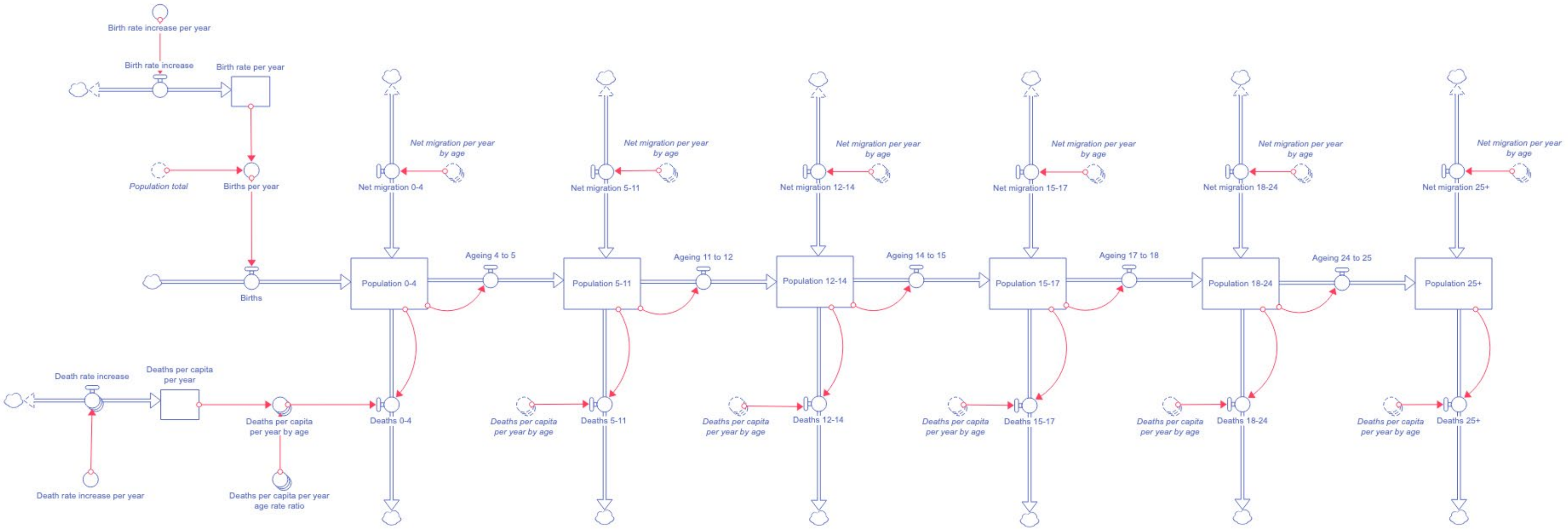

The population sector models the estimated resident population (ERP) of the BSPHN catchment into six age-specific stocks. The stocks correspond to people aged 0-4 years, 5-11 years, 12-14 years, 15-17 years, 18-24 years, and 25 years and older. Each stock has a mortality outflow and a net migration biflow. Births flow into the stock of 0–4-year-olds and people follow an ageing chain flowing from the younger to older age stocks This sector is calibrated with data based on ERP statistics from the Public Health Information Development Unit (PHIDU) at Torrens University Australia [2], and births [3], deaths [4], and migration [5] statistics from the ABS.

Supplementary Figure 2b. Calibration plots from the population sector

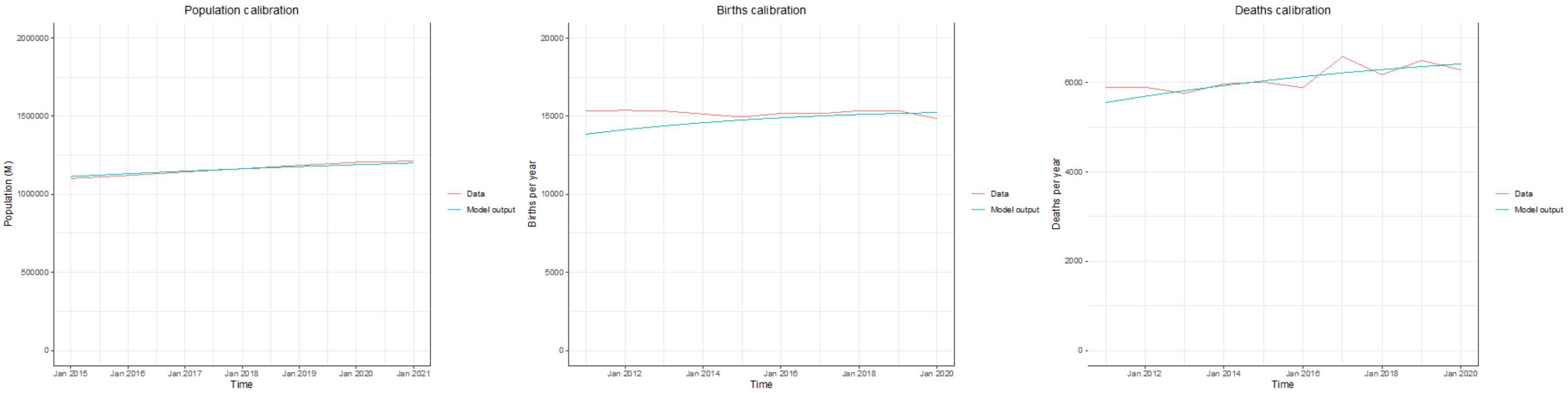

Education (students)

Supplementary Figure 3a. Structure of the education (students) sector

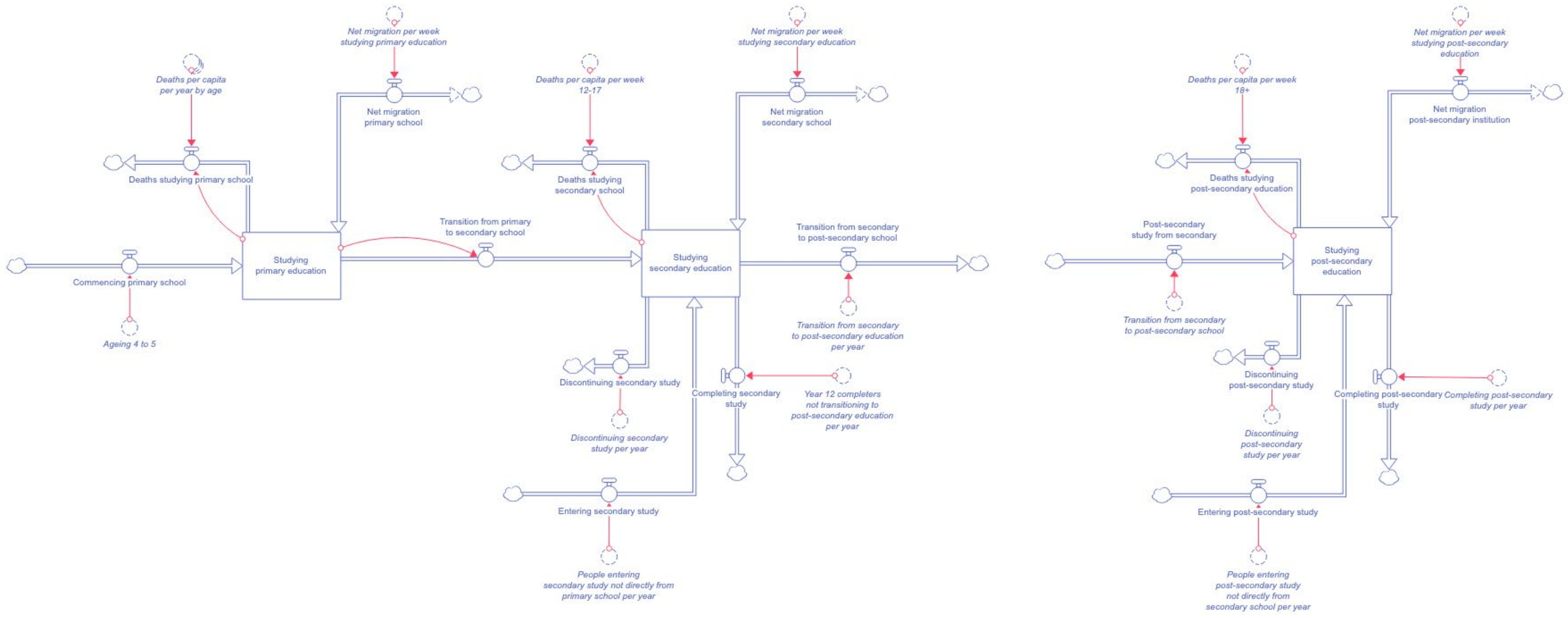

The education (students) sector models students enrolled in education in BSPHN. The stocks correspond to students enrolled in primary education, secondary education and post-secondary education. Each stock has a mortality outflow and a net migration biflow. People flow into the "Studying primary education" stock as they age from 4 to 5 years of age. Graduates of primary education then transition to secondary education, and graduates of secondary education can either transition to post-secondary education or not (e.g. those commencing employment). Students in secondary or post-secondary education may discontinue their studies at rates dependent on the prevalence of psychological distress / disorder [6, 7]. People may enter secondary or post-secondary studies without directly transitioning from primary education or secondary education

respectively (e.g. re-enrolling after discontinuation, people enrolled in both secondary and post-secondary studies). This sector is calibrated using student enrolment [8] and completion [9] data from the Australian Curriculum Assessment and Reporting Authority (ACARA), post-secondary education destinations data from the Queensland Department of Education [10], and education and work statistics from the ABS [11].

Supplementary Figure 3a. Calibration plots from the education (students) sector

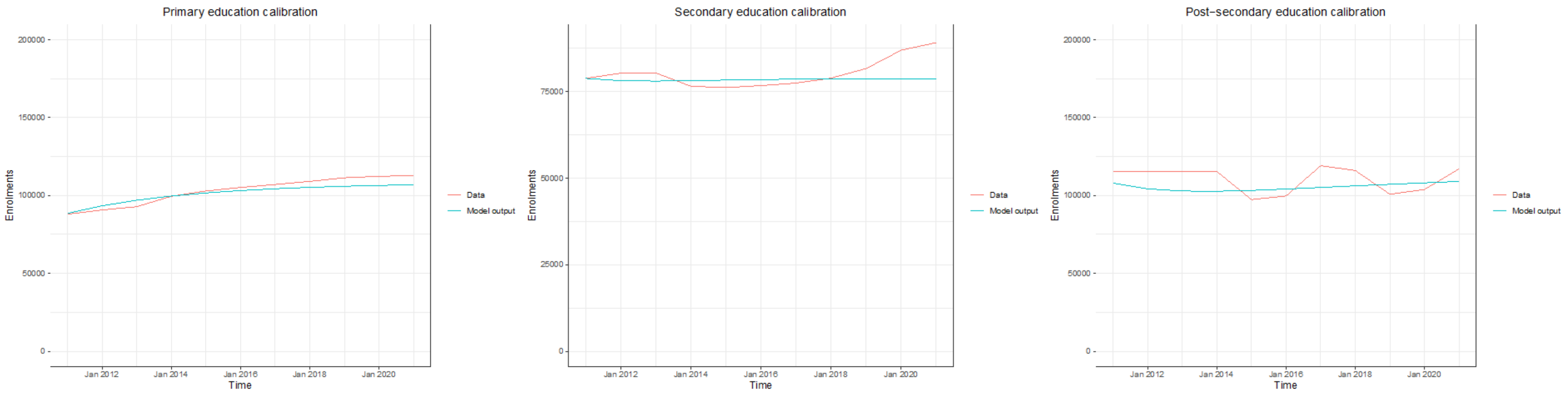

Education (highest level of qualification)

Supplementary Figure 4a. Structure of the education (highest level of qualification) sector

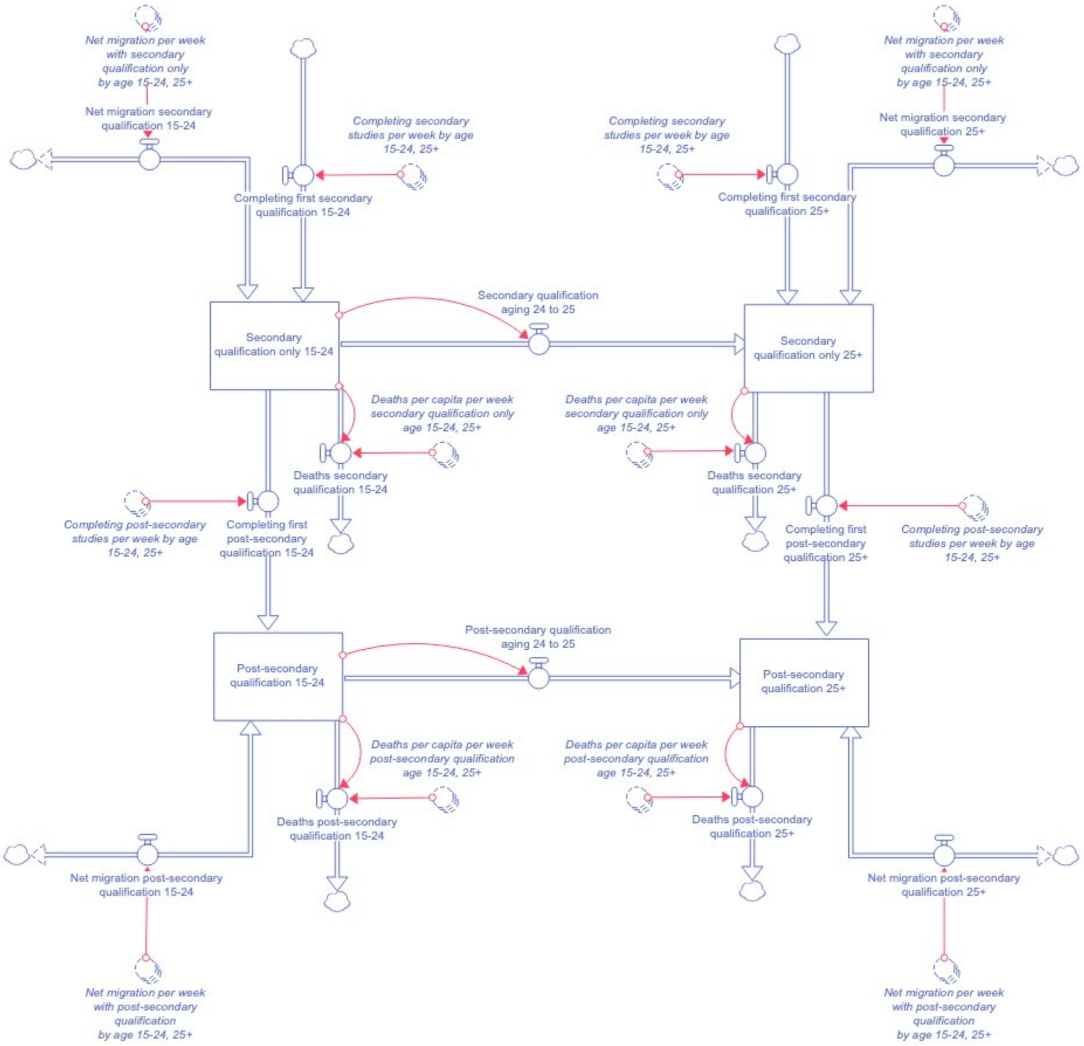

The education (highest level of qualification) sector models holders of different qualifications in BSPHN. The stocks correspond to people aged 15-24 years and 25 years and older, and by their highest level of qualification. More specifically, the stocks correspond to people whose highest level of qualification is Year 12 or equivalent completion, and to people whose highest level of qualification is Certificate III or above. People not in any of these two qualification stocks correspond to people whose highest level of qualification is below Year 12 or equivalent. Each stock has a mortality outflow [12] and a net migration biflow. People who complete secondary education then flow into the "Secondary qualification only" stocks. People who then complete their first post-secondary qualification flow into the "Post-secondary qualification" stocks. People also follow the ageing chain from 15-24 years to 25 years and older. This sector is calibrated using qualifications, education and work statistics from the ABS [11].

Supplementary Figure 4b. Calibration plots from the education (highest level of qualification) sector

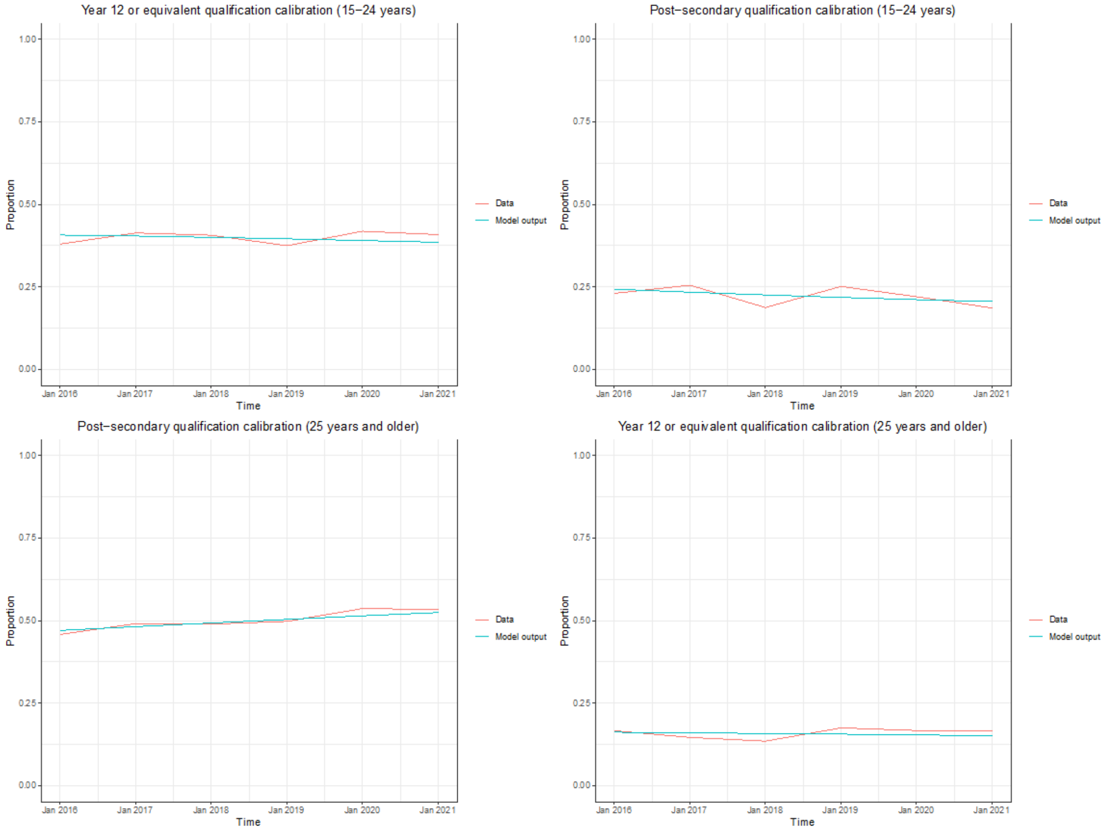

Labour force

Supplementary Figure 5a. Structure of the labour force sector

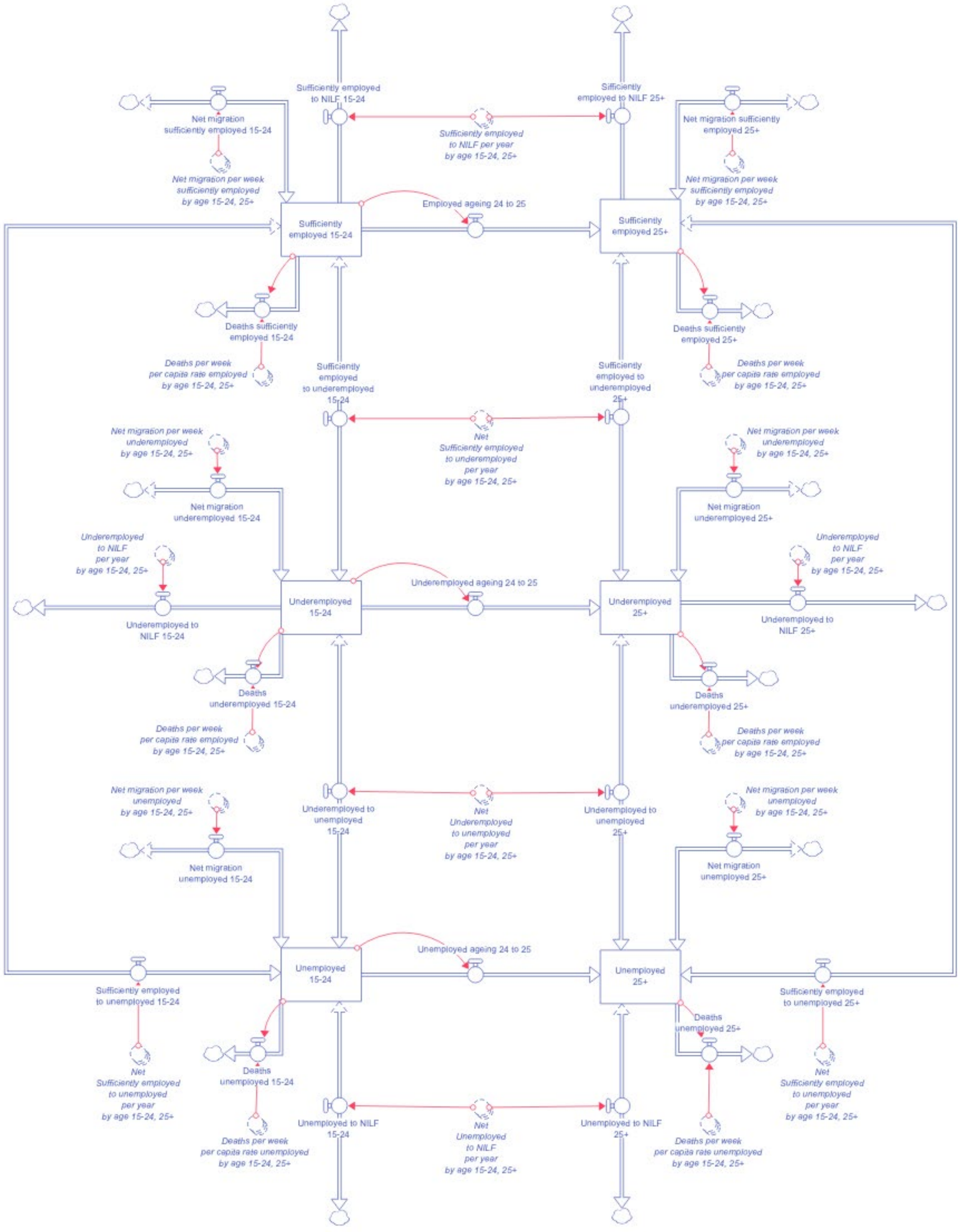

This labour force sector models the employment statuses and flow between these statuses of the BSPHN resident population. The stocks correspond to people aged 15-24 years and 25 years and older, and by their labour force status. More specifically, people can be either sufficiently employed, underemployed or

unemployed. People who are not in either of these stocks are deemed to be not in the labour force (NILF) (e.g. retirees). Each stock has a mortality outflow [13] and a net migration biflow. People can transition between these four labour force statuses with the exception for the NILF population who must transition into unemployment prior to transitioning into employment to reflect people actively looking for work prior to being employed. The rates of transition between employed and unemployed, and between NILF and unemployed are dependent on age and levels of psychological distress / disorder [14, 15] and highest levels of qualifications [11]. The rates of transition between sufficiently employed and underemployed are dependent on age and highest level of qualifications [16]. This sector is calibrated using labour force statistics from the ABS [11, 17, 18].

Supplementary Figure 5b. Calibration plots from the labour force sector

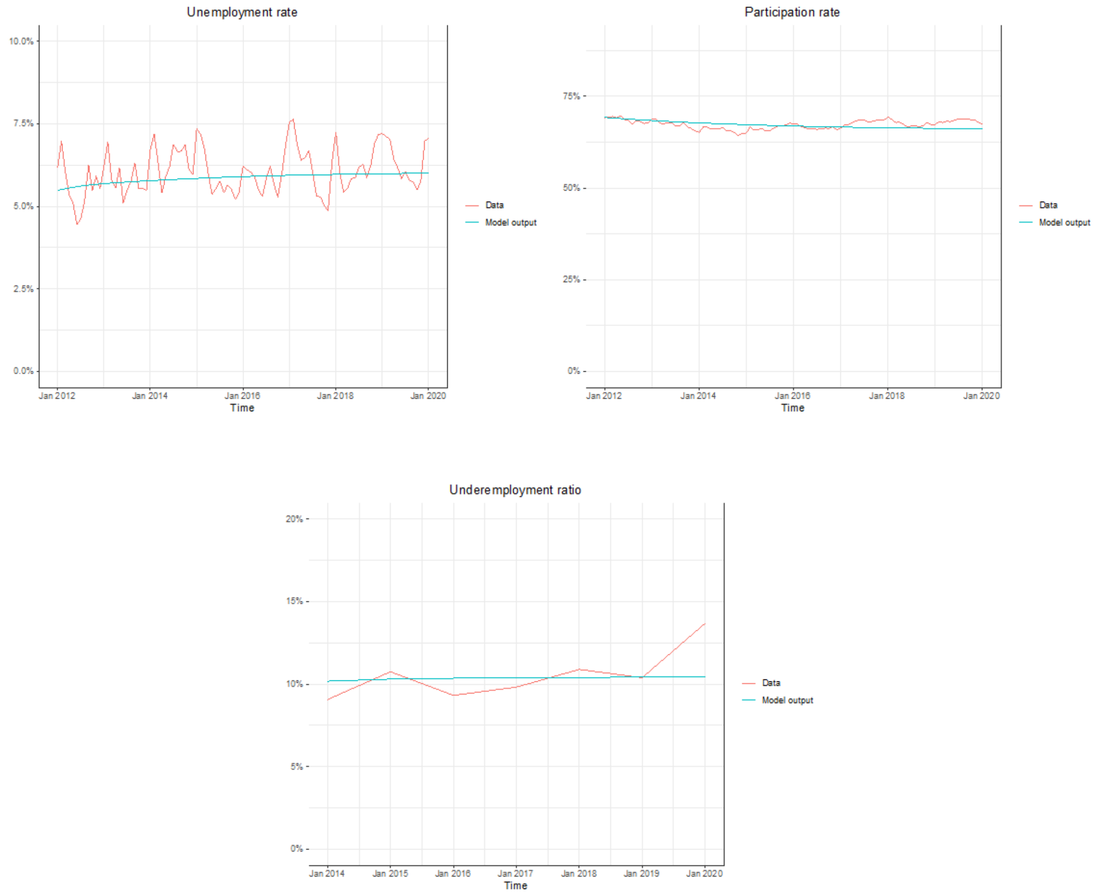

Not in employment nor education (NEET)

Supplementary Figure 6a. Structure of the NEET sector

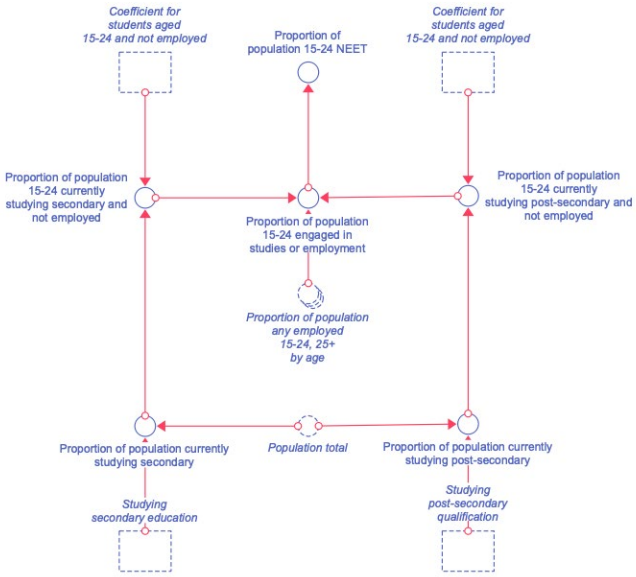

This sector models residents of BSPHN aged 15-24 years who are not in education, employment nor training (NEET). This sector uses model outputs from the labour force and education (students) sectors to calculate the numbers of young people who are NILF and not currently studying. This sector is calibrated using education and work statistics from the ABS [11].

Supplementary Figure 6b. Calibration plot from the NEET sector

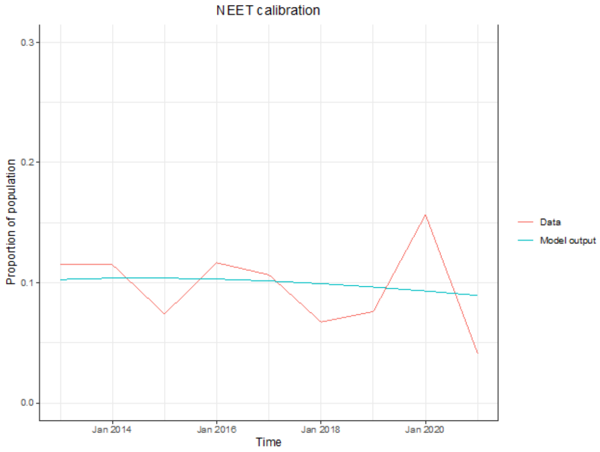

Psychological distress / disorder

Supplementary Figure 7a. Structure of the psychological distress / disorder sector

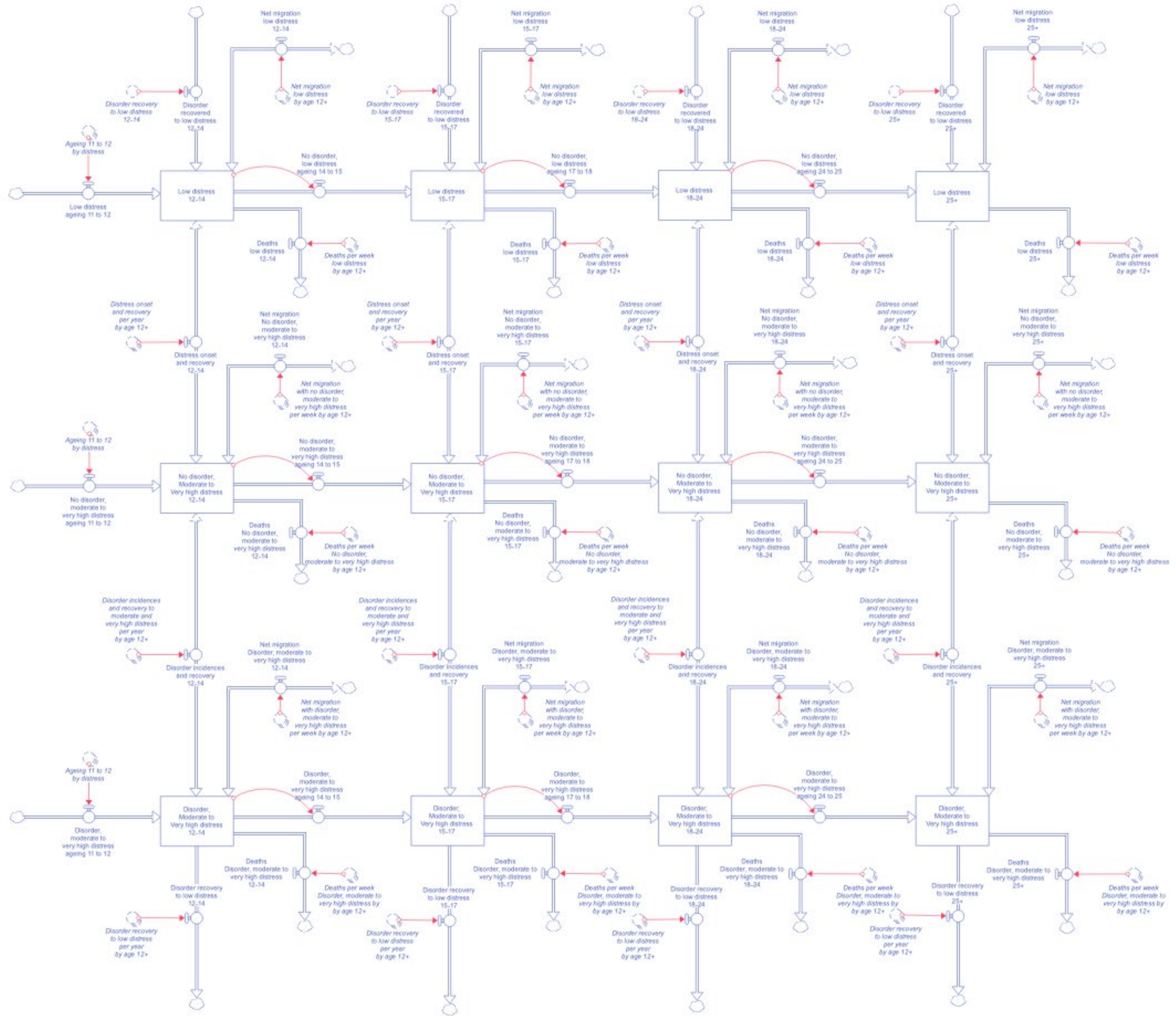

This sector models the prevalence of psychological distress and 12-month psychological disorder in people aged 12-14 years, 15-17 years, 18-24 years, and 25 years and older in BSPHN. These stocks model the population with low psychological distress according to the Kessler Psychological Distress Scale (K10) [19], the population with moderate to very high psychological distress who do not meet the criteria for a 12-month psychological disorder, and the population with moderate to very high psychological distress who meet the criteria for a 12-month psychological disorder. Transition rates between these three levels of psychological distress / disorder are dependent on age, rates of homelessness [20], unemployment [20], underemployment [21, 22], substance misuse [23, 24], engagement and disengagement with the mental health services system and the levels of social cohesion. Each stock has a mortality outflow [25] and a net migration biflow, and the population ages following an ageing chain across each level of psychological distress / disorder. This sector is calibrated using psychological distress prevalence data as measured by the K10 from the ABS' National Health Surveys [15, 26, 27] and the Young Minds Matter Survey [28]. The prevalence estimates for 12-month psychological disorder were modelled from data from the ABS' National Study of Mental Health and Wellbeing [29, 30]. Calibration

for 12-14-year-olds and 15-17-year-olds used data and modelled estimates inferred using the Young Minds Matter Survey [28] and the ABS' National Health Surveys [15, 26, 27].

Supplementary Figure 7b. Calibration plots from the psychological distress / disorder sector

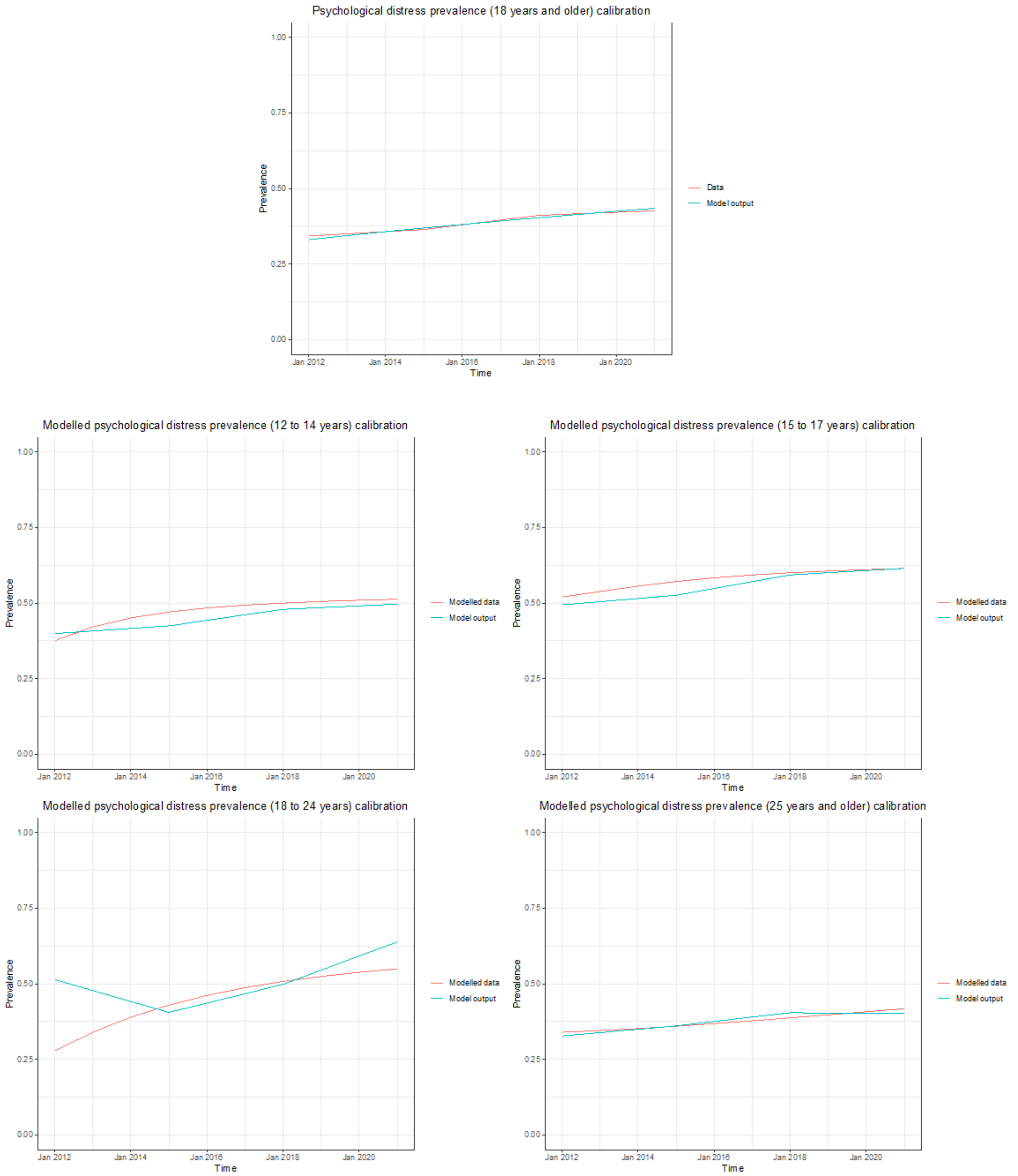

Strengths and Difficulties

Supplementary Figure 8. Structure of the Strengths and Difficulties sector

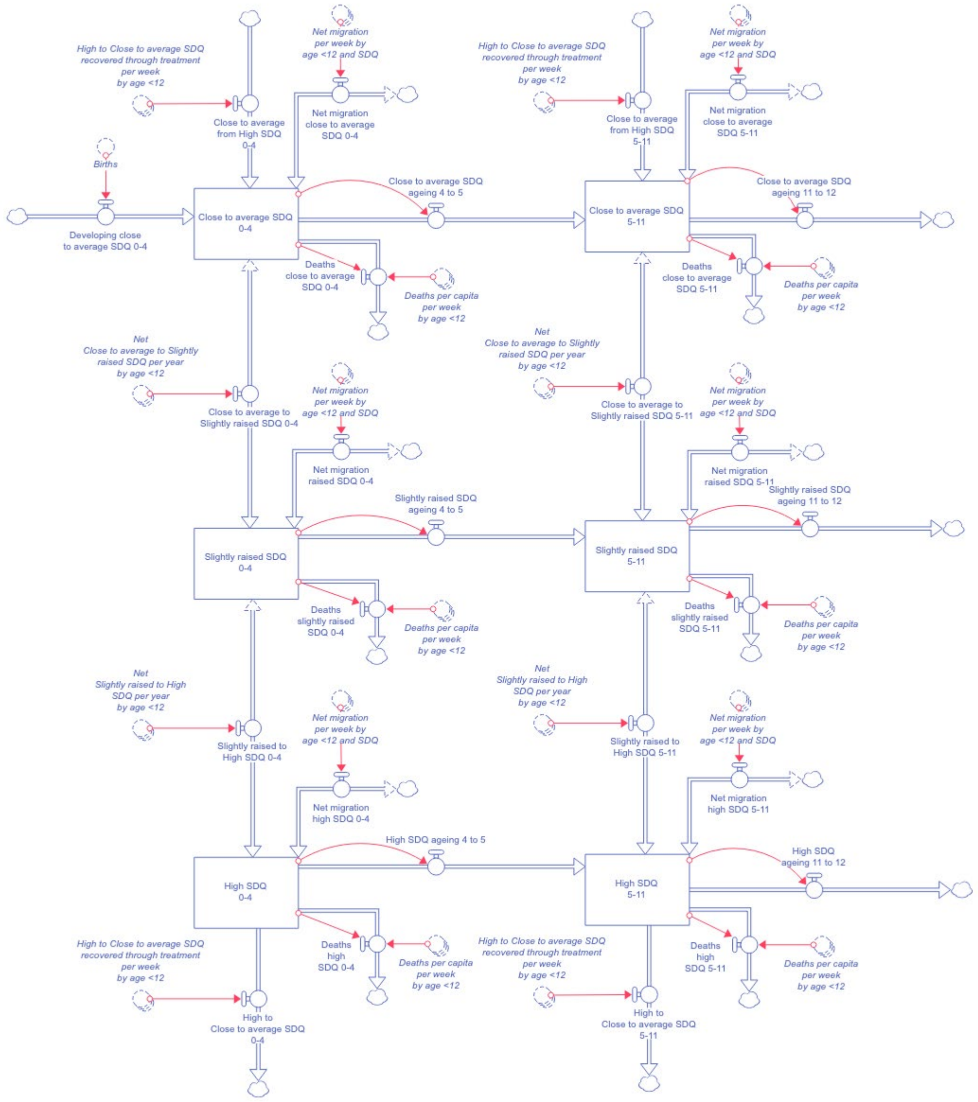

This sector models the prevalence of mental health difficulties in children aged 0-4-years and 5-11-years as measured using the Strengths and Difficulties Questionnaire (SDQ). These stocks model the population with “Close to average”, “Slightly raised” and “High” SDQ scores. People are assumed to be born with close to average levels of SDQ and hence flow into the “Close to average SDQ” stock for 0-4-year-olds. People can flow between close to average and slightly raised levels of SDQ, and between slightly raised to high levels of SDQ with rates dependent on age and rates of engagement and disengagement with the mental health services systems and the levels of social cohesion. Each stock has a mortality outflow and a net migration biflow, and the population ages following an ageing chain across each level of SDQ stocks. This sector is calibrated using SDQ data from the Longitudinal Study of Australian Children (LSAC) [31]. Calibration for 0-4-year-olds used modelled estimates inferred using data for 5-11-year-olds. Please note that, as part of the user agreement between the authors and the LSAC, SDQ data at the PHN level of geographic granularity cannot be shown. As such, calibration plots for the Strengths and Difficulties sector will not be shown here.

Homelessness

Supplementary Figure 9a. Structure of the homelessness sector

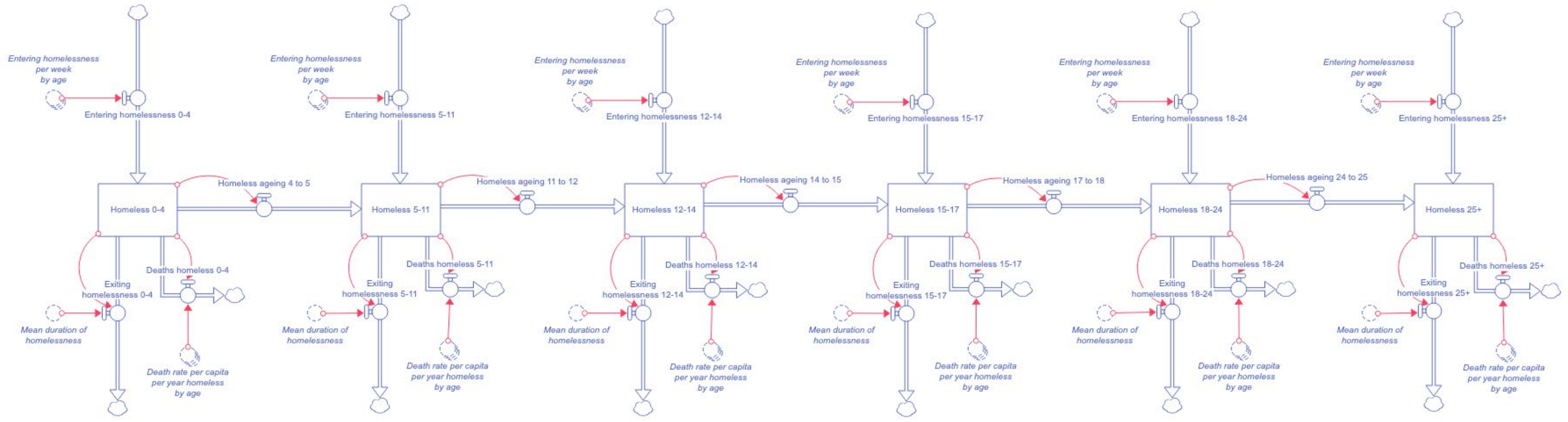

This sector consists of six stocks modelling people experiencing homelessness aged 0-4 years, 5-11 years, 12-14 years, 15-17 years, 18-24 years, and 25 years and older. Each stock has a mortality outflow [32] and a net migration biflow. People aged 15 and older enter homelessness at rates which are dependent on age, levels of psychological distress / disorder, unemployment rates, and substance misuse rates [33]. For people under 15 years of age, rates of entering homelessness are dependent on age. People exit homelessness at rates dependent on the mean duration of homelessness [34]. People age into older stocks following the ageing chain.

This sector was calibrated using homelessness statistics from the ABS [35, 36].

Supplementary Figure 9b. Calibration plot from the homelessness sector

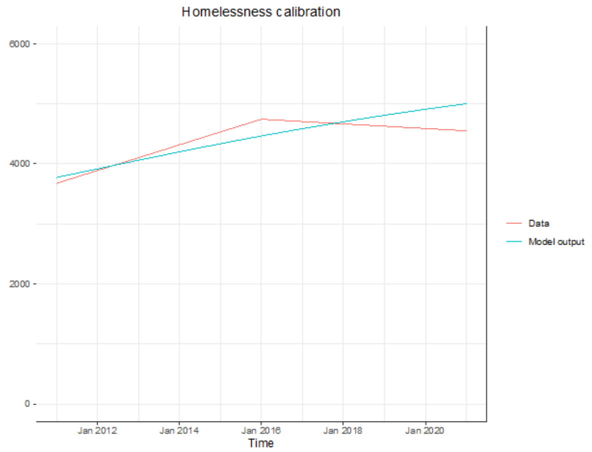

Substance misuse (substance misuse disorder)

Supplementary Figure 10a. Structure of the substance misuse (substance misuse disorder) sector

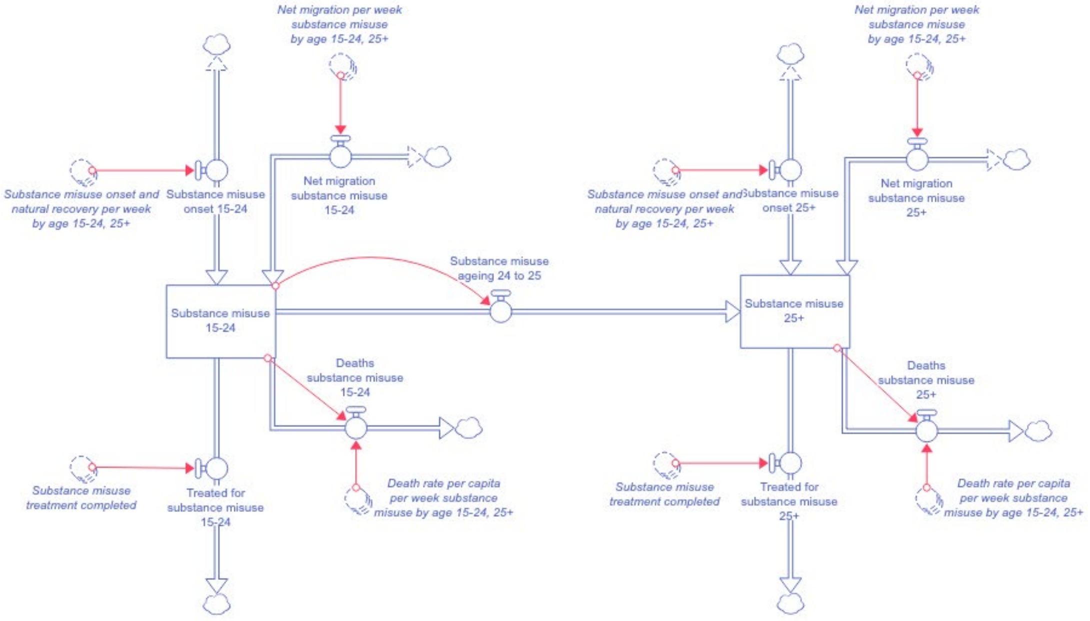

This sector models the prevalence of substance misuse in the BSPHN resident population. The stocks correspond to people aged 15-24 years and 25 years and older who meet the criteria for 12-month substance use disorder. Each stock has a mortality outflow [37] and a net migration biflow, and the population ages following an ageing chain. Each stock has a disorder onset and recovery biflow which reflect the rates of onset of and the non-treatment based recovery from substance misuse disorder. The onset rates are dependent on age, prevalence of psychological distress / disorder [38], homelessness [39, 40] and NEET [30, 41]. Each stock also has a recovery through treatment outflow representing people who recover from substance misuse disorder through treatment with services. This sector is calibrated using national 12-month substance use disorder data from the ABS' National Study of Mental Health and Wellbeing [29, 30].

Supplementary Figure 10b. Calibration plots from the substance misuse (substance misuse disorder) sector

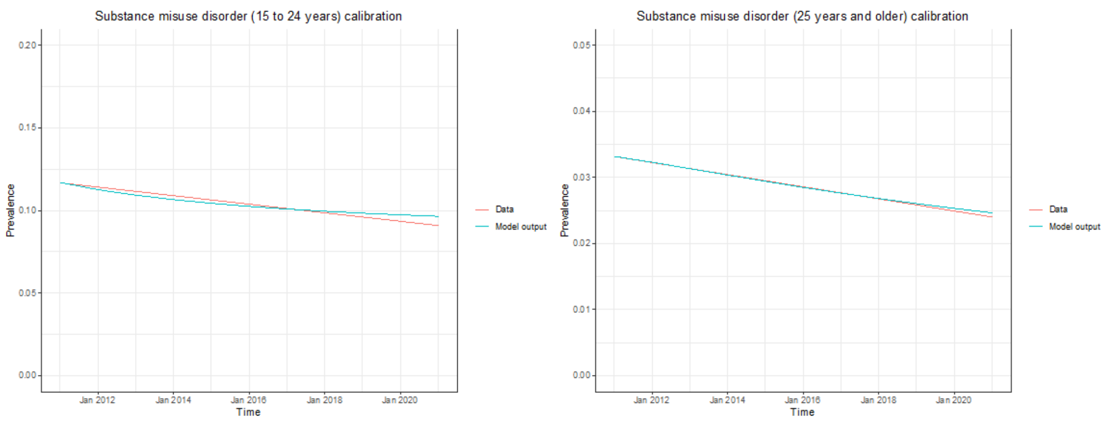

Substance misuse (substance misuse closed treatment episodes)

Supplementary Figure 11a. Structure of the substance misuse (substance misuse closed treatment episodes) sector

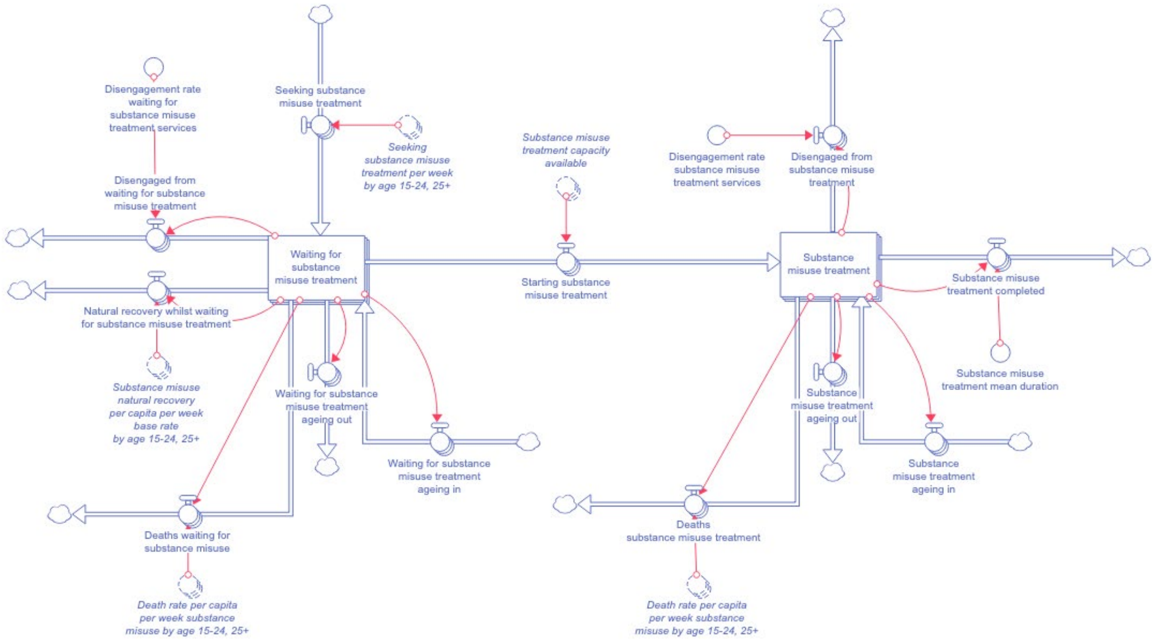

This sector models the flow of people engaging with substance misuse services. People with flow into the substance misuse services waiting stock, representing people on the waitlist for services prior to commencing substance misuse treatment [42]. From the waiting stock, people flow out if they recover without treatment required, if they disengage with services (due to, for example, excessive wait times), through death or, if there are sufficient capacity, through commencing treatment with substance misuse services. From the treatment stock, people flow out if they disengage with services (due to, for example, dissatisfaction with services provided), through death or through the completion of treatment. The remaining flows model ageing, distress / disorder transitions and mortality [37]. This sector was calibrated with substance misuse services data from the AIHW [43, 44].

Supplementary Figure 11b. Calibration plots from the substance misuse (substance misuse closed treatment episodes) sector

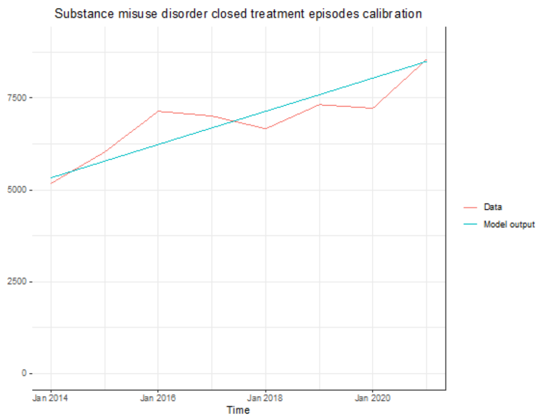

Suicidal behaviours

Supplementary Figure 12a. Structure of the suicidal behaviours sector

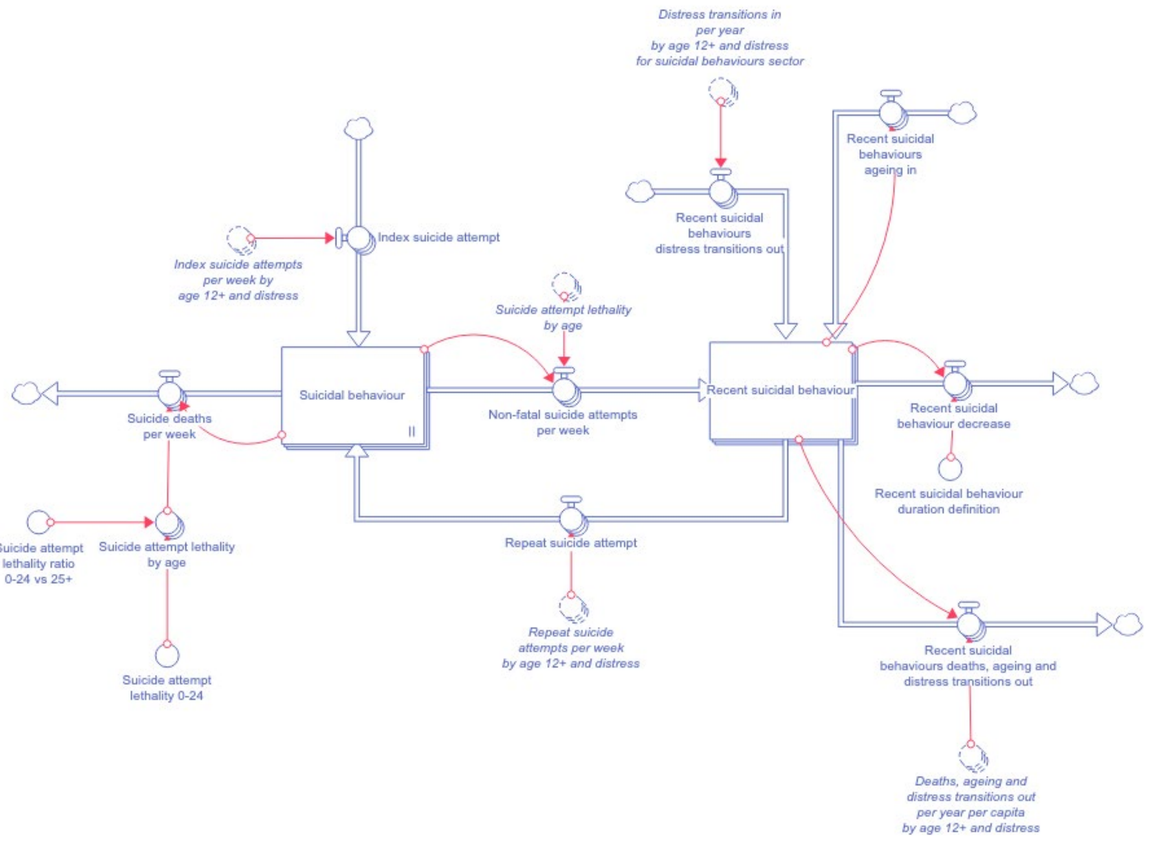

This sector models suicide deaths and attempts in the BSPHN population. The "Suicidal behaviour" stock represents people currently exhibiting suicidal behaviours. People can flow into this stock with an index suicide attempt and people can flow out of this stock depending on whether the attempt was fatal or non-fatal. People who had a non-fatal suicide attempt then flow into the "Recent suicidal behaviour" stock and either remain in this stock for 12 months, representing the duration of which people are at higher risk of exhibiting further suicidal behaviours, or flow back into the "Suicidal behaviours" stock if they have a repeat suicide attempt. The rates of suicide attempts are dependent on age, prevalence of psychological distress / disorder [45] and prevalence of substance misuse disorder [46]. The remaining flows model ageing, distress / disorder transitions and mortality excluding suicide deaths. This sector was calibrated using suicide deaths statistics from the AIHW [47] and intentional self-harm hospitalisations statistics provided by Queensland Health [48].

Please note that we calibrated non-fatal suicide attempts with intentional self-harm hospitalisations data. We acknowledge that these data do not fully capture the number of non-fatal suicide attempts (for example, those events not resulting in hospitalisation) and that these data may not accurately record the intention of the event.

Supplementary Figure 12b. Structure of the suicidal behaviours sector

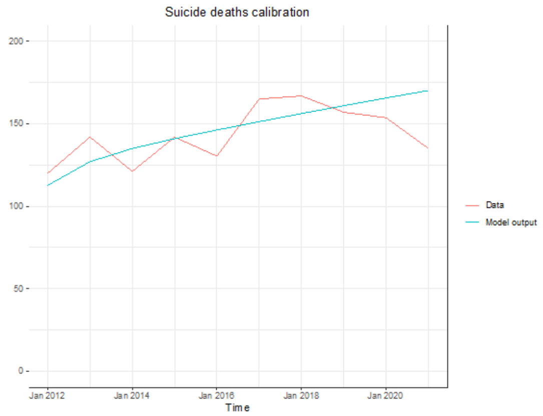
Suicide deaths calibration
200
150
100
50
0
Jan 2012
Jan 2014
Jan 2016
Jan 2018
Jan 2020
Time
Data
Model output

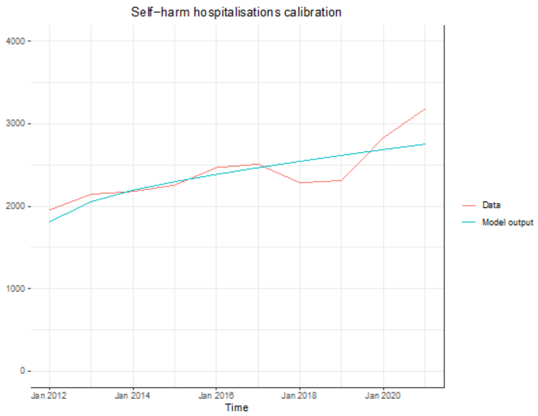
Self−harm hospitalisations calibration
4000
3000
2000
1000
0
Jan 2012
Jan 2014
Jan 2016
Jan 2018
Jan 2020
Time
Data
Model output

Social cohesion

Supplementary Figure 13a. Structure of the social cohesion sector

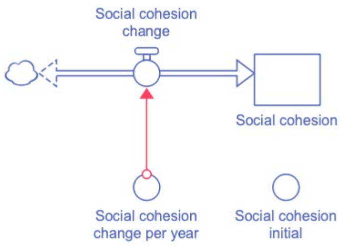

This sector models the level of social cohesion in the population. Social cohesion consists of five domains: belonging, worth, social justice, participation and acceptance and rejection. Social cohesion is a stock and flow structure allowing the level of social cohesion to change at a constant rate per year. This sector was calibrated with the Scanlon-Monash Index of Social Cohesion data from the Scanlon Foundation Research Institute [49].

Supplementary Figure 13b. Calibration plot the social cohesion sector

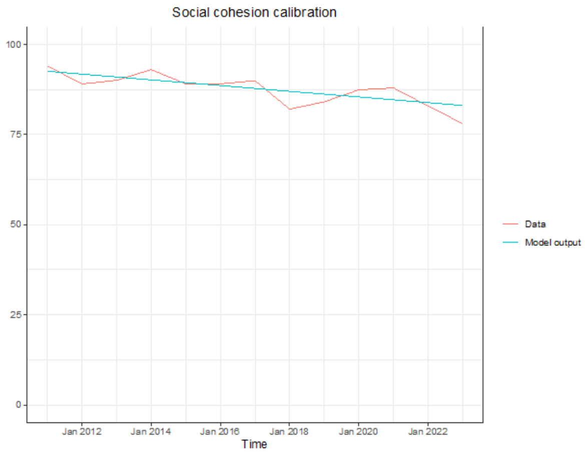

General Practitioner (GP)

Supplementary Figure 14a. Structure of the GP sector

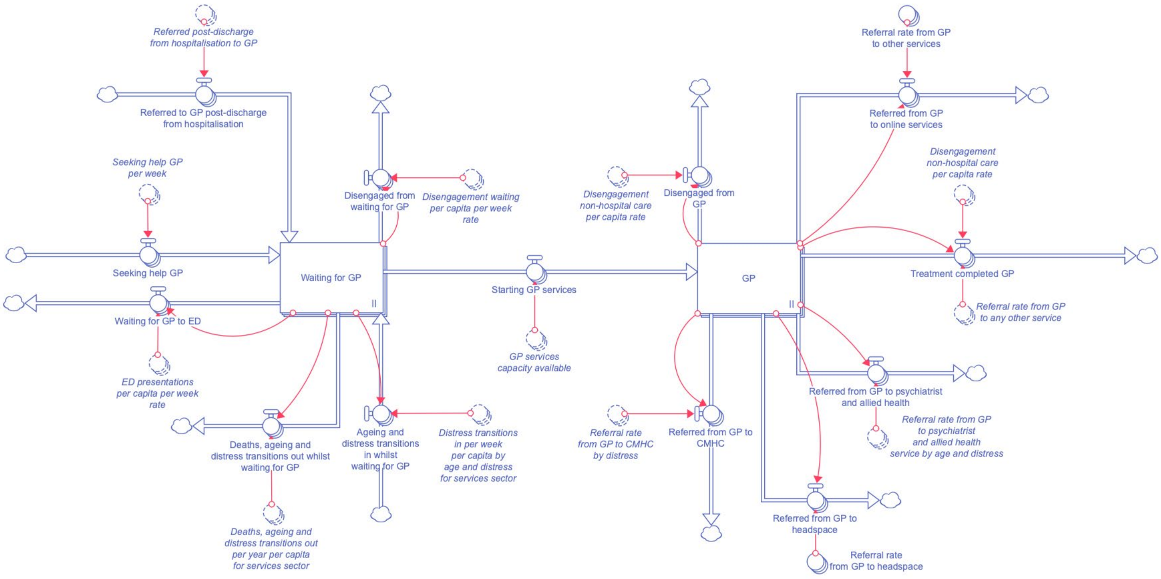

This sector models the flow of people engaging with a GP for their mental health. People flow into waiting stock "Waiting for GP", representing people on the waitlist for GP mental health services. The two inflows into the waiting stock are people in psychological distress who perceive a need for service and commence help-seeking with their GP, and people who have been referred to their GP post-discharge from a mental health related hospitalisation. From the waiting stock, people flow out if they disengage with services (due to, for example, excessive wait times), they present to an emergency department (due to, for example, high levels of distress) or they commence their consult with the GP, if services capacity allows. From the service stock "GP", people flow out if they disengage with services (due to, for example, dissatisfaction with services provided), they are referred to other mental health services (e.g. online mental health services) or if their consult is completed without further referrals. The remaining flows model ageing, distress / disorder transitions and mortality. This sector was calibrated with Medicare-subsidised GP mental health services data from the AIHW [50] and from data provided by BSPHN.

Supplementary Figure 14b. Calibration plot from the GP sector

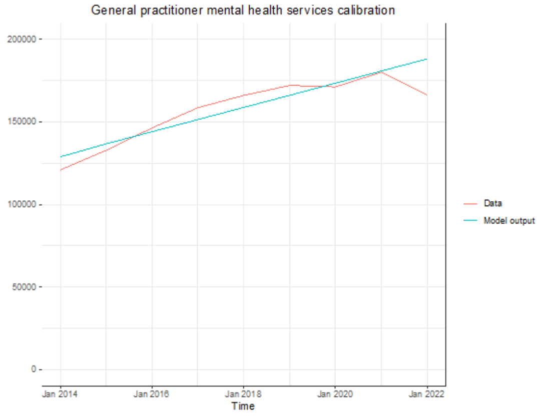

Specialist services

Supplementary Figure 15a. Structure of the specialist services sector

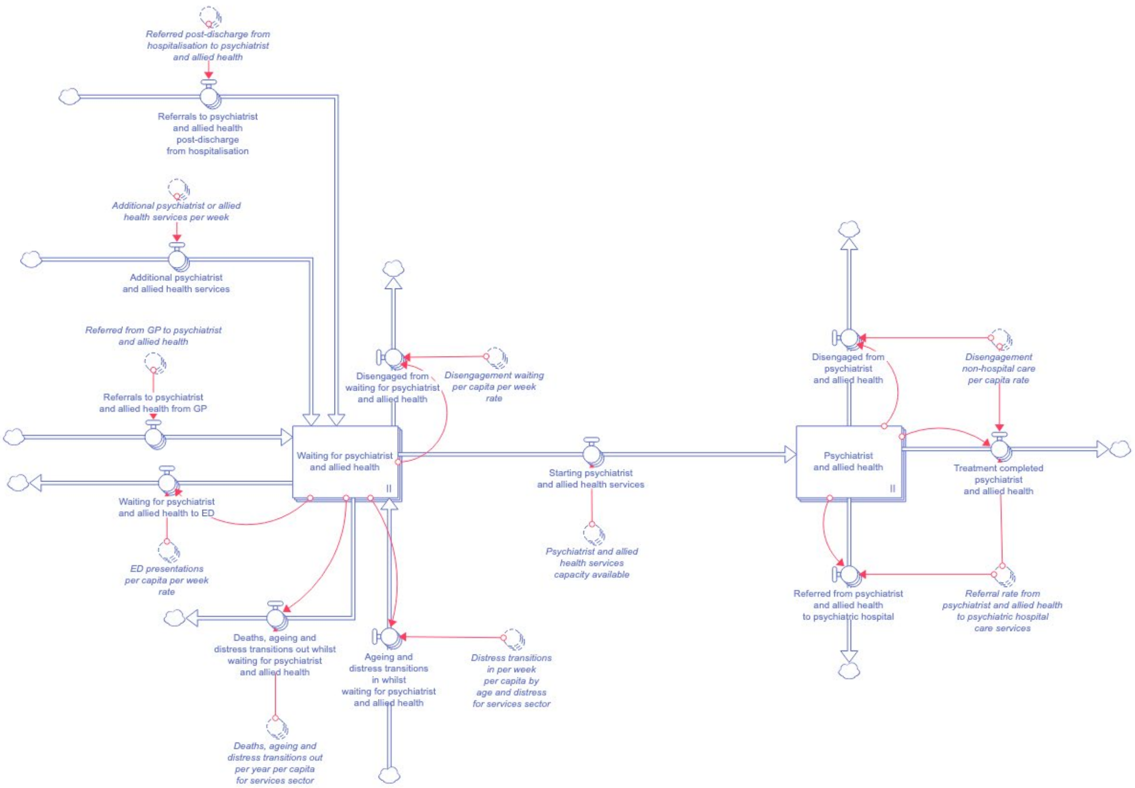

This sector models the flow of people engaging with specialist services (psychiatrists and allied mental health professionals such as psychologists, social worker and occupational therapists). People flow into the waiting stock "Waiting for psychiatrist and allied health", representing people on the waitlist for services. The inflows into the waiting stock are people referred by their GP, people referred post-discharge from a mental health related hospitalisation and people with follow-up appointments. From the waiting stock, people flow out if they disengage with services (due to, for example, excessive wait times), they present to an emergency department (due to, for example, high levels of distress) or they commence their consult with specialised services, if services capacity allows. From the service stock "Psychiatrist and allied health", people flow out if they disengage with services (due to, for example, dissatisfaction with services provided), they are referred to inpatient psychiatric care or if their consult is completed without further referrals. The remaining flows model ageing, distress / disorder transitions and mortality. This sector was calibrated with Medicare-subsidised Psychiatrists and Allied Mental Health mental health services data from the AIHW [50] and from data provided by BSPHN.

Supplementary Figure 15b. Calibration plot from the specialist services sector

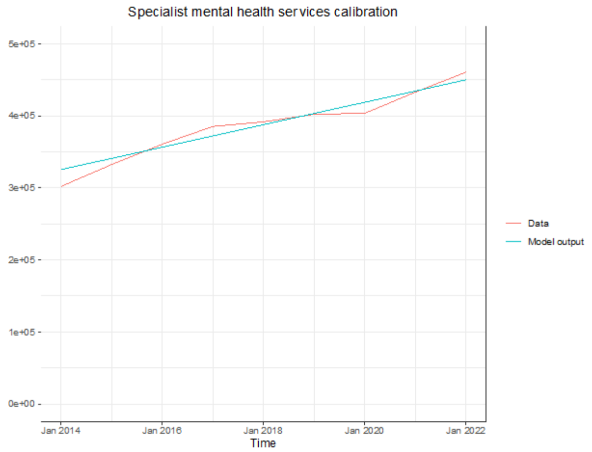

CMHC services

Supplementary Figure 16a. Structure of the CMHC services sector

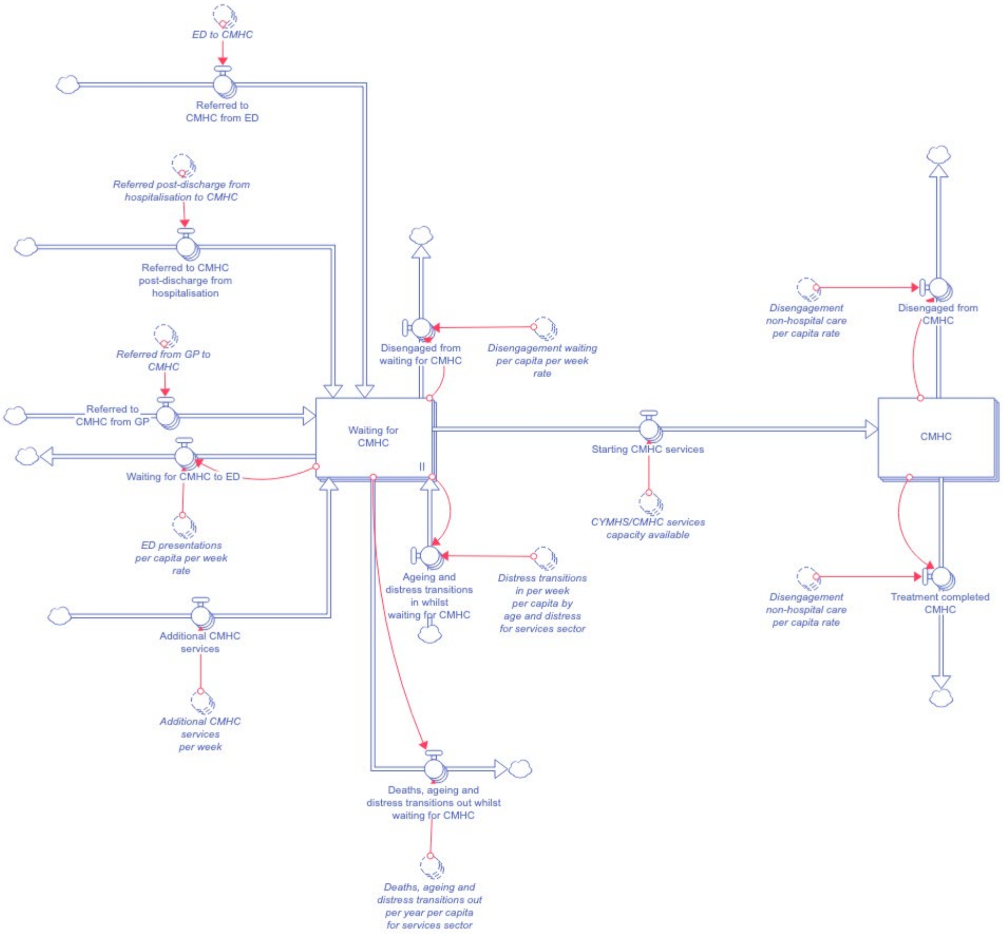

This sub-sector models the flow of people engaging with child and youth mental health services (CYMHS) and community mental health care (CMHC) services. People flow into the waiting stock "Waiting for CMHC", representing people on the waitlist for services. The inflows into the waiting stock are people referred by their GP, people referred post-discharge from a mental health related hospitalisation, people referred post-discharge from a mental health related emergency department presentation and people with follow-up appointments. From the waiting stock, people flow out if they disengage with services (due to, for example, excessive wait times), they present to an emergency department (due to, for example, high levels of distress) or they commence their consult with the CYMHS/CMHC, if services capacity allows. From the service stock "CMHC", people flow out if they disengage with services (due to, for example, dissatisfaction with services provided), or if their consult is completed. The remaining flows model ageing, distress / disorder transitions and mortality. This sector was calibrated with service contacts data from the AIHW [51].

Supplementary Figure 16b. Calibration plot from the CMHC services sector

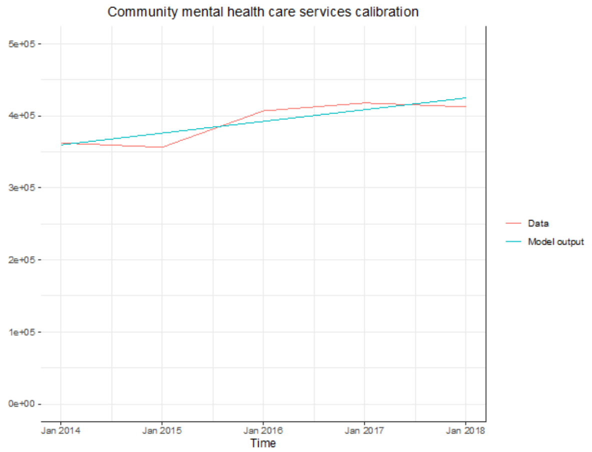

headspace

Supplementary Figure 17a. Structure of the headspace sector

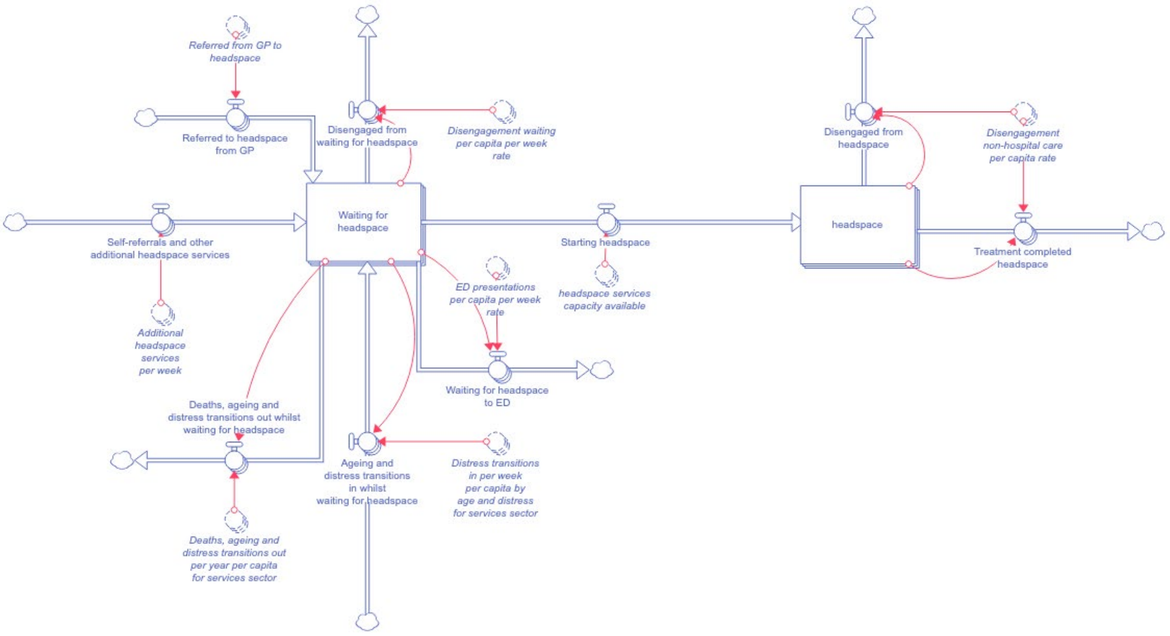

This sector models the flow of young people engaging with youth mental health service provider, headspace. Only people aged 12 and older flow into headspace stocks. People flow into the waiting stock “Waiting for headspace”, representing people on the waitlist for services. The two inflows into the waiting stock are people in psychological distress who perceive a need for service and commence help-seeking with headspace, and people who have been referred by their GP. The first inflow reflects people self-referring to headspace, people referred to headspace by family or friends and people with follow-up appointments. From the waiting stock, people flow out if they disengage with services (due to, for example, excessive wait times), they present to an emergency department (due to, for example, high levels of distress) or they commence their consult with headspace, if services capacity allows. From the service stock “headspace”, people flow out if they disengage with services (due to, for example, dissatisfaction with services provided), or if their consult is completed without further referrals. The remaining flows model ageing, distress / disorder transitions and mortality. This sector was calibrated with occasions of service data provided by BSPHN.

Supplementary Figure 17b. Calibration plot from the headspace sector

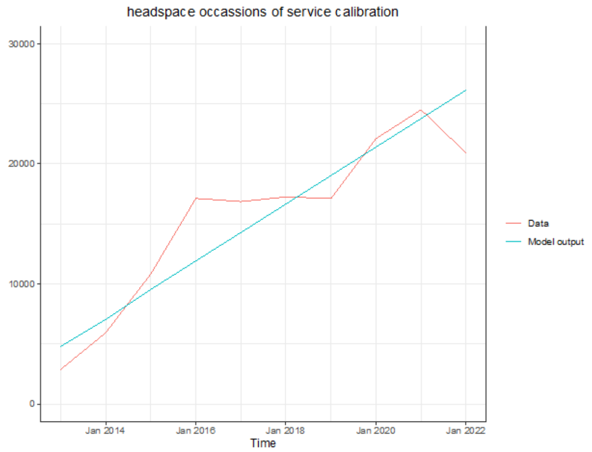

Online mental health services

Supplementary Figure 18. Structure of the online mental health services sector

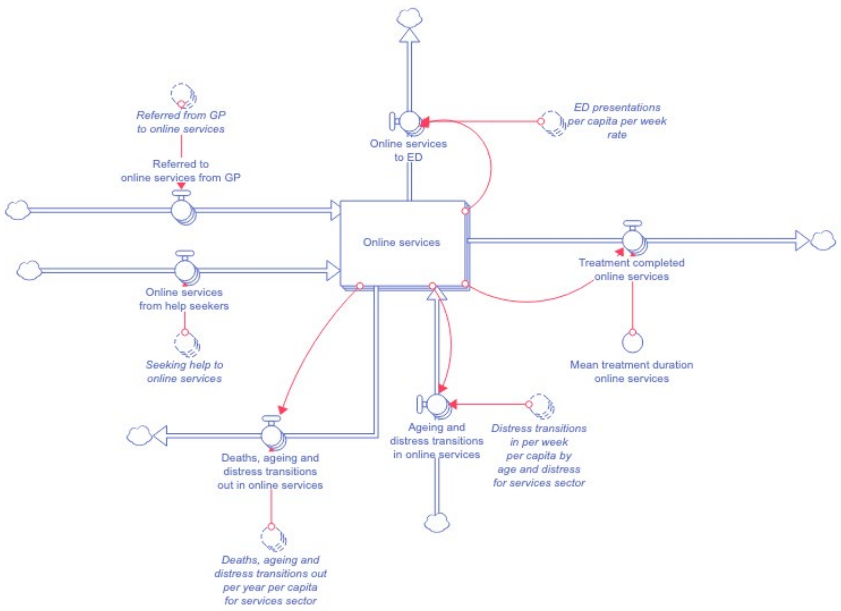

This sector models the flow of people engaging online mental health services. The two inflows into the stock are people in psychological distress who perceive a need for service and commence help-seeking with online services, and people who have been referred by their GP. People flow out when their online course of treatment is completed. The remaining flows model ageing, distress / disorder transitions and mortality.

Mental health related emergency department (ED) presentations

Supplementary Figure 19a. Structure of the mental health related ED presentations sector

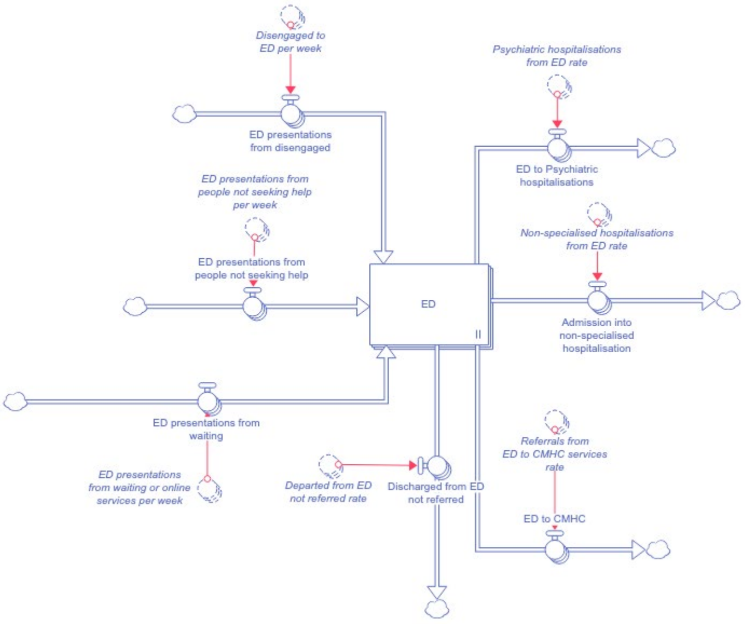

This sector models people presenting to the ED for mental health related presentations. People that flow into the stock are people who are distressed and are currently help-seeking with other services (e.g., whilst waiting for a consult with a psychologist), are not currently help-seeking (e.g., people whose family or friends take them to ED) and people who are disengaged from services. From the stock, people flow out if they are admitted into either psychiatric admitted care or non-specialised admitted care, discharged and referred to CMHC, or discharged without further referrals. This sector was calibrated with mental health related ED presentations data provided by Queensland Health [48].

Supplementary Figure 19b. Calibration plot from the mental health related ED presentations sector

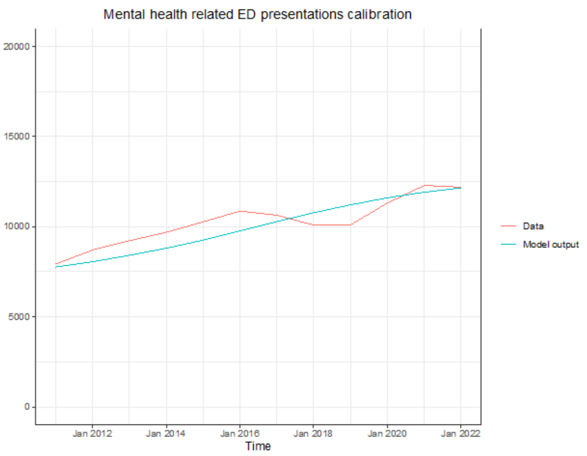

Psychiatric admitted care

Supplementary Figure 20a. Structure of the psychiatric admitted care sector

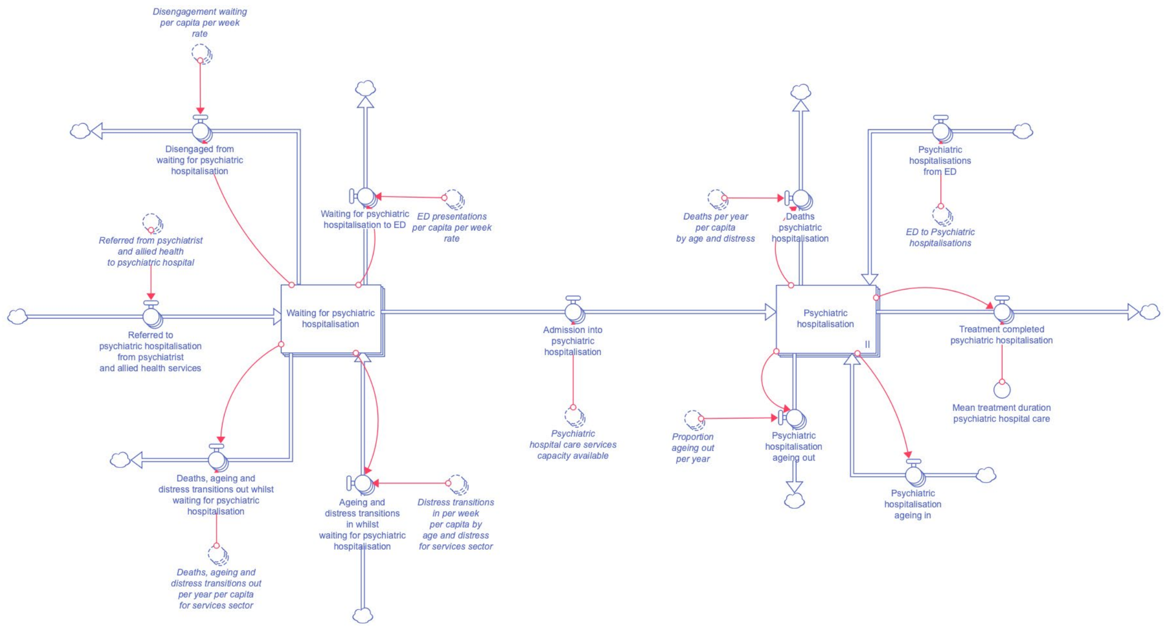

This sector models the flow of people into psychiatric admitted care. People flow into the waiting stock "Waiting for psychiatric hospitalisation", representing people on the waitlist for services. The inflow into the waiting stock are people referred by their specialist. From the waiting stock, people flow out if they disengage with services (due to, for example, excessive wait times), they present to an emergency department (due to, for example, high levels of distress) or they commence their psychiatric admitted care, if services capacity allows. People can directly flow into the service stock if the admission is from the ED. From the service stock "Psychiatric hospitalisation", people flow out once they are discharged. The remaining flows model ageing, distress / disorder transitions and mortality. This sector was calibrated with episodes of admitted care data provided by Queensland Health [48].

Supplementary Figure 20b. Calibration from the psychiatric admitted care sector

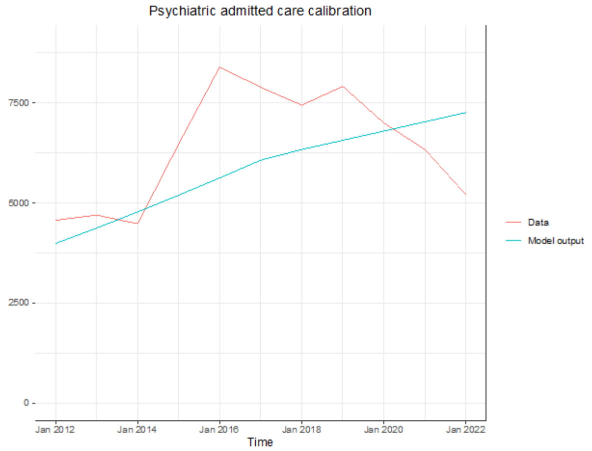

Non-specialised admitted care

Supplementary Figure 21a. Structure of the non-specialised admitted care sector

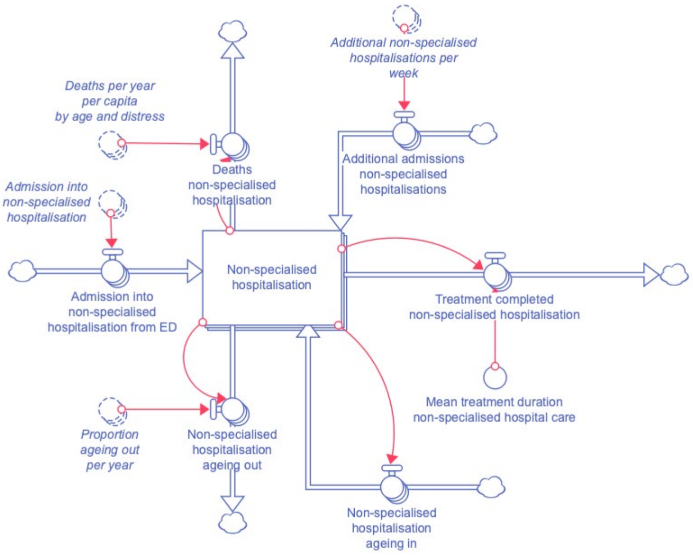

This sector models the flow of people into non-specialised, mental health related admitted care. People that flow into the stock are people who are admitted from ED and any other additional admissions. From the service stock, people flow out once they are discharged. The remaining flows model ageing, distress / disorder transitions and mortality. This sector was calibrated with episodes of admitted care data provided by Queensland Health [48].

Supplementary Figure 21b. Calibration plot from the non-specialised admitted care sector

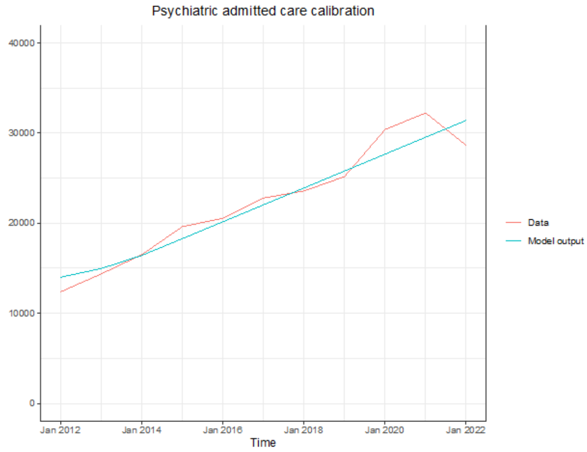

Help-seeking and disengaged

Supplementary Figure 22. Structure of seeking-help and disengaged

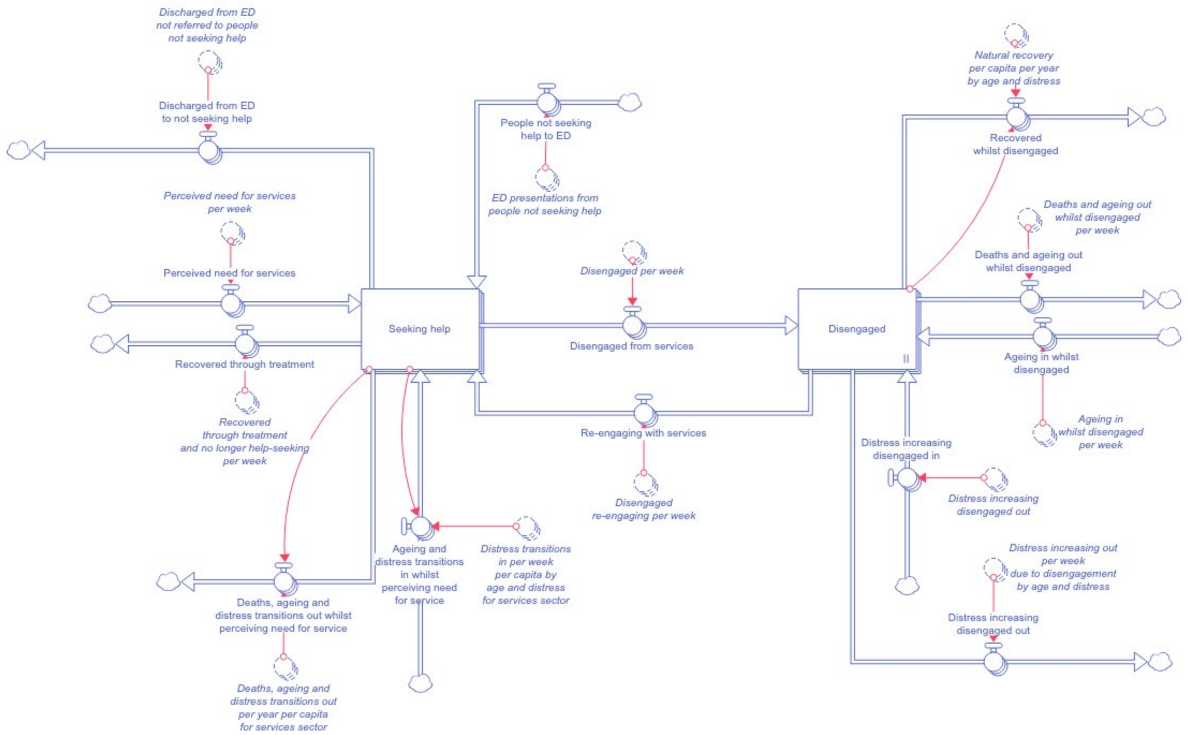

This models the flow of people in distress who perceive a need for service and are either seeking help or are disengaged from services. People in distress may develop a perceive need for service and flow into the ”Seeking help” stock. The other inflows are people who present to ED without a perceived need for service (e.g., people whose family or friends take them to ED) and people who were disengaged then re-engaging with services. People who present to ED without a perceived need for service will flow out of the “Seeking help” stock once they are discharged. The flow from “Seeking help” to “Disengaged” models people who disengage from services whilst waiting for services or from their treatment. Whilst disengaged, people develop psychological disorders at a higher rate than the total population’s per-capita rate. The remaining flows model ageing, distress / disorder transitions and mortality. This sector was calibrated with perceived need for service data from ABS’ National Survey of Mental Health and Wellbeing 2007 [20] and the AIHW’s Mental health perfomance indicators 2021 [52].

Services capacity growth rate

To reflect a slowing growth rate in services capacity in recent years compared to the longer term historic trend, multipliers have been applied to annual growth rates for forward projections (i.e., from January 2025). These mulipliers can be modified on the user interface.

Service	Growth rate
General practitioner mental health services	Based on Medicare-subsidised services data published by AIHW for the period 2014 to 2022 [50], the maximum number of GP mental health services that can be delivered per week has been increasing at an annual rate of 140 additional services per week. This assumes that services were operating at maximum capacity over this period.<br><br>The default value for the future growth rate multiplier (0.68) was derived from Medicare-subsidised services data published by AIHW for the period 2017-2020 (pre-pandemic) [50] and represents a decrease in the annual growth rate in GP services capacity of 32% from January 2025.
Specialist mental health services	Based on Medicare-subsidised services data published by AIHW for the period 2014 to 2022 [50], the maximum number of Psychiatry and allied mental health services that can be delivered per week has been increasing at an annual rate of 298 additional services per week. This assumes that services were operating at maximum capacity over this period.<br><br>The default value for the future growth rate multiplier (0.97) was derived from Medicare-subsidised services data published by AIHW for the period 2017-2020 [50] and represents a decrease in the annual growth rate from January 2025 onwards.
Child and youth mental health services	Based on CMHC Services published by AIHW for the period 2013 to 2018 [51], the maximum number of child and youth mental health services that can be delivered per week has been increasing at an annual rate of 89 additional services per week. This assumes that services were operating at maximum capacity over this period.<br><br>The default value for the future growth rate multiplier (0.41) was derived from CMHC Services data published by AIHW [51] for the period 2016-2018 and represents a decrease in the annual growth rate from January 2025 onwards.
CMHC services	Based on CMHC Services published by AIHW for the period 2013 to 2018 [51], the maximum number of CMHC services that can be delivered per week has been increasing at an annual rate of 222 additional services per week. This assumes that services were operating at maximum capacity over this period.

	The default value for the future growth rate multiplier (0.07) was derived from CMHC Services data published by AIHW [51] for the period 2016-2018 and represents a decrease in the annual growth rate from January 2025 onwards.
headspace	Based on headspace occasions of service data provided by BSPHN for the period 2013 to 2022, the maximum number of headspace services that can be delivered per week has been increasing at an annual rate of 45 additional services per week. This assumes that services were operating at maximum capacity over this period.<br><br>The default value for the future growth rate multiplier (0.58) was derived from headspace occasions of service data provided by BSPHN for the period 2017-2022 and represents a decrease in the annual growth rate from January 2025 onwards.
Psychiatric admitted care	Based on episodes of admitted patient care provided by Queensland Health [48] for the period 2011 to 2022, the maximum number of episodes of admitted patient care that can be delivered per week has been increasing at an annual rate of 4 additional episodes per week. This assumes that services were operating at maximum capacity over this period.<br><br>The default value for the future growth rate multiplier (0.08) was derived from episodes of admitted patient care provided by Queensland Health [48] for the period 2017-2019 and represents a decrease in the annual growth rate from January 2025 onwards.
Substance misuse treatment services	Based on alcohol and other drug closed treatment episodes published by AIHW [43] for the period 2013-14 to 2020-2021, the maximum number of closed treatment episodes that can be delivered per week has been increasing at an annual rate of 9 additional episodes per week. This assumes that services were operating at maximum capacity over this period.<br><br>The default value for the future growth rate multiplier (0.77) was derived from alcohol and other drug closed treatment episodes published by AIHW [43] for the period 2017-18 to 2019-20 and represents a decrease in the annual growth rate from January 2025 onwards.

Numerical inputs

Variable Name	Stratification / Value		Notes
**Population**			
Population initial by age	0–4 years	73403.32	Estimated with constrained optimisation using data based on ERP statistics from PHIDU [2], and births [3], deaths [4], and migration [5] statistics from the ABS.
	5–11 years	90745.96	
	12-14 years	35261.66	
	15-17 years	42747.12	
	18-24 years	112203	
	25 years and older	662238.1	
Birth rate per year initial	0.013619180		Estimated with constrained optimisation using data based on ERP statistics from PHIDU [2], and births [3], deaths [4], and migration [5] statistics from the ABS.
Birth rate increase per year	-0.0000904897		Estimated with constrained optimisation using data based on ERP statistics from PHIDU [2], and births [3], deaths [4], and migration [5] statistics from the ABS.
Death rate per year initial	0.005606927		Estimated with constrained optimisation using data based on ERP statistics from PHIDU [2], and births [3], deaths [4], and migration [5] statistics from the ABS.
Death rate increase per year	-0.0000030102		Estimated with constrained optimisation using data based on ERP statistics from PHIDU [2], and births [3], deaths [4], and migration [5] statistics from the ABS.
Deaths per capita per year age rate ratio	0–4 years	0.15787930	[53]
	5–11 years	0.01387892	
	12-14 years	0.01618564	
	15-17 years	0.05522237	
	18-24 years	0.07360028	
	25 years and older	1.45942	
Arrivals per year by age initial	0–4 years	21364.22826	Estimated with constrained optimisation using data based on ERP statistics from PHIDU [2], and births [3], deaths [4], and migration [5] statistics from the ABS.
	5–11 years	21421.73749	
	12-14 years	3302.189961	
	15-17 years	7339.889174	
	18-24 years	45644.2283	
	25 years and older	61372.53703	
Arrivals increase per year by age	0–4 years	0	Estimated with constrained optimisation using data based on ERP statistics from PHIDU [2], and births [3], deaths [4], and migration [5] statistics from the ABS.
	5–11 years	31.98119806	
	12-14 years	34.44288621	
	15-17 years	0.66163482	
	18-24 years	0	
	25 years and older	16.90088416	
Per capita departure rate by age initial	0–4 years	0.264754914	Estimated with constrained optimisation using data based on ERP statistics from PHIDU [2], and births [3], deaths [4], and migration [5] statistics from the ABS.
	5–11 years	0.188861708	
	12-14 years	0.076197284	

	15-17 years	0.158536797	
	18-24 years	0.360280937	
	25 years and older	0.079111422	
Per capita departure rate by age increase per year	0–4 years	0.000115989	Estimated with constrained optimisation using data based on ERP statistics from PHIDU [2], and births [3], deaths [4], and migration [5] statistics from the ABS.
	5–11 years	0.000067905	
	12-14 years	0	
	15-17 years	0	
	18-24 years	0.000457962	
	25 years and older	0	
**Education - Students**			
Studying post-secondary education initial	107910.9861		Estimated with constrained optimisation using student enrolment [54] and completion [55] data from ACARA, post-secondary education destinations data from the Queensland Department of Education [10], and education and work statistics from the ABS [11].
Proportion of population not currently studying entering secondary education per year	0.000001319		Estimated with constrained optimisation using student enrolment [54] and completion [55] data from ACARA, post-secondary education destinations data from the Queensland Department of Education [10], and education and work statistics from the ABS [11].
Proportion of population not currently studying entering post-secondary education per year	0.04781599		Estimated with constrained optimisation using student enrolment [54] and completion [55] data from ACARA, post-secondary education destinations data from the Queensland Department of Education [10], and education and work statistics from the ABS [11].
Discontinuing secondary study base rate	0.0394836834639		Estimated with constrained optimisation using student enrolment [54] and completion [55] data from ACARA, post-secondary education destinations data from the Queensland Department of Education [10], and education and work statistics from the ABS [11].
Discontinuing post-secondary study base rate	0.083776167		Estimated with constrained optimisation using student enrolment [54] and completion [55] data from ACARA, post-secondary education destinations data from the Queensland Department of Education [10], and education and work statistics from the ABS [11].
Completing secondary study rate	0.144451109414		Estimated with constrained optimisation using student enrolment [54] and completion [55] data from ACARA, post-secondary education destinations data from the Queensland Department of Education [10], and education and work statistics from the ABS [11].
Completing post-secondary study rate	0.427533386		Estimated with constrained optimisation using student enrolment [54] and completion [55] data from ACARA, post-secondary education destinations data from the Queensland Department of Education [10], and education and work statistics from the ABS [11].
Proportion of secondary study completers transitioning to post-secondary education	0.4796904		[10]
Proportion of population 5-11 studying primary education ratio: rest of Australia vs QLD	0.969		[56]
Proportion of population 12-17 studying secondary education ratio: rest of Australia vs QLD	1.08		[56]
Proportion of population 18+ studying post-secondary education ratio: rest of Australia vs QLD	1.013012		[56]

Prevalence of moderate to very high psychological distress ratio secondary students vs population	1		Assumes that the prevalence of psychological distress in secondary students is the same as population prevalence for 12-17 year olds.
Effect of moderate to very high distress on discontinuation of secondary education	1.99		[6]
Prevalence of moderate to very high psychological distress ratio post-secondary students vs population	1.3995		[15]
Effect of psychological distress on discontinuation of post-secondary education	1.1		[7]
**Education – Highest qualifications**			
Proportion of population with secondary qualification only initial by age 15-24, 25+	15-24 years	0.404604512	Estimated with constrained optimisation using qualifications, education and work statistics from the ABS [11].
	25 years and older	0.172317986	
Proportion of population with post-secondary qualification initial by age 15-24, 25+	15-24 years	0.29891598	Estimated with constrained optimisation using qualifications, education and work statistics from the ABS [11].
	25 years and older	0.40157251	
Proportion completing first post-secondary qualification by age 15-24, 25+	15-24 years	0.941009619	Estimated with constrained optimisation using qualifications, education and work statistics from the ABS [11].
	25 years and older	0.273396566	
Proportion of secondary school graduates who are aged 15-24	0.528945706		Estimated with constrained optimisation using qualifications, education and work statistics from the ABS [11].
Proportion of post-secondary graduates who are aged 15-24	0.081662928		Estimated with constrained optimisation using qualifications, education and work statistics from the ABS [11].
Death rate ratio post-secondary qualification vs low education	0.3623188		[12]
Death rate ratio secondary qualification only vs low education	0.635		[12]
Proportion of population with secondary qualification only ratio Australia vs BSPHN	15-24 years	1.1184972	[1, 11]
	25 years and older	1.0674508	
Proportion of population with post-secondary qualification ratio Australia vs BSPHN	15-24 years	0.8848893	[1, 11]
	25 years and older	1.0140127	
**Labour force**			
Proportion of population 15-24, 25+ sufficiently employed proportion initial	15-24 years	0.645484041	Estimated with constrained optimisation using labour force statistics from the ABS [11, 17, 18].
	25 years and older	0.671011922	
Proportion of population 15-24, 25+ underemployed proportion initial	15-24 years	0.000004229	Estimated with constrained optimisation using labour force statistics from the ABS [11, 17, 18].
	25 years and older	0.000610871	
Proportion of population 15-24, 25+ unemployed proportion initial	15-24 years	0.112349271	Estimated with constrained optimisation using labour force statistics from the ABS [11, 17, 18].
	25 years and older	0.033182918	
Sufficiently employed to unemployed per capita per year base rate by age 15-24, 25+	15-24 years	0.188773316	Estimated with constrained optimisation using labour force statistics from the ABS [11, 17, 18].
	25 years and older	0.041900295	
Unemployed to sufficiently employed per capita per year base rate by age 15-24, 25+	15-24 years	1.966435759	Estimated with constrained optimisation using labour force statistics from the ABS [11, 17, 18].
	25 years and older	0.851576955	
	15-24 years	0.086418873	

Underemployed to unemployed per capita per year base rate by age 15-24, 25+	25 years and older	0.533308196	Estimated with constrained optimisation using labour force statistics from the ABS [11, 17, 18].
Unemployed to underemployed per capita per year base rate by age 15-24, 25+	15-24 years	1.499279338	Estimated with constrained optimisation using labour force statistics from the ABS [11, 17, 18].
	25 years and older	2.25377314	
Sufficiently employed to underemployed per capita per year base rate by age 15-24, 25+	15-24 years	0.062465237	Estimated with constrained optimisation using labour force statistics from the ABS [11, 17, 18].
	25 years and older	0.080178673	
Underemployed to sufficiently employed per capita per year base rate by age 15-24, 25+	15-24 years	0.95585229	Estimated with constrained optimisation using labour force statistics from the ABS [11, 17, 18].
	25 years and older	1.378794293	
Unemployed to NILF per capita per year base rate by age 15-24, 25+	15-24 years	3.031715245	Estimated with constrained optimisation using labour force statistics from the ABS [11, 17, 18].
	25 years and older	2.430982644	
NILF to Unemployed per capita per year base rate by age 15-24, 25+	15-24 years	1.435570397	Estimated with constrained optimisation using labour force statistics from the ABS [11, 17, 18].
	25 years and older	0.268136312	
Sufficiently employed to NILF per capita per year base rate by age 15-24, 25+	15-24 years	0.420654638	Estimated with constrained optimisation using labour force statistics from the ABS [11, 17, 18].
	25 years and older	0.065116267	
Underemployed to NILF per capita per year base rate by age 15-24, 25+	15-24 years	0.049159652	Estimated with constrained optimisation using labour force statistics from the ABS [11, 17, 18].
	25 years and older	0.04791504	
Proportion of population sufficiently employed ratio: rest of Australia vs QLD	0.9999229		[18]
Proportion of population underemployed ratio: rest of Australia vs QLD	0.9548649		[18]
Proportion of population unemployed ratio: rest of Australia vs BSPHN	0.9051092		[11]
Death rate ratio: unemployed vs employed	1.22		[13]
Effect of post-secondary qualification on underemployment to sufficiently employed rate	1.407043821		[16]
Post-secondary qualification probability ratio underemployed vs population	0.86282872		[16]
Effect of moderate to very high distress on employment	0.8396596		[14]
Moderate distress prevalence ratio unemployed by age 15-24, 25+	15-24 years	1.34	[15]
	25 years and older	1.73	
Moderate distress prevalence ratio participation by age 15-24, 25+	15-24 years	0.981	[15]
	25 years and older	0.924	
Effect of post-secondary qualification on participation	1.435808		[11]
Post-secondary qualifications prevalence ratio participation vs population	1.08		[11]
Effect of post-secondary qualification on employment vs low educational attainment	1.512015		[11]
Post-secondary qualifications prevalence ratio unemployed vs population	0.822		[11]

Effect of secondary qualification only on participation	1.281302		[11]
Secondary qualifications only prevalence ratio participation vs population	0.964		[11]
Effect of secondary qualification only on employment vs low educational attainment	1.305415		[11]
Secondary qualifications only prevalence ratio unemployed vs population	1.29		[11]
**NEET**			
Proportion of population NEET ratio 15-17 / 15-24	0.2893055		[57]
Coefficient for students aged 15-24 and not employed initial	1.711780643		Estimated with constrained optimisation using education and work statistics from the ABS [11].
Coefficient for students aged 15-24 and not employed increase per year	0.046446351		Estimated with constrained optimisation using education and work statistics from the ABS [11].
**Psychological distress / disorder**			
Prevalence of moderate to very high distress initial by age 12+	12-14 years	0.307415879	Estimated with constrained optimisation using psychological distress prevalence data from the ABS [15, 26, 27] and the Young Minds Matter Survey [28], and 12-month psychological disorder prevalence data from the ABS [29, 30].
	15-17 years	0.506342413	
	18-24 years	0.19807616	
	25 years and older	0.336014894	
Coefficient Social cohesion on Distress onset per year per base rate by age 12+	12-14 years	-0.107442807	Estimated with constrained optimisation using psychological distress prevalence data from the ABS [15, 26, 27] and the Young Minds Matter Survey [28], and 12-month psychological disorder prevalence data from the ABS [29, 30].
	15-17 years	-0.019644493	
	18-24 years	-0.028448839	
	25 years and older	-0.032940071	
Intercept social cohesion on Distress onset per year per base rate by age 12+	12-14 years	0.049399265	Estimated with constrained optimisation using psychological distress prevalence data from the ABS [15, 26, 27] and the Young Minds Matter Survey [28], and 12-month psychological disorder prevalence data from the ABS [29, 30].
	15-17 years	0.060668244	
	18-24 years	-0.002286305	
	25 years and older	0.034026888	
Coefficient social cohesion on Disorder incidence per year base rate by age 12+	12-14 years	-0.116738035	Estimated with constrained optimisation using psychological distress prevalence data from the ABS [15, 26, 27] and the Young Minds Matter Survey [28], and 12-month psychological disorder prevalence data from the ABS [29, 30].
	15-17 years	-0.027401149	
	18-24 years	-0.027628572	
	25 years and older	-0.026413605	
Intercept social cohesion on Disorder incidence per year base rate by age 12+	12-14 years	0.974258794	Estimated with constrained optimisation using psychological distress prevalence data from the ABS [15, 26, 27] and the Young Minds Matter Survey [28], and 12-month psychological disorder prevalence data from the ABS [29, 30].
	15-17 years	0.993189929	
	18-24 years	1.014336705	
	25 years and older	0.979776148	
Death rate ratio Moderate distress vs Low distress	1.16		[25]
Death rate ratio High distress vs Low distress	1.37		[25]
Prevalence of low distress ratio Australia vs QLD by age 12+	12-14 years	1	[15]
	15-17 years	1	

	18-24 years	1.01	
	25 years and older	1.02	
Prevalence of disorder ratio Australia vs QLD by age 12+	12-14 years	1	[15]
	15-17 years	1	
	18-24 years	0.983	
	25 years and older	0.958	
Effect of unemployment on distress	15-24 years	1.43	[20]
	25 years and older	1.81	
Unemployment rate ratio of low distress vs population by age 15-24, 25+	15-24 years	0.759	[20]
	25 years and older	0.666	
Effect of homelessness on distress	2.14		[20]
Homelessness prevalence ratio of low distress vs population	0.567		[20]
Effect of underemployment on distress	1.132448		[21]
Underemployment ratio of low distress vs population	1.003201		[22]
Effect of substance abuse on distress	2.63		[23]
Substance misuse prevalence ratio of low distress vs population	0.6595638		[24]
Distress recovery per year base rate by age 12+	0.06833333		[58]
Disorder recovery per year base rate by age 12+	0.06833333		[58]
Proportion of disorder recovery to no disorder, moderate to very high distress through treatment	0.8		Assumes that 80% of people with a mental disorder who recover through accessing services will recover to a state of moderate to very high psychological distress and that 20% will recover into a state of low psychological distress.
Proportion of Close to average SDQ to Low distress	0.468		[31]
Proportion of Close to average SDQ to Disorder, Moderate to Very high distress	0.238		[31]
Proportion of Slightly raised SDQ to Low distress	0.182		[31]
Proportion of Slightly raised SDQ to Disorder, Moderate to Very high distress	0.62		[31]
Proportion of High SDQ to Low distress	0.112		[31]
Proportion of High SDQ to Disorder, Moderate to Very high distress	0.72		[31]
**Strengths and Difficulties**			
Coefficient Social cohesion on Close to average to Slightly raised SDQ per year base rate by age <12	0-4 years	-0.042484533	Estimated with constrained optimisation using SDQ data from LSAC [31].
	5-11 years	-0.044012565	
	0-4 years	1.242650711	Estimated with constrained optimisation using SDQ data from LSAC [31].

Intercept social cohesion on Close to average to Slightly raised SDQ per year base rate by age <12	5-11 years	1.041291164	
Coefficient Social cohesion on Slightly raised to High SDQ per year base rate by age <12	0-4 years	-0.017355267	Estimated with constrained optimisation using SDQ data from LSAC [31].
	5-11 years	-0.038128501	
Intercept social cohesion on Slightly raised to High SDQ per year base rate by age <12	0-4 years	0.360948024	Estimated with constrained optimisation using SDQ data from LSAC [31].
	5-11 years	2.28262466	
Proportion of High SDQ recovery to Slightly raised through treatment	0.8		Assumes that 80% of people with High levels of SDQ who recover through accessing services will recover to a state of Slightly raised SDQ and that 20% will recover into a state of Close to average SDQ.
Prevalence of slightly raised SDQ ratio rest of Australia vs BSPHN by age <12	0-4 years	Not shown here	Please note that, as part of the user agreement between the authors and LSAC, SDQ data at the PHN level of geographic granularity cannot be shown [31]
	5-11 years	Not shown here	
Prevalence of high SDQ ratio rest of Australia vs BSPHN by age <12	0-4 years	Not shown here	Please note that, as part of the user agreement between the authors and LSAC, SDQ data at the PHN level of geographic granularity cannot be shown [31]
	5-11 years	Not shown here	
Slightly raised to Close to average SDQ per year base rate by age <12	0.06833333		[58]
High to Slightly raised SDQ per year base rate by age <12	0.06833333		[58]
**Homelessness**			
Homeless by age initial	0–4 years	230.3500687	Estimated with constrained optimisation using homelessness statistics from the ABS [35, 36].
	5–11 years	258.4868751	
	12-14 years	101.3063039	
	15-17 years	167.9467548	
	18-24 years	716.0155577	
	25 years and older	2298.728691	
Entering homelessness per week base rate by age	0–4 years	0.000027358	Estimated with constrained optimisation using homelessness statistics from the ABS [35, 36].
	5–11 years	0.000015254	
	12-14 years	0.000012205	
	15-17 years	0.000014189	
	18-24 years	0.000023220	
	25 years and older	0.000011581	
Mean duration of homelessness	239.8571		[34]
Death rate ratio homeless vs non-homeless	1.6		[32]
Effect of unemployment on entering homelessness	2.6		[33]
Effect of mental illness on entering homelessness	1.7		[33]
Effect of substance misuse on entering homelessness	2.3		[33]
**Substance misuse**			
	15-24 years	980.4253188	

Waiting for substance misuse treatment by age 15-24, 25+ initial	25 years and older	974.8870492	Estimated with constrained optimisation using national 12-month substance use disorder data from the ABS [29, 30] and substance misuse services data from AIHW [43, 44].
Substance misuse seeking treatment per capita per week rate initial	15-24 years	0.002209145	Estimated with constrained optimisation using national 12-month substance use disorder data from the ABS [29, 30] and substance misuse services data from AIHW [43, 44].
	25 years and older	0.001545306	
Substance misuse seeking treatment per capita per week increase per year	15-24 years	0.000131204	Estimated with constrained optimisation using national 12-month substance use disorder data from the ABS [29, 30] and substance misuse services data from AIHW [43, 44].
	25 years and older	0.000485677	
Substance misuse onset per capita per week base rate by age 15-24, 25+	15-24 years	0.000159647	Estimated with constrained optimisation using national 12-month substance use disorder data from the ABS [29, 30] and substance misuse services data from AIHW [43, 44].
	25 years and older	0.000022895	
Substance misuse natural recovery per capita per week base rate by age 15-24, 25+	15-24 years	0.000914274	Estimated with constrained optimisation using national 12-month substance use disorder data from the ABS [29, 30] and substance misuse services data from AIHW [43, 44].
	25 years and older	0.002283660	
Substance misuse treatment capacity per week initial	76.20995643		Estimated with constrained optimisation using national 12-month substance use disorder data from the ABS [29, 30] and substance misuse services data from AIHW [43, 44].
Substance misuse treatment capacity per week increase per year	8.648468568		Estimated with constrained optimisation using national 12-month substance use disorder data from the ABS [29, 30] and substance misuse services data from AIHW [43, 44].
Substance misuse treatment mean duration	4.140022		[44]
Prevalence of substance misuse ratio rest of Australia vs BSPHN by age 15-24, 25+	1		This sector was calibrated with national prevalence data, hence the prevalence ratio will be one. [30, 59]
Death rate ratio substance misuse vs no substance misuse	1.95		[37]
Effect of homelessness on substance misuse	1.65		[39]
Prevalence of homelessness in the non substance misuse population vs total population	0.8683555		[40]
Effect of moderate to very high distress on substance misuse	2.505036		[38]
Prevalence of distress in the non substance misuse population vs total population	0.9916363		[38]
Effect of NEET substance misuse	1.43		[41]
Prevalence of NEET in the non substance misuse population vs total population	1.012717083		[30]
QLD average number of closed treatment episodes per client	1.3		[43]
Recovery rate from substance misuse treatment	0.359		[60]
**Suicidal behaviours**			
Suicide attempt lethality 0-24	0.023634286		Estimated with constrained optimisation using suicide deaths statistics from the AIHW [47] and intentional self-harm hospitalisations statistics provided by Queensland Health [48].
Index suicide attempt base rate by age 12+	12-14 years	0.000014123825	Estimated with constrained optimisation using suicide deaths statistics from the AIHW [47] and intentional self-harm hospitalisations statistics provided by Queensland Health [48].
	15-17 years	0.000024350493	
	18-24 years	0.000025232155	
	25 years and older	0.000016279156	

Repeat suicide attempts per week base rate	0.006285649		Estimated with constrained optimisation using suicide deaths statistics from the AIHW [47] and intentional self-harm hospitalisations statistics provided by Queensland Health [48].
Suicide attempt lethality ratio 0-24 vs 25+	3.217148569		[61, 62]
Suicide rate ratio substance misuse disorder vs no substance misuse disorder	4.1		[46]
Suicide attempt rate ratio by distress	No distress	1 (Reference)	[45]
	Distress No Disorder	1.41	
	Distress Disorder	3.57	
**Mental health services**			
Effect of disengagement on increasing psychological distress	1.271517		[24]
Baseline disengagement rate waiting per year	0.2620284		[63]
Baseline disengagement rate hospital care	0.051642558		[64]
Baseline disengagement rate non-hospital care	0.03909747		[64]
Effect of distress on hospitalisation	No distress	0	[20]
	Distress No Disorder	1 (Reference)	
	Distress Disorder	1.773723	
Mean treatment duration psychiatric hospital care	1.997143		[65]
Mean treatment duration non-specialised hospital care	0.7142857		[65]
Mean treatment duration online services	6		[66]
Seeking help to online services rate by age	0–4 years	0.0440037	[30]
	5–11 years	0.0440037	
	12-14 years	0.079675	
	15-17 years	0.079675	
	18-24 years	0.079675	
	25 years and older	0.027663	
Referral rate from GP to headspace	0–4 years	0	[50, 67]
	5–11 years	0	
	12-14 years	0.07415457	
	15-17 years	0.07415457	
	18-24 years	0.07415457	
	25 years and older	0	
Referral rate from GP to other services	0.046749		[68]
Effect of distress on help-seeking with GP	No distress	0	[20]
	Distress No Disorder	1	
	Distress Disorder	3.287037	

Proportion of post-discharge referrals to non-CMHC services to GP	0.5		Assumes half of patients not referred to CMHC services after discharge from hospital care are referred to a general practitioner. The remaining patients (i.e., those not referred to CMHC services or a general practitioner) are referred to a psychiatrist or allied mental health professional
Effect of distress on referral rate	No distress	0	[20]
	Distress No Disorder	1	
	Distress Disorder	1.786096	
Effect of distress on seeking help psychiatrist or allied health services	No distress	0	[20]
	Distress No Disorder	1	
	Distress Disorder	4.398422	
Baseline recovery rate psychiatric hospital care	No distress	0	[69]
	Distress No Disorder	0.4241071	
	Distress Disorder	0.3712737	
Baseline recovery rate headspace	0.050295858		[67]
Baseline recovery rate CMHC services	0.02332282		[70]
Recovery rate ratio GP services	No distress	0	[71]
	Distress No Disorder	1	
	Distress Disorder	0.4626866	
Baseline recovery rate mental health treatment	No distress	0	[69]
	Distress No Disorder	0.09525994	
	Distress Disorder	0.08339287	
Psychological treatment rate GP services	0.48343875		[70]
Recovery rate online services	No distress	0	[66, 72]
	Distress No Disorder	0.4	
	Distress Disorder	0.1850746	
Perceived needs for services per capita per week rate initial	No distress	0	Estimated with constrained optimisation using mental health service engagement data from the ABS [20], mental health perfomance indicator data from AIHW [52], Medicare-subsidised primary mental health services data from AIHW [50], CMHC service contacts data from AIHW [51], headspace occasions of service data provided by BSPHN, mental health related ED presentations data provided by Queensland Health [48] and episodes of admitted care data provided by Queensland Health [48].
	Distress No Disorder	0.001805767	
	Distress Disorder	0.012469428	
Perceived needs for services rate increase per year	No distress	0	Estimated with constrained optimisation using mental health service engagement data from the ABS [20], mental health perfomance indicator data from AIHW [52], Medicare-subsidised primary mental health services data from AIHW [50], CMHC service contacts data from AIHW [51], headspace occasions of service data provided by BSPHN, mental health related ED presentations data provided by Queensland Health [48] and episodes of admitted care data provided by Queensland Health [48].
	Distress No Disorder	-3.32927E-05	
	Distress Disorder	7.80161E-05	
Re-engaging excluding ED per capita per week rate initial	No distress	0	Estimated with constrained optimisation using mental health service engagement data from the ABS [20], mental health perfomance indicator data from AIHW [52], Medicare-subsidised primary mental health services data from AIHW [50], CMHC service contacts data from AIHW [51], headspace occasions of service data provided by BSPHN, mental
	Distress No Disorder	0.113266016	
	Distress Disorder	0.095776677	

			health related ED presentations data provided by Queensland Health [48] and episodes of admitted care data provided by Queensland Health [48].
Re-engaging excluding ED per capita per week rate increase per year	No distress	0	Estimated with constrained optimisation using mental health service engagement data from the ABS [20], mental health perfomance indicator data from AIHW [52], Medicare-subsidised primary mental health services data from AIHW [50], CMHC service contacts data from AIHW [51], headspace occasions of service data provided by BSPHN, mental health related ED presentations data provided by Queensland Health [48] and episodes of admitted care data provided by Queensland Health [48].
	Distress No Disorder	0.002808987	
	Distress Disorder	0.085153265	
Proportion of population in distress seeking help initial by distress	No distress	0	Estimated with constrained optimisation using mental health service engagement data from the ABS [20], mental health perfomance indicator data from AIHW [52], Medicare-subsidised primary mental health services data from AIHW [50], CMHC service contacts data from AIHW [51], headspace occasions of service data provided by BSPHN, mental health related ED presentations data provided by Queensland Health [48] and episodes of admitted care data provided by Queensland Health [48].
	Distress No Disorder	0.195389983	
	Distress Disorder	0.652438251	
Disengaged initial total	0		Estimated with constrained optimisation using mental health service engagement data from the ABS [20], mental health perfomance indicator data from AIHW [52], Medicare-subsidised primary mental health services data from AIHW [50], CMHC service contacts data from AIHW [51], headspace occasions of service data provided by BSPHN, mental health related ED presentations data provided by Queensland Health [48] and episodes of admitted care data provided by Queensland Health [48].
GP services capacity per week initial	2058.304273		Estimated with constrained optimisation using mental health service engagement data from the ABS [20], mental health perfomance indicator data from AIHW [52], Medicare-subsidised primary mental health services data from AIHW [50], CMHC service contacts data from AIHW [51], headspace occasions of service data provided by BSPHN, mental health related ED presentations data provided by Queensland Health [48] and episodes of admitted care data provided by Queensland Health [48].
GP services capacity per week increase per year	140.5472373		Estimated with constrained optimisation using mental health service engagement data from the ABS [20], mental health perfomance indicator data from AIHW [52], Medicare-subsidised primary mental health services data from AIHW [50], CMHC service contacts data from AIHW [51], headspace occasions of service data provided by BSPHN, mental health related ED presentations data provided by Queensland Health [48] and episodes of admitted care data provided by Queensland Health [48].
Psychiatrist and allied health services capacity per week initial	5342.906405		Estimated with constrained optimisation using mental health service engagement data from the ABS [20], mental health perfomance indicator data from AIHW [52], Medicare-subsidised primary mental health services data from AIHW [50], CMHC service contacts data from AIHW [51], headspace occasions of service data provided by BSPHN, mental health related ED presentations data provided by Queensland Health [48] and episodes of admitted care data provided by Queensland Health [48].
Psychiatrist and allied health services capacity per week increase per year	298.2331118		Estimated with constrained optimisation using mental health service engagement data from the ABS [20], mental health perfomance indicator data from AIHW [52], Medicare-subsidised primary mental health services data from AIHW [50], CMHC service contacts data from AIHW [51], headspace occasions of service data provided by BSPHN, mental

		health related ED presentations data provided by Queensland Health [48] and episodes of admitted care data provided by Queensland Health [48].
CYMHS services capacity per week initial	1147.346	Estimated with constrained optimisation using mental health service engagement data from the ABS [20], mental health perfomance indicator data from AIHW [52], Medicare-subsidised primary mental health services data from AIHW [50], CMHC service contacts data from AIHW [51], headspace occasions of service data provided by BSPHN, mental health related ED presentations data provided by Queensland Health [48] and episodes of admitted care data provided by Queensland Health [48].
CYMHS services capacity per week increase per year	89.01923	Estimated with constrained optimisation using mental health service engagement data from the ABS [20], mental health perfomance indicator data from AIHW [52], Medicare-subsidised primary mental health services data from AIHW [50], CMHC service contacts data from AIHW [51], headspace occasions of service data provided by BSPHN, mental health related ED presentations data provided by Queensland Health [48] and episodes of admitted care data provided by Queensland Health [48].
CMHC services (adult) capacity per week initial	4816.154	Estimated with constrained optimisation using mental health service engagement data from the ABS [20], mental health perfomance indicator data from AIHW [52], Medicare-subsidised primary mental health services data from AIHW [50], CMHC service contacts data from AIHW [51], headspace occasions of service data provided by BSPHN, mental health related ED presentations data provided by Queensland Health [48] and episodes of admitted care data provided by Queensland Health [48].
CMHC services (adult) capacity per week increase per year	222.3462	Estimated with constrained optimisation using mental health service engagement data from the ABS [20], mental health perfomance indicator data from AIHW [52], Medicare-subsidised primary mental health services data from AIHW [50], CMHC service contacts data from AIHW [51], headspace occasions of service data provided by BSPHN, mental health related ED presentations data provided by Queensland Health [48] and episodes of admitted care data provided by Queensland Health [48].
Psychiatric hospitalisation capacity per week initial	90.5	Estimated with constrained optimisation using mental health service engagement data from the ABS [20], mental health perfomance indicator data from AIHW [52], Medicare-subsidised primary mental health services data from AIHW [50], CMHC service contacts data from AIHW [51], headspace occasions of service data provided by BSPHN, mental health related ED presentations data provided by Queensland Health [48] and episodes of admitted care data provided by Queensland Health [48].
Psychiatric hospitalisation capacity per week increase per year	4.412692	Estimated with constrained optimisation using mental health service engagement data from the ABS [20], mental health perfomance indicator data from AIHW [52], Medicare-subsidised primary mental health services data from AIHW [50], CMHC service contacts data from AIHW [51], headspace occasions of service data provided by BSPHN, mental health related ED presentations data provided by Queensland Health [48] and episodes of admitted care data provided by Queensland Health [48].
Non-specialised hospital care services capacity per week initial	206.883489	Estimated with constrained optimisation using mental health service engagement data from the ABS [20], mental health perfomance indicator data from AIHW [52], Medicare-subsidised primary mental health services data from AIHW [50], CMHC service contacts data from AIHW [51], headspace occasions of service data provided by BSPHN, mental

			health related ED presentations data provided by Queensland Health [48] and episodes of admitted care data provided by Queensland Health [48].
Non-specialised hospital care services capacity capacity per week increase per year	35.86016039		Estimated with constrained optimisation using mental health service engagement data from the ABS [20], mental health perfomance indicator data from AIHW [52], Medicare-subsidised primary mental health services data from AIHW [50], CMHC service contacts data from AIHW [51], headspace occasions of service data provided by BSPHN, mental health related ED presentations data provided by Queensland Health [48] and episodes of admitted care data provided by Queensland Health [48].
headspace services capacity per week initial	1		Estimated with constrained optimisation using mental health service engagement data from the ABS [20], mental health perfomance indicator data from AIHW [52], Medicare-subsidised primary mental health services data from AIHW [50], CMHC service contacts data from AIHW [51], headspace occasions of service data provided by BSPHN, mental health related ED presentations data provided by Queensland Health [48] and episodes of admitted care data provided by Queensland Health [48].
headspace services capacity capacity per week increase per year	45.39721129		Estimated with constrained optimisation using mental health service engagement data from the ABS [20], mental health perfomance indicator data from AIHW [52], Medicare-subsidised primary mental health services data from AIHW [50], CMHC service contacts data from AIHW [51], headspace occasions of service data provided by BSPHN, mental health related ED presentations data provided by Queensland Health [48] and episodes of admitted care data provided by Queensland Health [48].
ED presentations per capita per week rate by age initial	0–4 years	0.00000620315	Estimated with constrained optimisation using mental health service engagement data from the ABS [20], mental health perfomance indicator data from AIHW [52], Medicare-subsidised primary mental health services data from AIHW [50], CMHC service contacts data from AIHW [51], headspace occasions of service data provided by BSPHN, mental health related ED presentations data provided by Queensland Health [48] and episodes of admitted care data provided by Queensland Health [48].
	5–11 years	0.00003095921	
	12-14 years	0.00018241607	
	15-17 years	0.00030566461	
	18-24 years	0.00062163940	
	25 years and older	0.00048565391	
ED presentations rate by age increase per year	0–4 years	0.00000614195	Estimated with constrained optimisation using mental health service engagement data from the ABS [20], mental health perfomance indicator data from AIHW [52], Medicare-subsidised primary mental health services data from AIHW [50], CMHC service contacts data from AIHW [51], headspace occasions of service data provided by BSPHN, mental health related ED presentations data provided by Queensland Health [48] and episodes of admitted care data provided by Queensland Health [48].
	5–11 years	0.00000703190	
	12-14 years	0.00001119396	
	15-17 years	0.00000543526	
	18-24 years	-0.00001699589	
	25 years and older	-0.00001301185	
ED presentation rate ratio help seeker	1		Estimated with constrained optimisation using mental health service engagement data from the ABS [20], mental health perfomance indicator data from AIHW [52], Medicare-subsidised primary mental health services data from AIHW [50], CMHC service contacts data from AIHW [51], headspace occasions of service data provided by BSPHN, mental health related ED presentations data provided by Queensland Health [48] and episodes of admitted care data provided by Queensland Health [48].
ED to admission rate initial by age	0–4 years	0.218855977	Estimated with constrained optimisation using mental health service engagement data from the ABS [20], mental health perfomance indicator data from AIHW [52], Medicare-subsidised primary mental health services data from AIHW [50], CMHC service contacts data from AIHW [51], headspace occasions of service data provided by BSPHN, mental
	5–11 years	0.125828686	
	12-14 years	0.191485232	
	15-17 years	0.249542276	

	18-24 years	0.08105427	health related ED presentations data provided by Queensland Health [48] and episodes of admitted care data provided by Queensland Health [48].
	25 years and older	0.145228354	
ED to admission rate increase per year	0–4 years	-0.011687394	Estimated with constrained optimisation using mental health service engagement data from the ABS [20], mental health perfomance indicator data from AIHW [52], Medicare-subsidised primary mental health services data from AIHW [50], CMHC service contacts data from AIHW [51], headspace occasions of service data provided by BSPHN, mental health related ED presentations data provided by Queensland Health [48] and episodes of admitted care data provided by Queensland Health [48].
	5–11 years	0.004505288	
	12-14 years	0.010480664	
	15-17 years	-0.001778161	
	18-24 years	0.072187184	
	25 years and older	0.086166213	
Proportion of admissions into psychiatric hospitalisation by age 0-17, 18-24, 25+	0-17 years	0.414161436	Estimated with constrained optimisation using mental health service engagement data from the ABS [20], mental health perfomance indicator data from AIHW [52], Medicare-subsidised primary mental health services data from AIHW [50], CMHC service contacts data from AIHW [51], headspace occasions of service data provided by BSPHN, mental health related ED presentations data provided by Queensland Health [48] and episodes of admitted care data provided by Queensland Health [48].
	18-24 years	0.547643051	
	25 years and older	0.377282299	
Additional non-specialised hospitalisations per capita per week by age 0-17, 18-24, 25+	0-17 years	0.00023579	Estimated with constrained optimisation using mental health service engagement data from the ABS [20], mental health perfomance indicator data from AIHW [52], Medicare-subsidised primary mental health services data from AIHW [50], CMHC service contacts data from AIHW [51], headspace occasions of service data provided by BSPHN, mental health related ED presentations data provided by Queensland Health [48] and episodes of admitted care data provided by Queensland Health [48].
	18-24 years	0.000773031	
	25 years and older	0.00189995	
Referral rate from ED to CMHC	0.074017861		Estimated with constrained optimisation using mental health service engagement data from the ABS [20], mental health perfomance indicator data from AIHW [52], Medicare-subsidised primary mental health services data from AIHW [50], CMHC service contacts data from AIHW [51], headspace occasions of service data provided by BSPHN, mental health related ED presentations data provided by Queensland Health [48] and episodes of admitted care data provided by Queensland Health [48].
Waiting for psychiatric hospitalisation initial total	0		Estimated with constrained optimisation using mental health service engagement data from the ABS [20], mental health perfomance indicator data from AIHW [52], Medicare-subsidised primary mental health services data from AIHW [50], CMHC service contacts data from AIHW [51], headspace occasions of service data provided by BSPHN, mental health related ED presentations data provided by Queensland Health [48] and episodes of admitted care data provided by Queensland Health [48].
Seeking help GP services rate by age 0-14, 15-24, 25+ initial	0-14 years	0.003158381	Estimated with constrained optimisation using mental health service engagement data from the ABS [20], mental health perfomance indicator data from AIHW [52], Medicare-subsidised primary mental health services data from AIHW [50], CMHC service contacts data from AIHW [51], headspace occasions of service data provided by BSPHN, mental health related ED presentations data provided by Queensland Health [48] and episodes of admitted care data provided by Queensland Health [48].
	15-24 years	0.005169437	
	25 years and older	0.007613575	
Seeking help GP services rate by age 0-14, 15-24, 25+ increase per year	0-14 years	2.99577E-06	Estimated with constrained optimisation using mental health service engagement data from the ABS [20], mental health perfomance indicator data from AIHW [52], Medicare-subsidised primary mental health services data from AIHW [50], CMHC service contacts data from AIHW [51], headspace occasions of service data provided by BSPHN, mental
	15-24 years	0.000307391	
	25 years and older	1.00601E-05	

			health related ED presentations data provided by Queensland Health [48] and episodes of admitted care data provided by Queensland Health [48].
Waiting for GP initial total	4260.483118		Estimated with constrained optimisation using mental health service engagement data from the ABS [20], mental health perfomance indicator data from AIHW [52], Medicare-subsidised primary mental health services data from AIHW [50], CMHC service contacts data from AIHW [51], headspace occasions of service data provided by BSPHN, mental health related ED presentations data provided by Queensland Health [48] and episodes of admitted care data provided by Queensland Health [48].
Referral rate from GP to psychiatrist and allied health service initial	0.045365473		Estimated with constrained optimisation using mental health service engagement data from the ABS [20], mental health perfomance indicator data from AIHW [52], Medicare-subsidised primary mental health services data from AIHW [50], CMHC service contacts data from AIHW [51], headspace occasions of service data provided by BSPHN, mental health related ED presentations data provided by Queensland Health [48] and episodes of admitted care data provided by Queensland Health [48].
Referral rate from GP to psychiatrist and allied health service increase per year	0.004929958		Estimated with constrained optimisation using mental health service engagement data from the ABS [20], mental health perfomance indicator data from AIHW [52], Medicare-subsidised primary mental health services data from AIHW [50], CMHC service contacts data from AIHW [51], headspace occasions of service data provided by BSPHN, mental health related ED presentations data provided by Queensland Health [48] and episodes of admitted care data provided by Queensland Health [48].
Referral rate from GP to CMHC	0.003341101		Estimated with constrained optimisation using mental health service engagement data from the ABS [20], mental health perfomance indicator data from AIHW [52], Medicare-subsidised primary mental health services data from AIHW [50], CMHC service contacts data from AIHW [51], headspace occasions of service data provided by BSPHN, mental health related ED presentations data provided by Queensland Health [48] and episodes of admitted care data provided by Queensland Health [48].
Additional psychiatrist or allied health services per capita per week initial by age 0-14, 15-24, 25+	0-14 years	0.014226087	Estimated with constrained optimisation using mental health service engagement data from the ABS [20], mental health perfomance indicator data from AIHW [52], Medicare-subsidised primary mental health services data from AIHW [50], CMHC service contacts data from AIHW [51], headspace occasions of service data provided by BSPHN, mental health related ED presentations data provided by Queensland Health [48] and episodes of admitted care data provided by Queensland Health [48].
	15-24 years	0.008226495	
	25 years and older	0.027601801	
Additional psychiatrist or allied health services per capita per week increase per year by age 0-14, 15-24, 25+	0-14 years	-0.000439327	Estimated with constrained optimisation using mental health service engagement data from the ABS [20], mental health perfomance indicator data from AIHW [52], Medicare-subsidised primary mental health services data from AIHW [50], CMHC service contacts data from AIHW [51], headspace occasions of service data provided by BSPHN, mental health related ED presentations data provided by Queensland Health [48] and episodes of admitted care data provided by Queensland Health [48].
	15-24 years	0.00126023	
	25 years and older	-0.000438443	
Referral rate from psychiatrist and allied health to psychiatric hospital care services by age 0-17, 18-24, 25+	0-17 years	0.003455588	Estimated with constrained optimisation using mental health service engagement data from the ABS [20], mental health perfomance indicator data from AIHW [52], Medicare-subsidised primary mental health services data from AIHW [50], CMHC service contacts data from AIHW [51], headspace occasions of service data provided by BSPHN, mental
	18-24 years	0.008682608	
	25 years and older	0.007059504	

			health related ED presentations data provided by Queensland Health [48] and episodes of admitted care data provided by Queensland Health [48].
Waiting for psychiatrist and allied health initial total	41729.37681		Estimated with constrained optimisation using mental health service engagement data from the ABS [20], mental health perfomance indicator data from AIHW [52], Medicare-subsidised primary mental health services data from AIHW [50], CMHC service contacts data from AIHW [51], headspace occasions of service data provided by BSPHN, mental health related ED presentations data provided by Queensland Health [48] and episodes of admitted care data provided by Queensland Health [48].
Referral rate from post-discharge to CMHC services initial	0–4 years	0.735839126	Estimated with constrained optimisation using mental health service engagement data from the ABS [20], mental health perfomance indicator data from AIHW [52], Medicare-subsidised primary mental health services data from AIHW [50], CMHC service contacts data from AIHW [51], headspace occasions of service data provided by BSPHN, mental health related ED presentations data provided by Queensland Health [48] and episodes of admitted care data provided by Queensland Health [48].
	5–11 years	0.66178922	
	12-14 years	0.721061818	
	15-17 years	0.697091783	
	18-24 years	0.701704305	
	25 years and older	0.777140465	
Referral rate from post-discharge to CMHC services increase per year	0–4 years	0.003500216	Estimated with constrained optimisation using mental health service engagement data from the ABS [20], mental health perfomance indicator data from AIHW [52], Medicare-subsidised primary mental health services data from AIHW [50], CMHC service contacts data from AIHW [51], headspace occasions of service data provided by BSPHN, mental health related ED presentations data provided by Queensland Health [48] and episodes of admitted care data provided by Queensland Health [48].
	5–11 years	0.053376831	
	12-14 years	0.010040507	
	15-17 years	0.023416686	
	18-24 years	0.02094671	
	25 years and older	-0.0006888	
Additional CMHC per capita per week initial by age AIHW	0–4 years	0.006479574	Estimated with constrained optimisation using mental health service engagement data from the ABS [20], mental health perfomance indicator data from AIHW [52], Medicare-subsidised primary mental health services data from AIHW [50], CMHC service contacts data from AIHW [51], headspace occasions of service data provided by BSPHN, mental health related ED presentations data provided by Queensland Health [48] and episodes of admitted care data provided by Queensland Health [48].
	5–11 years	0.034805066	
	12-17 years	0.092108971	
	18-24 years	0.042861193	
	25 years and older	0.046489093	
Additional CMHC per capita per week increase per year by age AIHW	0–4 years	-0.000243268	Estimated with constrained optimisation using mental health service engagement data from the ABS [20], mental health perfomance indicator data from AIHW [52], Medicare-subsidised primary mental health services data from AIHW [50], CMHC service contacts data from AIHW [51], headspace occasions of service data provided by BSPHN, mental health related ED presentations data provided by Queensland Health [48] and episodes of admitted care data provided by Queensland Health [48].
	5–11 years	0.000392489	
	12-17 years	0.002132025	
	18-24 years	0.000638724	
	25 years and older	-0.001044554	
Waiting for CMHC initial total	35055.52948		Estimated with constrained optimisation using mental health service engagement data from the ABS [20], mental health perfomance indicator data from AIHW [52], Medicare-subsidised primary mental health services data from AIHW [50], CMHC service contacts data from AIHW [51], headspace occasions of service data provided by BSPHN, mental health related ED presentations data provided by Queensland Health [48] and episodes of admitted care data provided by Queensland Health [48].
Seeking help headspace rate by age 12+ initial	12-14 years	0.000001012955	Estimated with constrained optimisation using mental health service engagement data from the ABS [20], mental health perfomance indicator data from AIHW [52], Medicare-subsidised primary mental health services data from AIHW [50], CMHC service contacts data from AIHW [51], headspace occasions of service data provided by BSPHN, mental
	15-17 years	0.000001331976	
	18-24 years	0.000000968132	
	25 years and older	0.000004073255	

			health related ED presentations data provided by Queensland Health [48] and episodes of admitted care data provided by Queensland Health [48].
Seeking help headspace rate by age 12+ increase per year	12-14 years	0.000509271129	Estimated with constrained optimisation using mental health service engagement data from the ABS [20], mental health perfomance indicator data from AIHW [52], Medicare-subsidised primary mental health services data from AIHW [50], CMHC service contacts data from AIHW [51], headspace occasions of service data provided by BSPHN, mental health related ED presentations data provided by Queensland Health [48] and episodes of admitted care data provided by Queensland Health [48].
	15-17 years	0.000408177488	
	18-24 years	0.000215692733	
	25 years and older	-0.000000055495	
Waiting for headspace initial total	0		Estimated with constrained optimisation using mental health service engagement data from the ABS [20], mental health perfomance indicator data from AIHW [52], Medicare-subsidised primary mental health services data from AIHW [50], CMHC service contacts data from AIHW [51], headspace occasions of service data provided by BSPHN, mental health related ED presentations data provided by Queensland Health [48] and episodes of admitted care data provided by Queensland Health [48].
**Social cohesion**			
Social cohesion initial	92.5068294842		Estimated with constrained optimisation using national social cohesion data from the Scanlon Foundation Research Institute [49]
Social cohesion change per year	-0.788470472722		Estimated with constrained optimisation using national social cohesion data from the Scanlon Foundation Research Institute [49]

Acknowledgements

The authors acknowledge the staff of the Statistical Services Branch, the Mental Health Alcohol and Other Drugs Branch, and the Hospital Access Analysis Team from Queensland Health for the datasets used for this research.

References

1. Australian Department of Health and Aged Care. *Primary Health Networks (PHNs) collection of concordance files*. 2023; Available from: https://www.health.gov.au/resources/collections/primary-health-networks-phns-collection-of-concordance-files.
2. Public Health Information Development Unit. *Social Health Atlas of Australia: Population Health Networks*. 2016-2021; Available from: https://phidu.torrens.edu.au/social-health-atlases/data#social-health-atlas-of-australia-primary-health-networks.
3. Australian Bureau of Statistics. *Population: Births, Australia*. 2021; Available from: https://www.abs.gov.au/statistics/people/population/births-australia.
4. Australian Bureau of Statistics. *Population: Deaths, Australia*. 2021; Available from: https://www.abs.gov.au/statistics/people/population/deaths-australia/.
5. Australian Bureau of Statistics. *Regional Statistics, ASGS 2016 [Data Explorer]*. 2020; Available from: https://explore.data.abs.gov.au/.
6. Butterworth, P. and L.S. Leach, *Early Onset of Distress Disorders and High-School Dropout: Prospective Evidence From a National Cohort of Australian Adolescents.* American Journal of Epidemiology, 2017. **187**(6): p. 1192-1198.
7. Lee, S., et al., *Mental disorders and termination of education in high-income and low- and middle-income countries: epidemiological study.* The British Journal of Psychiatry, 2009. **194**(5): p. 411-7.
8. Australian Curriculum Assessment and Reporting Authority, *Enrolments by Grade 2008-2021*. 2023.
9. Australian Curriculum Assessment and Reporting Authority, *Senior Secondary Outcomes 2008-2020*. 2023.
10. Queensland Department of Education. *Next Step post-school destination surveys: Year 12 Completers survey*. 2023 1 March 2023]; Available from: https://qed.qld.gov.au/publications/reports/statistics/schooling/learning-outcomes/next-step/year-12-completers.
11. Australian Bureau of Statistics. *Education and Work [Tablebuilder]*. 2014-2022; Available from: https://www.abs.gov.au/statistics/microdata-tablebuilder/tablebuilder.
12. Welsh, J., et al., *Education-related inequalities in cause-specific mortality: first estimates for Australia using individual-level linked census and mortality data.* International Journal of Epidemiology, 2021. **50**(6): p. 1981-1994.
13. Sorlie, P.D. and E. Rogot, *Mortality by employment status in the National Longitudinal Mortality Study.* American Journal of Epidemiology, 1990. **132**(5): p. 983-92.
14. Frijters, P., D.W. Johnston, and M.A. Shields, *The effect of mental health on employment: Evidence from Australian Panel Data.* Health Economics, 2014. **23**(9): p. 1058-1071.
15. Australian Bureau of Statistics. *Microdata: National Health Survey [Microdata Download]*. 2011-12, 2014-15, 2017-18; Available from: https://www.abs.gov.au/statistics/microdata-tablebuilder/microdatadownload.
16. Wilkins, R., *The Extent and Consequences of Underemployment in Australia.* Melbourne Institute of Applied Economic and Social Research, The University of Melbourne, Melbourne Institute Working Paper Series, 2004.
17. Australian Bureau of Statistics. *Labour Force, Australia, Detailed*. 2023; Available from: https://www.abs.gov.au/statistics/labour/employment-and-unemployment/labour-force-australia-detailed/.
18. Australian Bureau of Statistics. *Labour Force, Australia*. 2023; Available from: https://www.abs.gov.au/statistics/labour/employment-and-unemployment/labour-force-australia/.
19. Kessler, R.C., et al., *Short screening scales to monitor population prevalences and trends in non-specific psychological distress.* Psychol Med, 2002. **32**(6): p. 959-76.

20. Australian Bureau of Statistics. *Microdata: National Study of Mental Health and Wellbeing [Microdata Download]*. 2007; Available from: https://www.abs.gov.au/statistics/microdata-tablebuilder/microdatadownload.
21. Dooley, D., J. Prause, and K.A. Ham-Rowbottom, *Underemployment and Depression: Longitudinal Relationships.* Journal of Health and Social Behavior, 2000. **41**(4): p. 421-436.
22. Griffiths, D., et al., *The Impact of Work Loss on Mental and Physical Health During the COVID-19 Pandemic: Baseline Findings from a Prospective Cohort Study.* Journal of Occupational Rehabilitation, 2021. **31**(3): p. 455-462.
23. Marmorstein, N.R., W.G. Iacono, and S.M. Malone, *Longitudinal associations between depression and substance dependence from adolescence through early adulthood.* Drug and Alcohol Dependence, 2010. **107**(2): p. 154-160.
24. Australian Bureau of Statistics. *Information paper. Use of the Kessler psychological distress scale in ABS health surveys, Australia, 2007-08. Cat. no. 4817.0.55.001*. 2012; Available from: https://www.abs.gov.au/AUSSTATS/abs@.nsf/DetailsPage/4817.0.55.0012007-08?OpenDocument.
25. Russ, T.C., et al., *Association between psychological distress and mortality: individual participant pooled analysis of 10 prospective cohort studies.* BMJ : British Medical Journal, 2012. **345**: p. e4933.
26. Australian Bureau of Statistics. *National Health Survey [Tablebuilder]*. 2020-21; Available from: https://www.abs.gov.au/statistics/microdata-tablebuilder/tablebuilder.
27. Australian Bureau of Statistics. *National Health Survey [TableBuilder]*. 2011-12, 2013-14, 2017-18; Available from: https://www.abs.gov.au/statistics/microdata-tablebuilder/tablebuilder.
28. Young Minds Matter Survey. *Survey Results Query Tool [online]*. 2013-14; Available from: http://www.youngmindsmatterresults.org.au/PrevalenceK10BS.html.
29. Australian Bureau of Statistics. *National Study of Mental Health and Wellbeing*. 2007; Available from: https://www.abs.gov.au/statistics/health/mental-health/national-study-mental-health-and-wellbeing/2007.
30. Australian Bureau of Statistics. *National Study of Mental Health and Wellbeing*. 2020-22; Available from: https://www.abs.gov.au/statistics/health/mental-health/national-study-mental-health-and-wellbeing/2020-2022.
31. Australian Institute of Family Studies. *Growing Up in Australia: Longitudinal Study of Australian Children (LSAC) Release 9.1 C2 (Waves 1-9C) [ADA Dataverse]*. 2022; Available from: https://growingupinaustralia.gov.au/.
32. Morrison, D.S., *Homelessness as an independent risk factor for mortality: results from a retrospective cohort study.* International Journal of Epidemiology, 2009. **38**(3): p. 877-883.
33. Nilsson, S.F., M. Nordentoft, and C. Hjorthøj, *Individual-Level Predictors for Becoming Homeless and Exiting Homelessness: a Systematic Review and Meta-analysis.* Journal of Urban Health, 2019. **96**(5): p. 741-750.
34. Ranney, K. *Brisbane Zero reduces the average length of time a person experiences homelessness by 40% with the help of real-time, by-name data*. 2023; Available from: https://community.solutions/case-studies/brisbane-zero-reduces-the-average-length-of-time-a-person-experiences-homelessness-by-40-with-the-help-of-real-time-by-name-data/.
35. Australian Bureau of Statistics, *Estimating Homelessness: Census*. 2011, 2016, 2021, ABS: Canberra.
36. Australian Bureau of Statistics. *Census of Population and Housing [TableBuilder]*. 2016, 2021; Available from: https://www.abs.gov.au/statistics/microdata-tablebuilder/tablebuilder.
37. Roerecke, M. and J. Rehm, *Alcohol use disorders and mortality: a systematic review and meta-analysis.* Addiction, 2013. **108**(9): p. 1562-1578.
38. Australian Institute of Health and Welfare. *National Drug Strategy Household Survey 2016, ADA Dataverse, V8*. 2017; Available from: https://dataverse.ada.edu.au/dataverse.
39. Johnson, T., et al., *Substance Abuse and Homelessness: Social Selection or Social Adaptation?* Addiction, 1997. **92**: p. 437-445.
40. Slade, T., et al., *The Mental Health of Australians 2: Report on the 2007 National Survey of Mental Health and Wellbeing*. 2009.
41. Gariépy, G., et al., *The mental health of young people who are not in education, employment, or training: a systematic review and meta-analysis.* Social Psychiatry and Psychiatric Epidemiology, 2022. **57**(6): p. 1107-1121.
42. Brisbane South Primary Health Network. *Mental Health and Alcohol and Other Drugs Service Mapping Project: Final Report Alcohol and Other Drugs*. 2016; Available from: https://bsphn.org.au/wp-content/uploads/2017/12/BSPHN-Mental-Health-and-AOD-Service-Mapping-Final-Report-Alcohol-and-Other-Drugs-October-2016.pdf.
43. Australian Institute of Health and Welfare. *Alcohol and other drug treatment services in Australia: Primary Health Network (PHN) Data*. 2016-21; Available from:

https://www.aihw.gov.au/getmedia/bb441e32-ea60-4851-900b-acae743b9944/aihw-hse-250-2021-SCR-client-rates-and-multiple-years-27072022.xls.aspx.
44. Australian Institute of Health and Welfare. *Alcohol and other drug treatment services in Australia annual report: Duration of treatment*. 2023; Available from: https://www.aihw.gov.au/reports/alcohol-other-drug-treatment-services/alcohol-other-drug-treatment-services-australia/contents/treatment-referral-completion/referral-to-treatment.
45. Hockey, M., et al., *Psychological distress as a risk factor for all-cause, chronic disease- and suicide-specific mortality: a prospective analysis using data from the National Health Interview Survey.* Social Psychiatry and Psychiatric Epidemiology, 2022. **57**(3): p. 541-552.
46. Too, L.S., et al., *The association between mental disorders and suicide: A systematic review and meta-analysis of record linkage studies.* Journal of Affective Disorders, 2019. **259**: p. 302-313.
47. Australian Institute of Health and Welfare, *Suicide and self-harm monitoring*. 2021: Canberra.
48. Statistical Services Branch (Queensland Health). 2023; Available from: https://www.health.qld.gov.au/hsu/how-to-access-data-from-ssb.
49. Scanlon Foundation Research Institute. *Scanlon-Monash Index*. 2023; Available from: https://scanloninstitute.org.au/research/scanlon-monash-index.
50. Australian Institute of Health and Welfare. *Medicare-subsidised GP, allied health and specialist health care across local areas: 2021–22*. 2022; Available from: https://www.aihw.gov.au/reports/primary-health-care/medicare-subsidised-gp-allied-health-and-specialis/data.
51. Australian Institute of Health and Welfare. *Mental health services in Australia: State and territory community mental health services*. 2017-18; Available from: https://www.aihw.gov.au/mental-health/topic-areas/community-services.
52. Australian Institute of Health and Welfare. *Mental health performance indicators [online]*. 2021; Available from: https://www.aihw.gov.au/mental-health/monitoring/performance-indicators#apmhs.
53. Australian Bureau of Statistics. *Deaths, Year of occurrence, Age at death, Age-specific death rates, Sex, States, Territories and Australia [Data Explorer], Deaths, Year of occurrence, Age at death, Age-specific death rates, Sex, States, Territories and Australia [Data Explorer]*. 2022 16 March 2023]; Available from: https://explore.data.abs.gov.au/.
54. Australian Curriculum Assessment and Reporting Authority. *Enrolments by Grade 2008-2021*. Available from: https://acara.edu.au/contact-us/acara-data-access.
55. Australian Curriculum Assessment and Reporting Authority. *Senior Secondary Outcomes 2008-2020*. Available from: https://acara.edu.au/contact-us/acara-data-access.
56. Australian Bureau of Statistics. *Education: Schools [ABS Website]*. 2022 1 March 2023]; Available from: https://www.abs.gov.au/statistics/people/education/schools/2022.
57. Australian Bureau of Statistics. *Education and Work [Tablebuilder]*. 2015-2022; Available from: https://www.abs.gov.au/statistics/microdata-tablebuilder/tablebuilder.
58. Jokela, M., et al., *Natural course of recurrent psychological distress in adulthood.* Journal of Affective Disorders, 2011. **130**(3): p. 454-461.
59. Australian Bureau of Statistics, *National Survey of Mental Health and Wellbeing: Summary of Results, 2007*. 2008: Canberra.
60. Manning, V., et al., *Substance use outcomes following treatment: Findings from the Australian Patient Pathways Study.* Australian & New Zealand Journal of Psychiatry, 2017. **51**(2): p. 177-189.
61. Australian Institute of Health and Welfare. *Suicide and Self-harm Monitoring: National Mortality Database—Suicide (ICD-10 X60–X84, Y87.0)*. 2021; Available from: https://www.aihw.gov.au/suicide-self-harm-monitoring/data/suicide-self-harm-monitoring-data.
62. Australian Institute of Health and Welfare. *Suicide and Self-harm Monitoring: National Hospital Morbidity Database*. 2021; Available from: https://www.aihw.gov.au/suicide-self-harm-monitoring/data/suicide-self-harm-monitoring-data.
63. Tyrer, P., et al., *A randomised controlled study of close monitoring of vulnerable psychiatric patients.* The Lancet, 1995. **345**(8952): p. 756-759.
64. Australian Institute of Health and Welfare. *Mental health: Consumer outcomes in mental health care*. 2016-17; Available from: https://www.aihw.gov.au/mental-health/topic-areas/consumer-outcomes.
65. Australian Institute of Health and Welfare. *Mental health services in Australia: Overnight admitted mental health-related care*. 2021; Available from: https://www.aihw.gov.au/mental-health/topic-areas/admitted-patients.
66. Christensen, H., K.M. Griffiths, and A.F. Jorm, *Delivering interventions for depression by using the internet: randomised controlled trial.* BMJ, 2004. **328**(7434): p. 265.
67. KPMG. *Evaluation of the National headspace Program*. 2022; Available from: https://www.health.gov.au/sites/default/files/documents/2022/10/evaluation-of-the-national-headspace-program.pdf.

68. Australian Institute of Health and Welfare. *Mental health services in Australia — Mental health-related care in general practice*. 2015-16; Available from: https://www.aihw.gov.au/getmedia/86df866c-05c5-4d44-92c8-d35c3a36f18f/Mental-health-related-service-provided-by-general-practitioners-2015-16.xlsx.aspx.
69. Thase, M.E., et al., *Treatment of Major Depression With Psychotherapy or Psychotherapy-Pharmacotherapy Combinations.* Archives of General Psychiatry, 1997. **54**(11): p. 1009-1015.
70. Australian Institute of Health and Welfare. *Mental health: Mental health services*. 2016; Available from: https://www.aihw.gov.au/mental-health/overview/mental-health-services.
71. Cuijpers, P., et al., *Screening for mood and anxiety disorders with the five-item, the three-item, and the two-item Mental Health Inventory.* Psychiatry Research, 2009. **168**(3): p. 250-255.
72. Cuijpers, P., et al., *Psychological treatment of depression in primary care: a meta-analysis.* British Journal of General Practice, 2009. **59**(559): p. e51-e60.
