# Supplementary material for: Beyond capacity limits: can social cohesion offset the impact of service constraints on youth mental health?
Source: Eur Psychiatry. 2025 Jun 27;68(1):e93. doi: 10.1192/j.eurpsy.2025.10053 (PMC12260717; doi:10.1192/j.eurpsy.2025.10053)
Supplement: Occhipinti et al. supplementary material 2 — Occhipinti et al. supplementary material [file S0924933825100539sup002.pdf]

Supplementary file 2: Heatmaps

Beyond capacity limits: Can social cohesion offset the impact of service constraints on youth mental health?

Jo-An Occhipinti, PhD,[a,b,*§] Nicholas Ho, BSc. Hons,[a§] Paul Crosland, MHEcon,[a] Sam Huntley, BA(Psych),[a] Wendy Hawkins, BOccThy,[c] Adam Connell, MPH,[d] Judith Piccone, MClinPsych,[e] Sarah Piper, BA(Psych),[a] Seyed Hossein Hosseini, PhD,[a] Catherine Vacher, PhD,[a] Jordan Frith, BPH,[a] Sophie Morson, MClinPsych,[f] Courtney Milham, BHumanServ,[g] Wendy Burton, MD,[h] Kayla Andrade, BA(Arch),[a] Chloe Gosling, BSc (Psych Crim),[a] Kristen Tran, MEc,[a] Yun Ju C. Song, PhD,[a] Victoria Loblay, PhD,[a] Jo Robinson, PhD,[i,j] Adam Skinner, Ph,[a,^] Ian B. Hickie, MD.[a,^]

Affiliations:

[a] Brain and Mind Centre, Faculty of Medicine and Health, University of Sydney, Australia

[b] Computer Simulation & Advanced Research Technologies, Sydney, Australia.

[c] Metro South Hospital and Health Service, Queensland Health, Brisbane, Australia

[d] Mental Health Alcohol and Other Drugs Strategy and Planning Branch, Queensland Health, Brisbane, Australia

[e] Children's Health Queensland Hospital and Health Service; Brisbane, Australia

[f] Thriving Queensland Kids Partnership/Australian Research Alliance for Children and Youth; Brisbane, Australia

[g] Brisbane South Primary Health Network, Brisbane, Australia

[h] Morningside General Practice Clinic, Brisbane, Australia

[i] Orygen, Melbourne, Australia

[j] Centre for Youth Mental Health, The University of Melbourne, Melbourne, Australia

[§] Joint first authors

^ Joint senior authors

*Corresponding author: Jo-An Occhipinti, Head, Systems Modelling & Simulation, The Brain and Mind Centre, Faculty of Medicine & Health, University of Sydney, Moore College, Level 4, 1 King Street, Newtown, NSW, 4042, Australia; Ph: +61 467 522 766; email: jo-an.occhipinti@sydney.edu.au

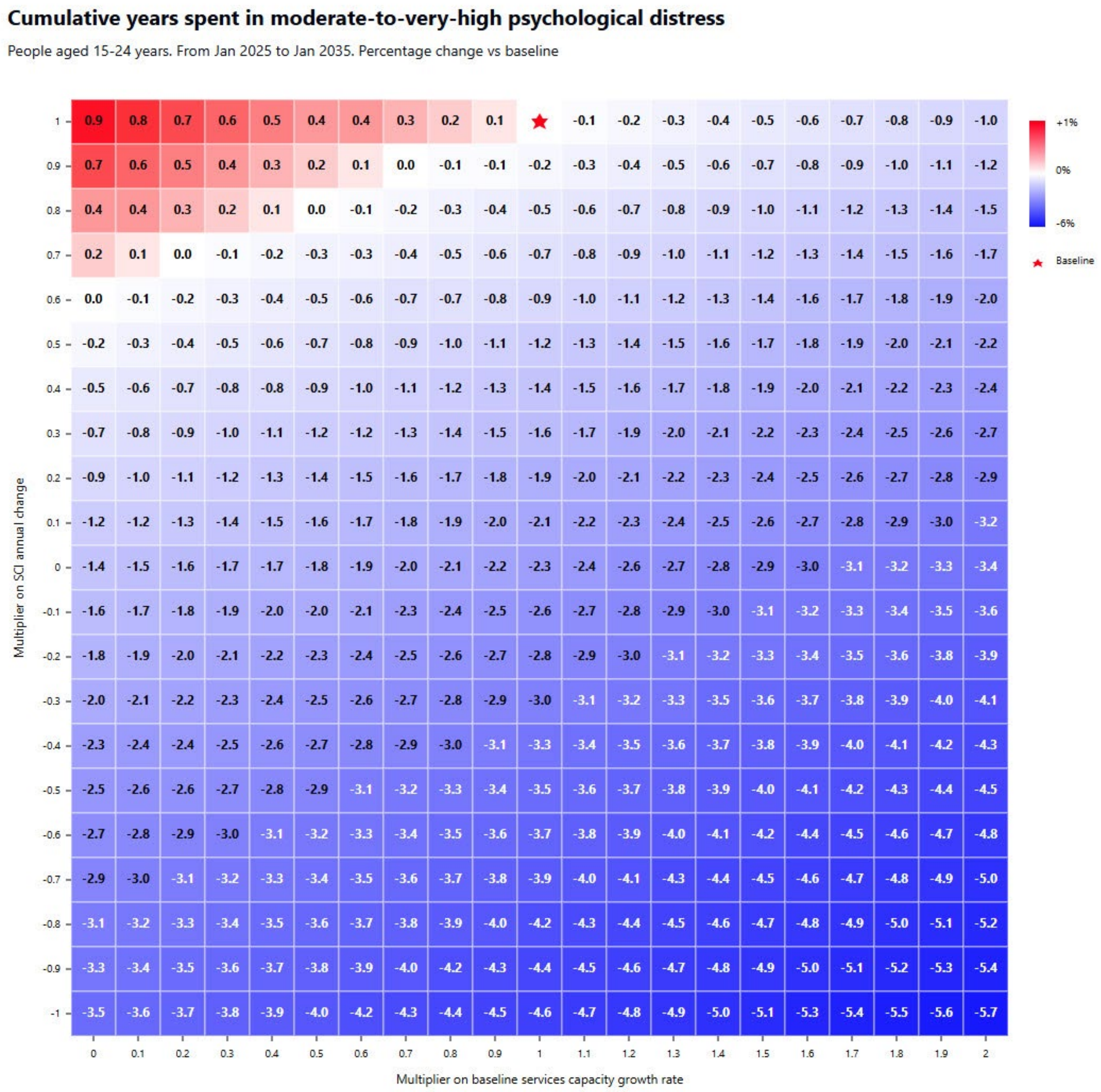

Fig. *S2a* Simulation results. Combined impact of changes to growth rate of specialised services, headspace and GP services capacity (x-axis) versus social cohesion (y-axis) on cumulative years spent in moderate-to-very-high psychological distress over the 10-year period, January 2025 to January 2035. The figure presented in each square represents the increase/decrease against the baseline. Red shading corresponds with a deterioration (increasing distress) and blue shading an improvement (decreasing distress) with the intensity of shading reflecting the scale of deterioration or improvement. The baseline services capacity growth rate and social cohesion is marked by the red star.

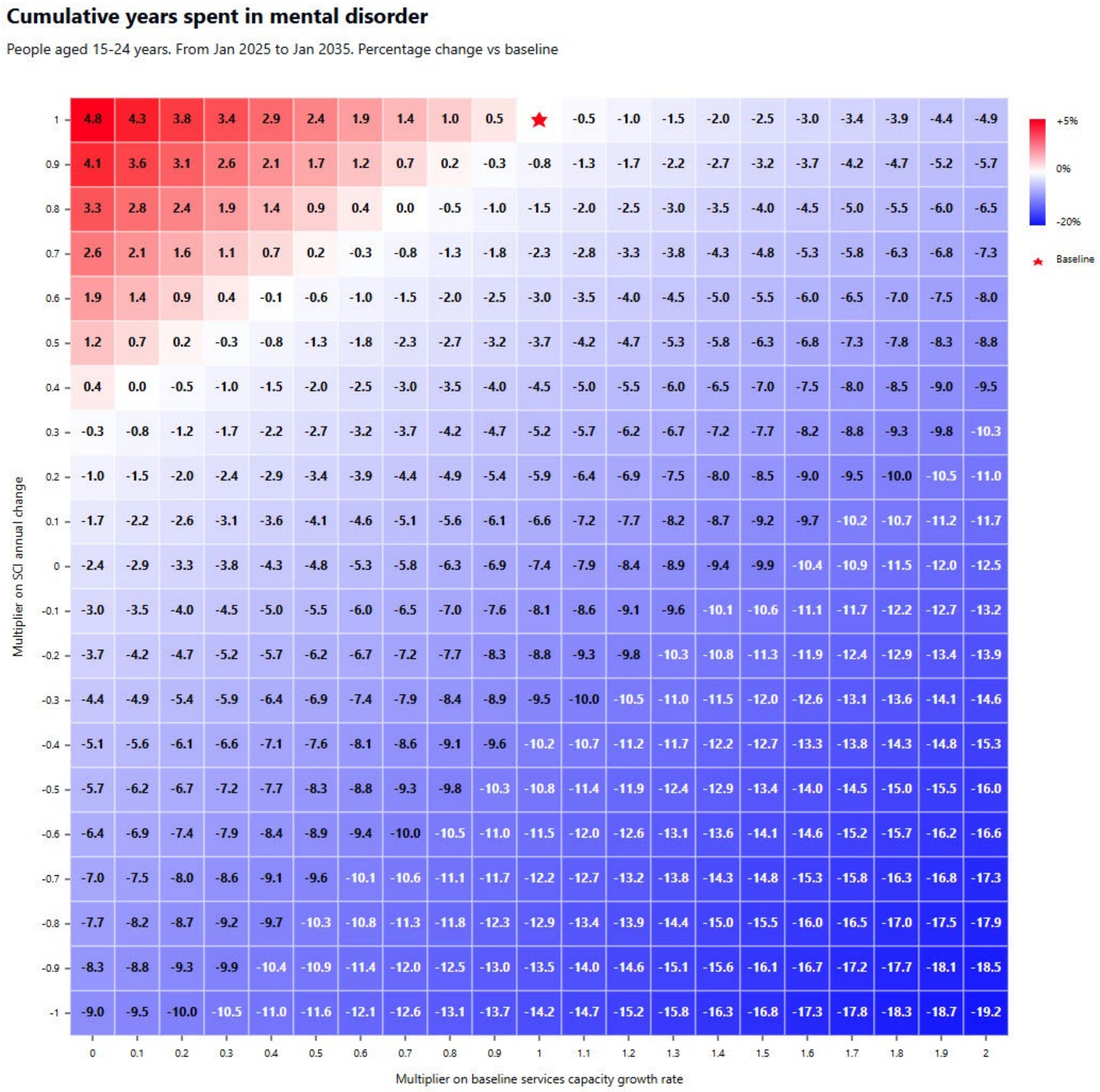

Fig. *S2b* Simulation results. Combined impact of changes to growth rate of specialised services, headspace and GP services capacity (x-axis) versus social cohesion (y-axis) on cumulative years spent in mental disorder over the 10-year period, January 2025 to January 2035. The figure presented in each square represents the increase/decrease against the baseline. Red shading corresponds with a deterioration (increasing disorder) and blue shading an improvement (decreasing disorder) with the intensity of shading reflecting the scale of deterioration or improvement. The baseline services capacity growth rate and social cohesion is marked by the red star.
